# MET Exon 14 Splice-Site Mutations Preferentially Activate KRAS Signaling to Drive Tumourigenesis

**DOI:** 10.3390/cancers14061378

**Published:** 2022-03-08

**Authors:** Daniel Lu, Amy Nagelberg, Justine LM Chow, Yankuan T Chen, Quentin Michalchuk, Romel Somwar, William W. Lockwood

**Affiliations:** 1 Department of Integrative Oncology, BC Cancer Research Institute, Vancouver, BC V5Z 1L3, Canada; daniel.lu@bccrc.ca (D.L.); anagelberg@bccrc.ca (A.N.); jchow@bccrc.ca (J.L.C.); terrych1@student.ubc.ca (Y.T.C.); qmichalchuk@bccrc.ca (Q.M.); 2 Department of Interdisciplinary Oncology, University of British Columbia, Vancouver, BC V5Z 1L3, Canada; 3 Department of Pathology & Laboratory Medicine, University of British Columbia, Vancouver, BC V6T 1Z7, Canada; 4 Department of Pathology, Memorial Sloan-Kettering Cancer Center, New York, NY 10065, USA; somwarr@mskcc.org; 5 Human Oncology and Pathogenesis Program, Memorial Sloan-Kettering Cancer Center, New York, NY 10065, USA

**Keywords:** lung cancer, targeted therapies, Hepatocyte Growth Factor Receptor, splice-site mutations, RAS

## Abstract

**Simple Summary:**

*MET* exon 14 splice-site mutations occur in ~3–4% of lung adenocarcinoma cases, defining a cohort of patients which might benefit from anti-MET targeted therapy. Such therapies have yielded mixed results, however, pointing to the need for better treatment design. Our study sought to aid this by characterizing key changes in mutant MET signaling behaviour. We first compared the transcriptional profiles of lung tumours with either *METΔex14* or wild-type *MET*-amplification. *METΔex14*-mutant tumours exhibited increased activation of the Ras-MAPK pathway, consistent with our observations in an isogenic model system. Furthermore, sustained activity of this pathway is necessary for proliferation and maintenance of *METΔex14* tumours, while forced reactivation of this pathway is sufficient to restore growth in the absence of MET activity. Our findings suggest that the MAPK pathway represents a main effector of *METΔex14*-driven cancer, lending credence to the possibility of combined MET-MAPK inhibition to improve therapeutic outcomes.

**Abstract:**

Targeted therapies for *MET* exon 14-skipping (*METΔex14*)-driven lung cancers have generated some promising results but response rates remain below that seen for other kinase-driven cancers. One strategy for improving treatment outcomes is to employ rational combination therapies to enhance the suppression of tumour growth and delay or prevent the emergence of resistance. To this end, we profiled the transcriptomes of MET-addicted lung tumours and cell lines and identified the RAS-mitogen-activated protein kinase (MAPK) pathway as a critical effector required for METΔex14-dependent growth. Ectopic expression of *MET* in an isogenic cell line model showed that overexpression of the mutant MET receptor led to higher levels of MAPK phosphorylation and nuclear import, resulting in increased expression and phosphorylation of nuclear MAPK targets. In comparison, other known MET effectors were unaffected. Inhibition of this pathway by *KRAS* knockdown in MET-addicted cells in vitro led to decreased viability in only the *METΔex14*-mutant cells. Conversely, decoupling RAS-MAPK axis, but not other effector pathways, from MET activity via the introduction of constitutively active mutants conferred resistance to MET inhibitors in vitro. Our results suggest that aberrant hyperactivity of the MET receptor caused by the exon 14-skipping mutation does not uniformly upregulate all known downstream effectors, rather gaining a predilection for aberrantly activating and subsequently relying on the RAS-MAPK pathway. These findings provide a rationale for the co-targeting of the RAS-MAPK pathway alongside MET to prolong therapeutic response and circumvent resistance to improve patient survival.

## 1. Introduction

The advent of improved sequencing technologies over the past decade has led to routine detection of actionable alterations becoming standard-of-care in patients with non-small cell lung cancer (NSCLC). Tumours harbouring mutations in genes such as *EGFR* [1,2,3] and *ERBB2* [4,5], or gene rearrangements involving *ALK* [6,7], *RET* [8], or *ROS1* [9] are typically dependent on the oncogene for survival, with profound anti-tumour effects observed in response to the appropriate targeted therapy. Frequently found to be overexpressed or amplified in lung cancers, aberrant activation of the Hepatocyte Growth Factor (MET) receptor typically co-occurs with other established oncogenic targets [10]. However, recent findings demonstrating that activating mutations in the *MET* gene occur in mutual exclusivity with mutations in other oncogenes have led to intensified interest in its potential as a therapeutic target [11,12].

As part of the receptor tyrosine kinase (RTK) family, MET plays a highly versatile role in the cell by functioning to integrate growth, survival, and signaling cues from the extracellular environment. This role necessitates the use of diverse signaling pathways, tightly regulated via dedicated signal transducers, adaptors, and scaffolding proteins to collectively modulate receptor activity and signal transduction [13]. Importantly, somatic mutations in the *MET* gene leading to loss of exon 14 (*METΔex14*) is a recurrent event in ~3–4% of NSCLC cases [12]. These mutations result in loss of the juxtamembrane (JM) regulatory domain of the receptor, impairing the cell’s ability to ubiquitylate and shut off MET receptor activity, leading to oncogenesis [14].

MET has long been known to be a critical mediator in processes ranging from foetal organogenesis during development, to tissue regeneration and inflammation reduction during wound healing [15,16,17]. Its oncogenic potential was also noted decades ago with the identification of TPR-MET fusion proteins in chemically transformed carcinoma cell lines [18]. However, it was the recent NGS-based discovery of recurrent splice-site mutations in *MET*, leading to oncogenic loss of exon14 in LUAD patients, which has generated interest in its potential as a candidate for targeted therapy [12,19]. MET-targeted therapies using repurposed inhibitors have met with some initial success in the clinic; however, unlike cancers driven by other oncogenes such as *EGFR*, response rates in *METΔex14* mutant tumours are lower [20,21,22]. In addition, as in other instances of targeted therapy, long-term patient survival remains poor due to acquired drug resistance [23,24,25]. A viable strategy for enhancing initial and long-term response is to design combinatorial therapies that target both the primary oncoprotein and its key effector(s) as first-line treatment. This strategy of “vertical inhibition” has been deployed with some success in BRAF^V600E/K^-positive melanoma patients, resulting in some improvements to overall response and progression-free survival [26,27,28]. In order to design similar therapies targeting RTK-driven cancers, identifying the crucial effector pathways responsible for maintaining oncogene addiction becomes a necessary pre-requisite. Given the relatively novel discovery of *MET* exon 14-skipping mutations in LUAD, there is a lack of insight towards how the *METΔex14* oncogene aberrantly engages its parallel downstream signaling pathways, especially compared to its role when amplified in other oncogene-driven cancers. In this study, we show that *MET* exon 14-skipping mutations specifically enhance signaling through the RAS/MAPK pathway, providing a potential avenue of combinatorial therapy to enhance patient survival.

## 2. Materials and Methods

### 2.1. Cell Lines and Culture Conditions

Cell lines were obtained from American Type Culture Collection (ATCC, Manassas, VA, USA) and cultured according to ATCC guidelines. Hs746T, H1993, and HEK293T cells were maintained in DMEM (Gibco, cat #12430-062, Waltham, MA, USA) supplemented with 10% (*v/v*) foetal bovine serum (FBS) (Gibco, cat #26140079) and 1% (*v/v*) Glutamax (Gibco, cat #250-30081). H596 and HPL1D cells were maintained in RPMI-1640 (Gibco, cat# 11875119) supplemented with 10% FBS and 1% Glutamax. Cultured cell lines were incubated at 37 °C in a humidified incubator infused with 5% CO_2_. All lines were maintained in 10 cm tissue culture dishes (Sarstedt, cat #83-3902, Nümbrecht, Germany) and propagated when approaching 75–85% confluency using 0.25% Trypsin-EDTA (Gibco, cat #25200114). All cell lines were tested regularly for mycoplasma by polymerase chain reaction as previously described [29]. All cell lines were authenticated by the supplier and once received were subcultured and stocks frozen. A new vial was thawed and used every 20–25 passages.

### 2.2. Generation of Plasmids, Lentiviruses, and Viral Transduction to Create Stable Expression Cells

pDONR223 PIK3CA_p.E545K (plasmid #82881), pDONR223 STAT3_p.A662C_N664C (plasmid #82184), and pDONR223 KRAS_p.G12D (plasmid #81651) were ordered from Addgene. Human MET_WT, MET_ΔEx14, and MET_p.Y1003F ORFs were subcloned into pCR8/GW/TOPO (Thermo Fisher Scientific, cat #K250020, Waltham, MA, USA) as we previously described [24]. Target genes were transferred by Gateway LR Clonase II enzyme mix (Thermo Scientific, cat #11791-020, Waltham, MA, USA) to pInducer20 (gift from S. Elledge, Harvard, Cambridge, MA, USA). Presence of insert was verified using PCR and restriction enzyme mapping. Lentivirus was generated by co-transfection of psPAX2 (Addgene, plasmid #12260, Watertown, MA, USA) and pMD2.G (Addgene, plasmid #12259) into HEK293T cells (ATCC) using Lipofectamine2000 (Thermo Scientific, cat #11668019). Lentiviral media containing 8 µg/mL hexadimethrine bromide/polybrene (MilliporeSigma, cat #H9268-10G, Burlington, MA, USA) was applied to target cells in Falcon 6-well plates (VWR, cat#353046, Radnor, PA, USA), followed by spinfection at 1200 G for 60 min. Target cells were maintained polyclonally in 800 µg/mL G-418 Sulphate (Gibco, cat #10131027) 48–72 h post-spinfection.

### 2.3. Doxycycline-Inducible shRNA and Overexpression Experiments

pTRIPZ:shKRAS and pTRIPZ:shScrambled plasmids were gifts from S. Dedhar, BC Cancer. Target cells virally transduced with a doxycycline-inducible expression system underwent antibiotic selection in 4 µg/mL puromycin and were subsequently maintained in their respective media containing 10% (*v/v*) Tet-system approved FBS (Takara Bio, cat #631101, Kyoto, Japan). Cells were maintained in 100–500 ng/mL doxycycline (Takara Bio, cat #631311) for 24–72 h prior to plating or harvest. Doxycycline-treated cells seeded for further assays were maintained in doxycycline until endpoint.

### 2.4. Growth Assays

Dose-response experiments were performed in 96-well flat-bottom plates (Sarstedt, cat #83-3924). To establish dose-response curves to various drugs, doxycycline-treated cells were seeded in quadruplicate at a density of 2000 cells per well and treated with the desired concentrations of chemicals. Viability was assayed 96 h following treatment using alamarBlue viability dye (Thermo Fisher Scientific, cat #A50100). Fluorescence of the dye (Ex: 560 nM, Em: 590 nM) following 2–4 h incubation was measured using a Cytation3 microplate reader (BioTek Instruments, Winooski, VT, USA). Curve-fitting of absorbance readings by non-linear regression with fitting by least sum of squares was performed using Graphpad Prism v8.4.3 to obtain IC_50_ values. Parameters calculated for each cell line were compared to control parameters by Extra sum-of-squares F-test. *p*-values < 0.05 were considered statistically significant. Long-term growth assays were performed in six-well flat bottom plates (Sarstedt, cat #83-392). Doxycycline-treated cells were seeded in triplicate at a density of 10,000–20,000 cells per well, based on growth rate, in media containing the desired concentrations of chemicals. Media was refreshed twice weekly for 14 days, following which cells were fixed with 100% methanol (Fisher Scientific, cat #A433P-4, Pittsburgh, PA, USA) and stained with 1:1 DPBS: Crystal Violet solution (MilliporeSigma, cat #HT90132-1L, Burlington, MA, USA). Cell growth was assessed using alamarBlue and by destaining CV-stained plates using 10% acetic acid and transferring the resulting solution into 96-well plates. The absorbance readings (Ex: 560 nM, Em: 590 nM) were measured to compare relative cell growth between conditions.

### 2.5. RNA Extraction and Microarray Analysis

H1993, Hs746T, and H596 cells were seeded in six-well plates (150,000 cells/well) and incubated overnight. Each cell line, in triplicate, was subjected to 6 h incubation under three different conditions: 0.1% DMSO (baseline), 0.1% DMSO + 25 ng/mL HGF (stimulatory), or 10 µM Cabozantinib + 25 ng/mL HGF (inhibitory). Cells were lysed in 300 µL RNA lysis buffer at room temperature and RNA purified using the Zymo Research Quick-RNA Miniprep kit (Cedarlane, cat #R1055, Burlington, ON, Canada). The RNA extraction, DNase I treatment, and column purification protocol was followed according to the manufacturer’s instructions. RNA concentration was measured using the Qubit 2.0 RNA HS Assay kit (Thermo Scientific, cat #32852). Samples were sent to The Centre for Applied Genomics (TCAG, Toronto, Canada), where RNA integrity, sample labelling, array hybridisation, and data acquisition steps were performed, using the GeneChip Human Gene 2.0 ST Array (Thermo Fisher Scientific, cat #902113, Waltham, MA, USA). Cross-array normalisation and background correction of raw expression values was performed using Robust Multiarray Analysis [30] to obtain triplicate expression values (in log_2_ scale) for each oligonucleotide probe. Annotation of gene names and symbols to each probeset was done on R using the “hugene20sttranscriptcluster.db” package available through Bioconductor [31]. Expression values for unmapped probes were dropped, while the average expression value for probes corresponding to the same gene was taken for each sample. Gene Set Enrichment Analysis (GSEA) [32,33] was used together with the “Oncogenic Signatures” library of curated gene sets from MSigDB, comparing gene expression profiles between MET_WT (H1993) and MET_ΔEx14 (Hs746T and H596) cells in the stimulatory state to identify top enriched gene sets. Gene Set Variation Analysis (GSVA) [34] was used to compare relative enrichment of KRAS-associated gene sets between cell lines and between stimulatory, inhibitory, and baseline conditions. Leading edge analysis was performed as previously described [33] to identify leading edge genes, and the R package ComplexHeatmap [35] was used to make all the heat maps. Individual differentially expressed (DE) genes were identified using the limma R [36] package available through Bioconductor. Benjamini–Hochberg-adjusted *p*-value > 0.05 and log_2_ (fold change) > 0 cut-offs were used to identify positive hits for all analyses.

### 2.6. Tumour RNA-Seq Expression Analyses

RNA-seq data and accompanying clinicopathological information (Supplementary Table S1) for 585 lung adenocarcinomas, of which 230 were profiled by The Cancer Genome Atlas [12], were downloaded from the NIH GDC Data Portal. Gene expression values were log_2_(x + 1)-normalised prior to analysis. Using the MSKCC cBioPortal, six tumours with wild-type *MET* amplification and eight tumours that harboured *MET* exon 14-skipping mutations were identified for expression analysis. GSVA was used to compare the relative enrichment of *KRAS* signature gene sets between tumours from the two groups.

### 2.7. Gene Ontology (GO) Analysis

GO [37] was used to determine the function of leading edge upregulated genes selected from gene sets associated with *KRAS*-driven cancers. The overrepresentation test of Protein Analysis Through Evolutionary Relationships (PANTHER; version 16.0) with Fisher’s Exact Test (FDR < 0.05 multiple test correction) was used to functionally classify the genes of interest, employing the PANTHER GO Biological Process annotation set [38,39]. Redundant GO terms were trimmed using REVIGO [40], and the remaining terms were visualised on R alongside fold change enrichment and FDR values as previously described [41].

### 2.8. Ras Activity Assay

The Active Ras Detection Kit (CST, cat #8821) was used to assess relative fractions of GTP-bound Ras in cells. The protocol for cell lysis and GST-RBD pulldown was followed according to the manufacturer’s instructions.

### 2.9. Protein Extraction and Western Blot Analysis

Cells were grown in 6-well, 6-cm, or 10-cm plates to 70–80% confluency. Prior to harvest, cells were washed with ice-cold PBS (Gibco, cat #14190250) and lysed with an appropriate volume of RIPA lysis buffer (G-Biosciences, cat #CA95029-284, St. Louis, MO, USA) containing Halt Protease and Phosphatase Inhibitor Mixture (Thermo Scientific, cat #PI78446). Cells were harvested by scraping and were frozen overnight at −80 °C. All samples were sonicated, then cleared of cell debris via centrifugation at 15,000× *g* for 15 min at 4 °C. Protein concentration was measured using the Pierce BCA Protein Assay kit (Thermo Scientific, cat #PI23225) according to the manufacturer’s instructions. Absorbance (Em: 562 nM) was measured using a microplate reader and sample protein concentration calculated using a set of BSA standards. In total, 30 µg of protein from each sample was mixed with NuPAGE LDS Sample Buffer (Thermo Scientific, cat #NP0008) and NuPAGE Sample Reducing Agent (Thermo Scientific, cat #NP0009) and denatured by boiling at 95 °C for 5 min. Samples were loaded into NuPAGE Novex 4–12% Bis Tris Gels (Thermo Scientific) alongside Precision Plus Protein Kaleidoscope Standards (Bio-Rad Laboratories, cat #161-0375, Hercules, CA, USA) and electrophoresed at 100 V for 2 h, then transferred to PVDF Immobilon membranes (MilliporeSigma, cat #IPVH00010, Burlington, MA, USA) at 110 V for 70 min. Membranes were blocked at room temperature for 1 h in 5% (*w/v*) bovine serum albumin (Sigma, cat #A7906-100 G) dissolved in TBS-T [1x Tris-Buffered Saline (Bio-Rad Laboratories, cat #170-6435), 0.1% (*v/v*) Tween-20 (Thermo Fisher Scientific, cat #BP337-500)]. Blocked membranes were incubated with primary antibodies (listed below) overnight at 4 °C with shaking. Membranes were then washed 3× with TBS-T and incubated with the appropriate horseradish peroxidase-conjugated secondary antibody at 1:10,000 dilution for 1 h at room temperature. SuperSignal West Pico Plus Chemiluminescent Substrate (Thermo Scientific, cat #PI-34580) and SuperSignal West Femto Chemiluminescent Substrate (Thermo Scientific, cat #PI-34096) were used on the ChemiDocMP Gel Imaging System (Bio-Rad Laboratories) to visualise immunoblot bands. Band intensity was quantified by densitometry using ImageJ software and summarized in Supplementary Table S2. Uncropped western blot images are collated as Supplementary Figure S6.

### 2.10. Antibodies, Inhibitors, and Other Reagents

All antibodies used for Western blot analysis were diluted in 5% *v/v* BSA in TBS-T prior to use. Primary antibodies were used at 1:1000 dilution, while secondary antibodies were used at 1:10,000 dilution. All antibodies except anti-ERM/ETV5 (Abcam, cat #ab102010, Cambridge, United Kingdom) and anti-DUSP6 (Santa Cruz Biotechnology, cat #sc-37707, Dallas, TX, USA) were purchased from Cell Signaling Technology (Danvers, MA, USA): MET (8198S), p-MET Tyr1234 (3077S), p-MET Tyr1349 (3121S), AKT (4691S)), p-AKT Ser473 (4060L), p44/42 MAPK (137F5), p-p44/42 MAPK Thr202/Tyr204 (9101S), MEK1/2 (9122S), p-MEK1/2 Ser217/221 (9121S), STAT3 (12640S), p-STAT3 Y705 (9145S), SRC (2108S), p-SRC Y416 (59548S), Ras (8955S), cMYC (5605S), cFOS (2250S), p-cFOS S32 (5348S), FRA1 (5281S), p-FRA1 S265 (5841S), Vinculin (13901S), β-Actin HRP-linked (12620S), Histone H3 (4499S), Anti-Rabbit IgG HRP linked (7074S), and Anti-Mouse IgG HRP linked (7076S). MET kinase inhibitors crizotinib (PF-02341066), cabozantinib (BMS-907351), and capmatinib (INCB28060) were purchased from SelleckChem (Houston, TX, USA) and dissolved in DMSO (Thermo Fisher Scientific, cat #BP231-100) prior to use. Hepatocyte Growth Factor recombinant human protein (PHG0324) was purchased from Thermo Scientific and reconstituted according to manufacturer’s instruction prior to use.

### 2.11. Xenograft Studies

All animal experiments were conducted in accordance with protocols approved by the University of British Columbia Animal Care Committee (UBC ACC). Cell lines used for the generation of tumour xenografts were as follows: H1993 pTRIPZ:shKRAS, H1993 pTRIPZ:shScrambled, Hs746T pTRIPZ:shKRAS, and Hs746T pTRIPZ:shScrambled. Immediately prior to injection, all cells were trypsinised, counted, and resuspended in an appropriate volume of ice-cold PBS. Moreover, 2 × 10^6^ pTRIPZ:shKRAS and pTRIPZ:shScrambled cells were injected subcutaneously into the left and right flanks of 8-week old male NRG mice (*n* = 16) in alternating fashion, with half the mice (*n* = 8) receiving H1993 cells and the other half receiving Hs746T cells. All mice were placed on doxycycline-infused feed (Envigo, cat #TD.130141, Indianapolis, IN, USA) starting 4 days prior to injection and continuing for the duration of the study. Measurement of tumour size was performed using callipers on a biweekly basis. Tumour volume was estimated using the equation V = 0.5 L·W^2^ and final weights were determined at endpoint.

## 3. Results

### 3.1. Cancer Cells Expressing METΔex14 Exhibit an Enhanced KRAS Activation Signature

**Figure 1:**
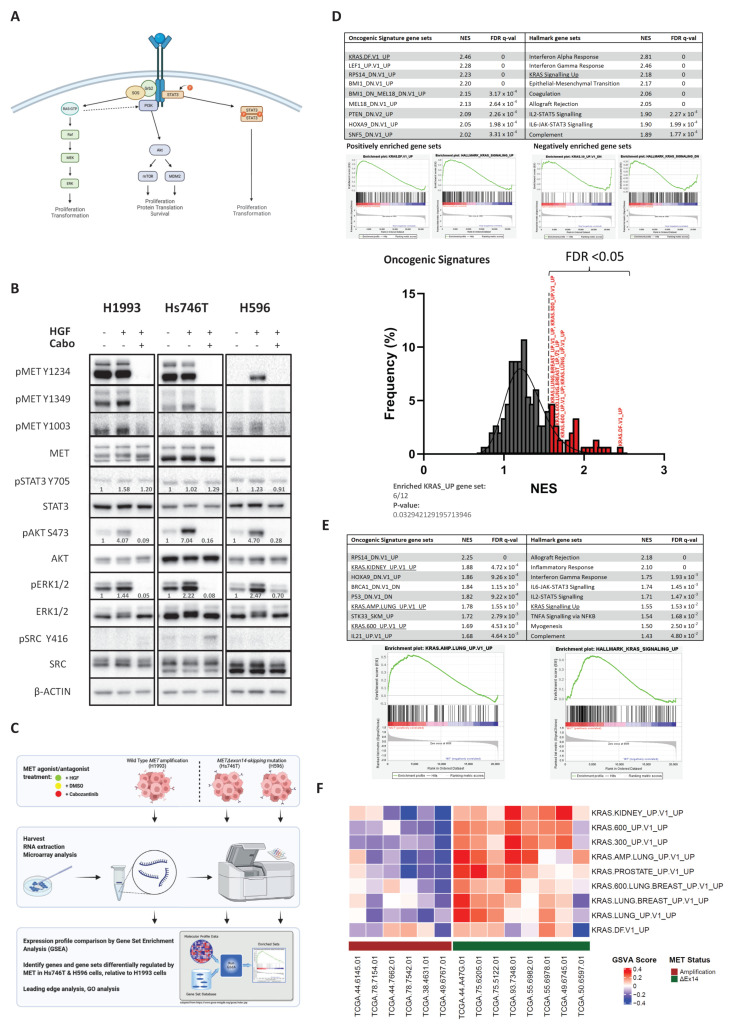
*METΔex14* variant-expressing cancers exhibit an enhanced KRAS activation signature. (**A**) When activated by HGF, the MET receptor is capable of triggering multiple downstream pathways in parallel. This is because its multisubstrate docking domain is capable of recruiting a diverse set of kinases and signaling factors. (**B**) WB analysis WT *MET*-amp (H1993) and *METΔex14*-expressing (Hs746T, H596) cells comparing relative phosphorylation levels of MET effector signaling pathways Ras-MAPK, PI3K-Akt, Stat3, and Src at baseline (0.1% DMSO; 30 min), MET-stimulated (25 ng/mL HGF + 0.1% DMSO; 30 min), or MET-inhibited (25 ng/mL HGF + 10 μM Cabozantinib; 30 min) states. (**C**) Expression profiling workflow: to identify transcriptomic differences in the molecular profiles of cancer cells addicted to WT MET-amp vs. METΔex14 activity, we performed Gene Set Enrichment Analysis (GSEA) comparing the expression profiles of Hs746T and H596 cells to that of H1993 cells (figure created using BioRender.com). (**D**) GSEA revealed that gene set expression signatures typically associated with KRAS-driven cancers are among the top-most enriched in *METΔex14*-expressing cancer lines when compared against the wild type *MET* cancer line, while gene sets downregulated in KRAS-driven cancers were similarly negatively enriched in *METΔex14*-expressing cancer lines. As multiple KRAS-related gene sets exist in the “Oncogenic_signatures” MSigDB library curated by UC San Diego and Broad Institute, Fisher’s Exact Test was performed to show significant overrepresentation of KRAS-associated gene sets (bottom right panel). (**E**) GSEA comparing TCGA RNA-seq data of *METΔex14*-expressing tumours with WT *MET*-amp tumours revealed similar enrichment of gene sets associated with KRAS-driven cancers. (**F**) Gene Set Variation Analysis (GVSA) of TCGA RNA-seq data from (**E**) showing relative enrichment of KRAS-associated gene sets (compared to all other gene sets in the “Oncogenic_signatures library) for WT *MET*-amp and in *METΔex14*-expressing tumours, at single-sample resolution. Heatmap shows positive relative enrichment of KRAS-associated gene sets in METΔex14-expressing lung tumours, and negative relative enrichment in WT *MET*-amp tumours.

Overexpression or low-level amplification of *MET* occurs in a significant fraction of LUADs, yet it represents a poor candidate for targeted monotherapy in these cases owing to its co-occurrence with alterations in other oncogenes [42]. In contrast, *MET* exon 14-skipping mutations, as well as high-level amplification of the wild type *MET* gene, constitute true driver alterations, which confer sensitivity to anti-MET therapy in some instances. However, while both receptor amplification and mutations leading to exon 14 skipping can result in aberrant hyperactivity of MET, they differ in the mechanism by which they stimulate receptor activity. There is evidence which suggests this may lead to differences in terms of receptor localisation, recycling, and protein interactions [43]. Thus, we wondered whether the downstream consequences on effector pathways might also differ depending on the route of hyperactivation, as this may impact the development of strategies for combination therapy (Figure 1A).

To determine whether such differences exist, we compared the status of known MET effector pathways in *METΔex14*-expressing cancer lines (Hs746T and H596) to another MET-addicted line with high-level amplification of the wild type *MET* gene (H1993). Notably, while all three lines exhibited a comparable degree of ERK1/2 (pERK1/2) and AKT (pAKT) phosphorylation at baseline, treatment with HGF to specifically stimulate MET activity resulted in a greater increase in phosphorylation of both RAS effectors in the *METΔex14* lines compared to H1993 cells, suggesting that stimulation of METΔex14 may activate these pathways to a greater degree than the wild-type receptor (Figure 1B). In contrast, the phosphorylation status of the RAS-independent effectors STAT3 and SRC remained at comparable levels regardless of treatment condition (Figure 1B). Furthermore, treatment with the MET inhibitor cabozantinib blocked the HGF-induced increase of pERK1/2 and pAKT, with no effects on pSTAT3 or SRC in the *METΔex14* cell lines. Our findings are consistent with recently published data. For example, phospho-kinase profiling *MET*-amplified (EBC-1) and *METΔex14* (Hs746T) cells similarly reported higher ERK1/2 phosphorylation levels in Hs746T cells compared to EBC-1 [44]. These results together suggest that, while all three cell lines are reliant on MET for growth and/or survival, *METΔex14*-expressing cells exhibit higher levels of HGF-induced RAS pathway activity despite similar or lower levels of MET phosphorylation.

To further explore the potential impact of these observed differences on downstream signaling pathways and transcriptional programs, we performed microarray analysis of RNA collected from Hs746T, H596, and H1993 cells following treatment with HGF (MET agonist), DMSO (baseline), or HGF+cabozantinib (MET inhibitor). We compared expression profiles between each cell line and assessed the resulting differentially expressed genes using gene set enrichment analysis (GSEA) to identify transcriptional programs differentially regulated between *METΔex14* and *MET*-amplified lines under conditions of MET stimulation, with a focus on the “Oncogenic Signatures” library of gene sets representing transcriptional signatures of cellular pathways known to be dysregulated in cancer (Figure 1C). This revealed that in comparison to *MET*-amplified cells, *METΔex14* cell lines specifically demonstrated enrichment of multiple *KRAS*-mediated gene sets corresponding to genes found upregulated in epithelial cancer cells expressing oncogenic KRAS (Figure 1D, bottom right panel: *p*-value = 0.033; Fisher’s Exact Test). We repeated this analysis using the alternate “Hallmarks” library gene sets, and observed a similar result. These results suggest that, compared to cancers addicted to the wild type MET receptor, METΔex14-driven cells exhibit an expression signature more similar to KRAS-driven cancers. Given the lack of *METΔex14* cell lines available, we expanded our analysis to include publicly available RNA-seq data [12] from tumours with either high-grade wildtype *MET* amplification (*n* = 6) or *METΔex14* mutations (*n* = 8). In agreement with our cell line findings, GSEA results show a positive enrichment of KRAS-mediated gene sets in the transcriptomes of *METΔex14*-mutant tumours compared to those with high-grade wildtype *MET* amplification (Figure 1E). Gene Set Variation Analysis (GSVA), which provide single-sample enrichment scores (ES) instead of calculating ES based on aggregate sample group gene expression Z-scores, showed that a diverse collection of KRAS-mediated gene sets was positively enriched in most *METΔex14*-expressing lung tumour samples while the same sets were negatively enriched in all wildtype *MET*-amplified lung tumour samples by comparison (Figure 1F). Furthermore, GSEA using positional gene sets found most genes in the Chr12Q15 region to be overexpressed in *METΔex14* tumour samples, suggesting focal amplification. Interestingly, this region encodes several *p53* pathway inhibitors, including *MDM2* and *YEATS4* [45,46] (Supplementary Figure S1). This finding is consistent with previous reports of concurrent *TP53* loss/*MDM2* amplification in *METΔex14* patient tumours [37].

### 3.2. METex14 Drives KRAS Signaling to a Greater Degree than the Wild-Type Receptor

**Figure 2:**
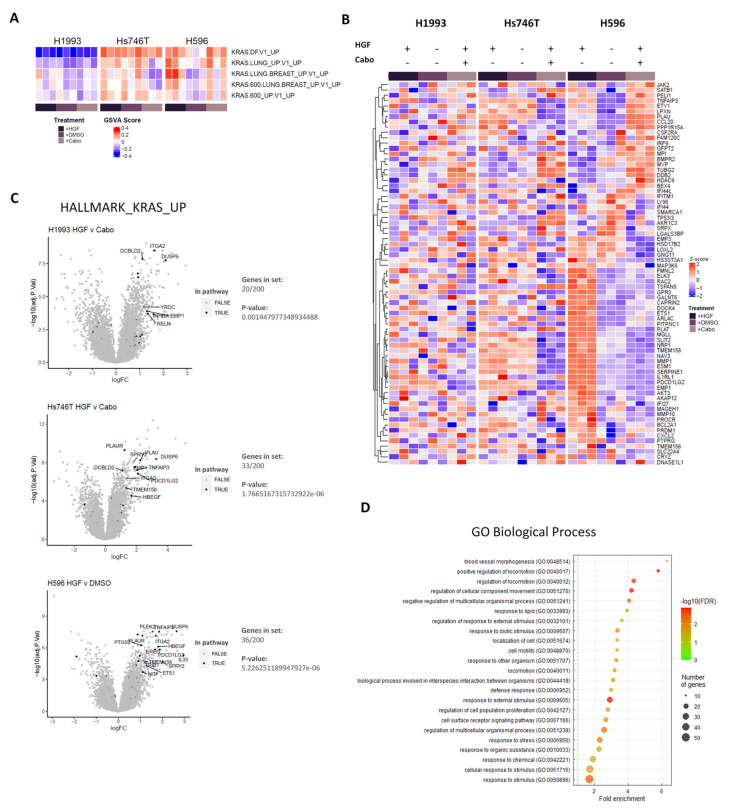
KRAS activation signature observed in exon14-skipping mutants is dependent on MET receptor activity. (**A**) GSVA comparing relative enrichment of KRAS-associated gene sets to NES of all gene sets from the entire Oncogenic_signatures library. Heatmaps show positive enrichment of Ras-driven genetic signatures in *METΔex14*-expressing cancer lines, in contrast to negative enrichment observed for the wild type *MET*-amp driven cell line. (**B**) Unsupervised clustering identifies a subset of genes within Ras-associated gene sets which correlate with MET receptor activity in *METΔex14*-expressing cancer lines, but not in the H1993 cell line. (**C**) Volcano plots comparing relative changes in gene expression of H1993, Hs746T, and H596 cells between HGF stimulated or MET inhibited states. Significantly upregulated hits (log2[FC] > 0; Adj *p*-value < 0.05) that fell under the “HALLMARKS_KRAS_UP” gene set were tallied for each line, and the significance of their overrepresentation calculated using Fisher’s Exact Test. (**D**) Gene ontology analysis of leading edge genes shown in (**B**,**C**) heatmaps point to their functional involvement in angiogenesis, cell mobility, and proliferation.

While GSEA identified enrichment of gene sets associated with mutant KRAS-driven cancers in both *METΔex14*-expressing tumours and cell lines, it is unknown whether the observed KRAS activation signature is a direct consequence of mutant MET activity, or whether it is a MET-independent phenomenon associated as a potential co-driver with *METΔex14*-presenting tumours. To investigate this, we sought to determine how the relative enrichment of the top 5 KRAS-associated gene sets (Figure 1D) changed in response to HGF or cabozantinib treatment using GSVA. While these gene sets are significantly more enriched in *METΔex14*-expressing lines H596 and Hs746T, we observed that they are further upregulated in response to HGF treatment (H596) and downregulated in response to cabozantinib treatment (Hs746T) (Figure 2A). This observation is consistent with the knowledge that, while Hs746T cells are addicted to active MET signaling with or without HGF, H596 cells show MET-driven growth only in the presence of HGF due to an activating mutation in the *PIK3CA* gene. Comparison of transcript levels for leading edge genes across treatment conditions revealed a positive correlation between their relative expression levels to MET receptor activity in *METΔex14* expressing cells. In contrast, we failed to observe any correlation of transcript levels to MET receptor activity in the wildtype *MET*-amplified cell line (Figure 2B). A parallel comparison using genes from the “HALLMARKS_KRAS_UP” set found that a larger fraction of these genes was significantly upregulated in *METΔex14*-expressing Hs746T (33/200 hits) and H596 (36/200 hits) cells in response to HGF stimulation, compared to the H1993 *MET*-amplified cell line (20/200) (Figure 2C). Some of these METΔex14-controlled genes include *SERPINE1*, *MMP1*, and *NRP-1*, known to promote metastatic and invasive behaviour, as well as promoters of growth and survival *ELK3*, *ETV1*, and *TMEM158*. Indeed, further GO analysis of the upregulated leading edge KRAS target genes revealed a critical involvement in functional processes associated with proliferative and invasive behaviour (e.g., regulation of cell migration and regulation of cell population proliferation) (Figure 2D). Interestingly, *PLAU* and *PLAT* were also upregulated in response to HGF treatment in Hs746T and H596 cells, respectively (Figure 2B); both genes code for plasminogen activators that are capable of cleaving environmental pro-HGF into its active form, suggesting a potential positive feedback mechanism promoting MET activity. Taken together, our data suggest that the activity of downstream KRAS pathway target genes, in particular those involved in regulating proliferative and metastatic behaviour, are regulated by MET receptor activity in splice mutant tumours in addition to being overexpressed relative to tumours with only *MET* amplification, a result that could be due to an apparent bias towards RAS-MAPK pathway activation in *METΔex14*-expressing cells.

### 3.3. METΔEx14-Expressing Cells Preferentially Hyperactivate the RAS-MAPK Pathway

**Figure 3:**
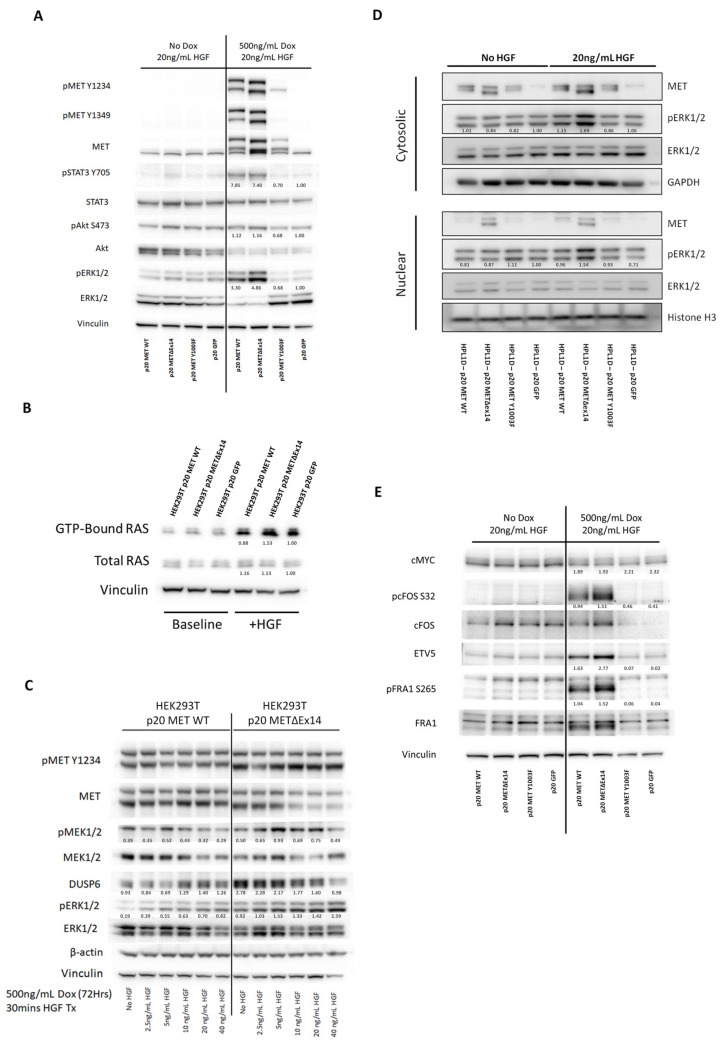
*METΔex14*-expressing cells preferentially hyperactivate the Ras-MAPK pathway. (**A**) Western blot analysis of major signaling effectors downstream of the MET receptor, including STAT3, Akt, and MAPK, show that all three pathways are upregulated in response to HGF treatment in *MET* WT or *METΔex14*-overexpressing cells, but that MAPK specifically exhibits higher phosphorylation levels in the METΔex14-expressing line. (**B**) GST-Raf-RBD pulldown demonstrates that HGF treatment (10 ng/mL) of METex14-expressing HEK293T cells results leads to a higher fraction of active, GTP-bound RAS compared to cells overexpressing wild type MET or GFP. (**C**) Western blot of Ras-MAPK pathway effectors in human embryonic kidney cells (HEK293T) ectopically expressing *MET* WT or *METΔex14* under the dox-inducible TetO promoter. Cells expressing *METΔex14* exhibit higher levels of MET receptor phosphorylation, as well as phosphorylation and activation of downstream Ras effectors MEK1/2 and ERK1/2. (**D**) Western blot analysis following separation of cytoplasmic and nuclear fractions show increased nuclear import of phosphorylated ERK1/2 in HGF-treated *MET* mutant-overexpressing cells, compared to *MET* WT or GFP-overexpressing cells. (**E**) Western blot analysis depicts METex14-expressing cells as exhibiting comparatively higher activating phosphorylation of select ERK1/2 nuclear targets known to promote growth, proliferation, or maintenance of alveolar type II cells.

Known downstream effectors of the MET receptor include the RAS-MAPK, PI3K-AKT, and JAK-STAT pathways (Figure 1A). On the basis of our transcriptomics results, we hypothesised that the RAS-MAPK pathway stands out as the most critical effector for mediating oncogenic METΔex14 activity. To investigate this, we established an isogenic *MET* overexpression model system to directly compare the effects of wild type and mutant MET signaling on its downstream effectors. Using a doxycycline-inducible vector, we generated HEK293T cell lines expressing wild type *MET*, its oncogenic mutant isoform *METΔex14*, or GFP. Additionally, we further included a *MET-Y1003F* mutant isoform to investigate whether loss of the c-Cbl binding site encoded by exon 14 phenocopied loss of the exon in its entirety. We found that, despite similar levels of total MET protein across all three established cell lines after the addition of doxycycline, the *METΔex14*-expressing cells exhibited a notable increase in phosphorylation of its kinase domain (Y1234). By comparison, phosphorylation of this residue in both the wild type and Y1003F mutant MET receptors was significantly lower (Figure 3A). Downstream of MET, we observed an increase in the level of active, GTP-bound RAS within the HEK293T cells, compared to the more modest increases observed in cells overexpressing wild type *MET* or the *MET Y1003F* mutant (Figure 3B). In agreement with this observation, phosphorylation levels of downstream RAS effectors MEK1/2 and ERK1/2 were higher, indicating increased activation of the RAS-MAPK pathway as a whole (Figure 3C). As MET is capable of recruiting and phosphorylating additional effectors, such as PI3K and STAT3, we assessed by Western blotting their relative degree of activating phosphorylation across our isogenic cell lines. We found that phosphorylation of these second messengers or their direct downstream targets did not vary between MET receptor status (Figure 3A), thereby suggesting that the mutant MET receptor is preferentially activating the RAS-MAPK pathway, rather than indiscriminately upregulating downstream signaling pathways due to increased receptor phosphorylation. In agreement with the findings from our isogenic system, we also observed greater ERK1/2 phosphorylation in MET-addicted cancer cells expressing *METΔex14* (Hs746T/H596) vs. wildtype *MET*-amplified (H1993) cells (Figure 1A). Interestingly, ERK1/2 phosphorylation was largely lost in response to MET TKI treatment across both H1993 and Hs746T cells. However, only in H1993 cells were pERK1/2 levels able to rebound despite sustained MET inhibition (Supplementary Figure S2). This suggests that, while MET plays an active role in MAPK signaling in MET WT-addicted cells, it is not necessary for ERK1/2 phosphorylation. In contrast, Hs746T cells, which fail to show a rebound in pERK1/2 levels, appear to be wholly dependent on METex14 activity for MAPK signaling.

Though ERK1/2 initially undergoes phosphorylation via the MAPK cascade in the cytoplasm, its nuclear translocation is required for the induction of proliferation via phosphorylation of key transcription factors [47]. Thus, we sought to determine whether increased overall ERK1/2 phosphorylation via METΔex14 stimulation might lead to increased rates of nuclear importation, and subsequently, stronger growth effects. We first compared nuclear levels of pERK1/2 in our isogenic lines via fractionation of nuclear and cytoplasmic cell compartments with and without HGF treatment. Isogenic doxycycline-treated lung epithelial (HPL1D) cells overexpressing the MET WT or MET mutant receptor did not exhibit increased ERK phosphorylation levels overall. However, upon treatment with HGF, we observed a marked increase in both cytosolic and nuclear pERK1/2 levels, specifically in the *METΔex14*-expressing cells (Figure 3D, Supplementary Figure S5). Notably, *METΔex14*-expressing cells with greater levels of nuclear pERK1/2 also showed elevated levels of ETV5, cFOS, and FRA1 phosphorylation (Figure 3E). All three transcription factors are known nuclear targets of ERK1/2, and play key roles in promoting proliferation and maintenance of alveolar type II cells, which are the precursor for LUAD. These findings point to the possibility that the *METΔex14* mutation enhances the ability of the MET receptor to drive growth through the RAS-MAPK pathway. Despite our above findings, however, overexpression of neither the wild type nor mutant receptors elicited an increase in proliferation in HEK293T cells (Supplementary Figure S3). However, a small but noticeable increase conferred by the METΔex14 receptor was observed in HPL1D lung epithelial lines under anchorage-independent conditions, an effect that was lost upon treatment with ERK1/2 inhibitor SCH772984 (Supplementary Figure S4).

### 3.4. MET-Driven Cell Growth Is Dependent on RAS/MAPK Pathway Activity

**Figure 4:**
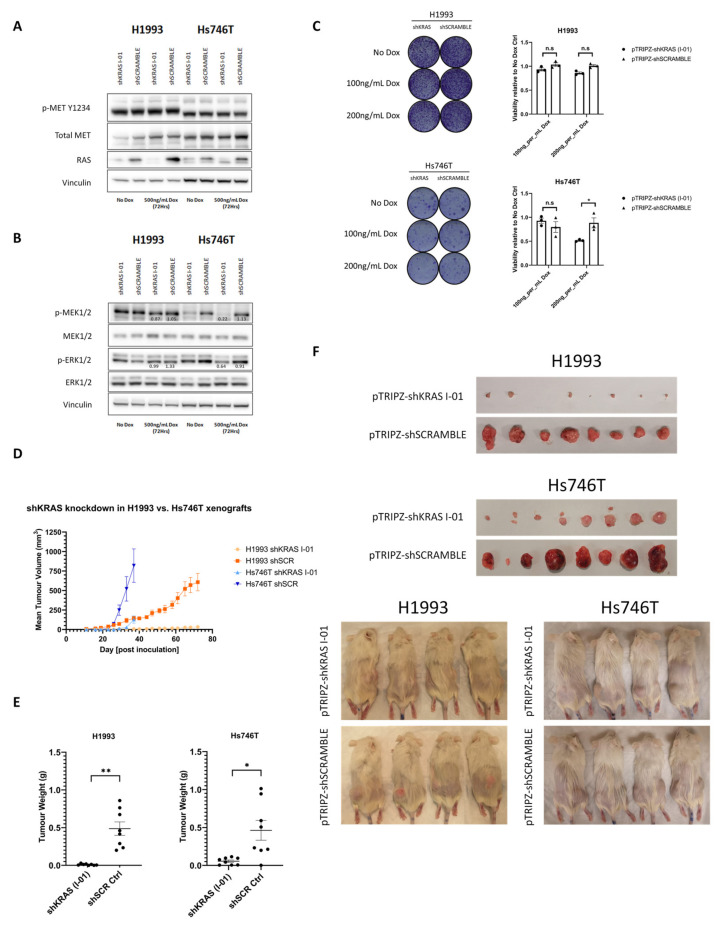
MET-driven cell growth is dependent on activation of the Ras/MAPK pathway. (**A**) Western blot analysis depicting partial or total loss of Ras protein in H1993 and Hs746T carcinoma cells following doxycycline treatment (500 ng/mL Dox for 72 h)**.** (**B**) Western blot analysis showing the effect of KRAS knockdown on the phosphorylation of downstream effectors MEK1/2 and ERK1/2. (**C**) Viability assay by 6-well clonogenics demonstrating a negative impact of KRAS loss on cell viability in *METΔex14*-expressing Hs746T cells compared to *MET* WT-expressing H1993 cells in vitro, as measured by Alamar Blue and Crystal Violet staining. (**D**) Tumour growth kinetics at endpoint of H1993 (shKRAS: *n* = 8; shSCR: *n* = 8) and Hs746T (shKRAS: *n* = 8; shSCR: *n* = 8) cells injected subcutaneously into flanks of NRG mice maintained on doxycycline diet (starting 4 days before injection) demonstrate that loss of KRAS hinders tumour growth in vivo in both MET-addicted cell lines. (**E**) Tumour weights at endpoint correlate with our results in (**D**), demonstrating that loss of KRAS hinders tumour growth in vivo in both cell lines. (**F**) Visual comparison of H1993 vs. Hs746T xenograft tumours with or without KRAS knockdown. n.s, not significant; * *p* < 0.05; ** *p* < 0.01 for the indicated comparisons.

The propensity of the METΔex14 receptor to specifically hyperactivate RAS-MAPK signaling led us to evaluate whether METΔex14-addicted cancer cells are more reliant on this pathway for survival. To address this, we transfected pInducer20 constructs expressing KRAS-targeting shRNA (shKRAS I-01) or non-targeting control (shSCRAMBLE) into Hs746T and H1993 cells, enabling us to conditionally knock down KRAS in these cells upon the addition of doxycycline. Induction of shKRAS with doxycycline led to suppression of total RAS protein in H1993 cells (Figure 4A). However, multiple RAS proteins were detected in Hs746T cells, of which only one was successfully suppressed by shKRAS induction, suggesting additional members of the RAS gene family may play a role in these cancers besides KRAS. Nevertheless, shRNA-mediated KRAS knockdown led to complete and partial loss of MEK1/2 and ERK1/2 phosphorylation, respectively, in *METΔex14*-expressing Hs746T cells (Figure 4B). In contrast, KRAS knockdown in H1993 cells did not lead to a discernible decrease in MEK1/2 and ERK1/2 phosphorylation, suggesting that KRAS is not necessary for the maintenance of MAPK pathway activity in the context of wild type MET signaling. Interestingly, we found KRAS knockdown to have a significant detrimental effect on METΔex14-addicted Hs746T cells resulting in reduced cell proliferation and colony formation in vitro while growth of H1993 cells remained unaffected (Figure 4C). We attempted to validate our findings in vivo via subcutaneous injection of shKRAS- or shSCR-expressing H1993 and Hs746T cells. In contrast to our in vitro observations, we found that doxycycline-induced expression of shKRAS significantly impaired the growth of subcutaneous xenografts for both H1993 and Hs746T cells (Figure 4D–F), suggesting that the integrity of KRAS-mediated signaling activity remains a vital component of MET-driven oncogenesis.

### 3.5. MET-Independent KRAS Activation Is Sufficient to Rescue Cell Death following MET TKI Treatment in METΔEx14-Addicted Cells

**Figure 5:**
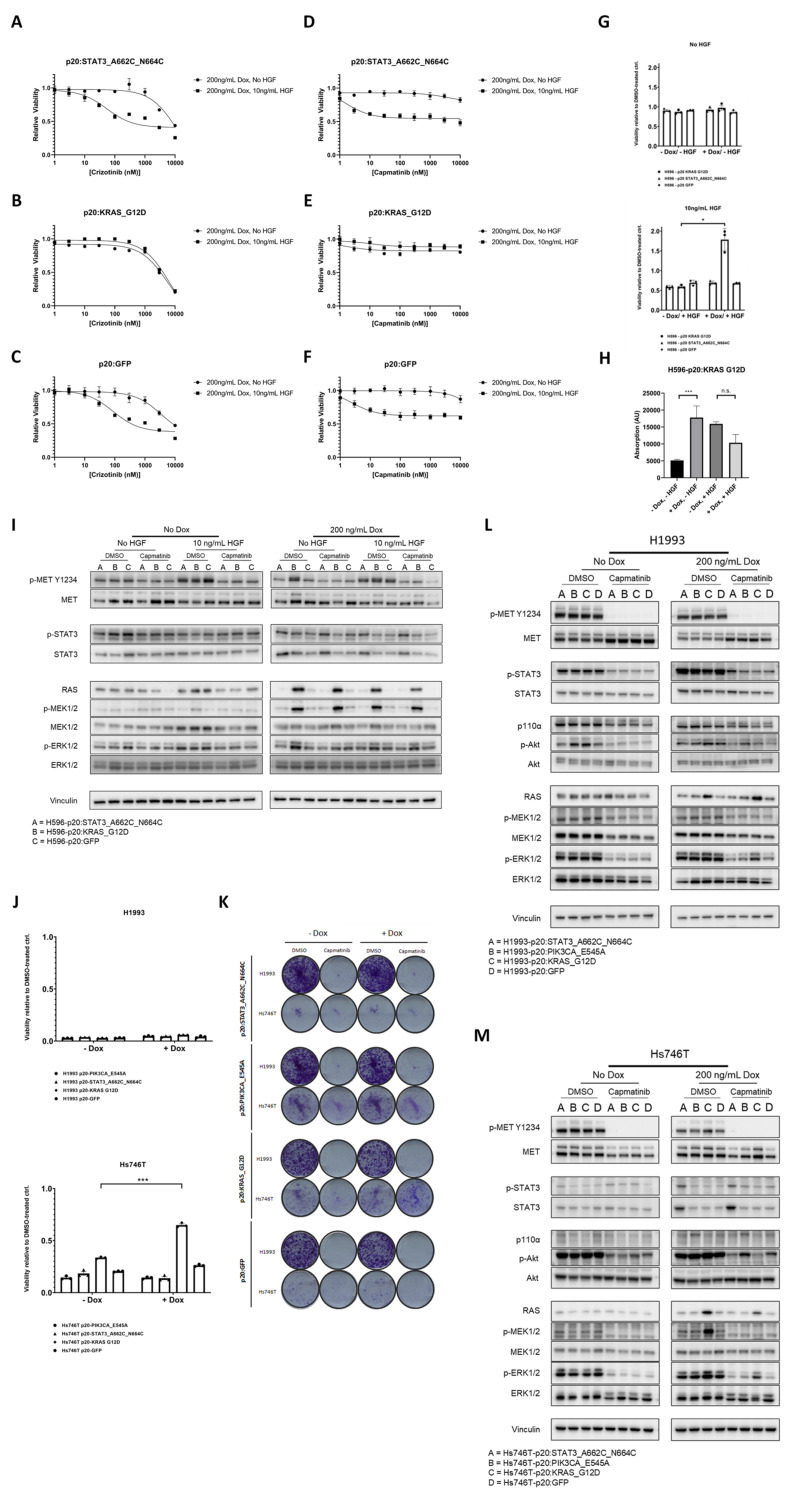
Decoupling MET from the Ras/MAPK pathway is sufficient to rescue cell death following MET TKI treatment in METΔEx14-addicted cells. (**A**–**F**) Dose response curves of H596 lung adenosquamous cells to Crizotinib and Capmatinib, depicting loss of HGF-dependent sensitivity to MET tyrosine kinase inhibitors in response to KRAS^G12D^ expression. (**G**) Cell viability assays of H596 cells conditionally expressing oncogenic *STAT3*, *KRAS*, or *GFP* in the presence (top) or absence (bottom) of HGF. Values shown are calculated from Alamar Blue absorbance readings of cells grown in MET TKI (Capmatinib, 100 nM) relative to those grown in 0.1% DMSO. (**H**) Growth assays of H596 cells cultured under indicated conditions to compare the impact of oncogenic *STAT3*, *KRAS*, or *GFP* expression on H596 cell growth in the presence or absence of HGF-driven proliferation. (**I**) Western blot analysis of MET effector proteins overexpressed by the pInducer20 promoter in the presence of doxycycline (200 ng/mL), along with their downstream signaling targets with or without HGF (10 ng/mL) or Capmatinib (100 nM) in H596 cells. (**J**,**K**) Viability assay in 6-well plates, as measured by (**J**) Alamar Blue and (**K**) Crystal Violet staining, demonstrating mutant KRAS-mediated rescue cell viability from MET TKI treatment in Hs746T, but not H1993, cells. (**L**,**M**) Western blot analysis of MET effector proteins overexpressed by the pInducer20 promoter in the presence of doxycycline (200 ng/mL), along with their downstream signaling targets with or without Capmatinib (100 ng/mL), in (**L**) H1993 and (**M**) Hs746T cells. n.s, not significant; * *p* < 0.05; *** *p* < 0.001 for the indicated comparisons.

Our results above suggested that RAS-MAPK signaling remained an important effector pathway for growth and survival in the context of MET-driven oncogenesis in general. However, recent clinical screens found that amplification and/or activating mutations in the gene encoding *KRAS* decoupled the RAS-MAPK pathway activity from the MET receptor, constituting the most common form of resistance against MET-targeted therapies in *METΔex14*-presenting tumours [23,24,25]. Thus, we wondered whether activation of this pathway was sufficient to account for the driving force behind the tumourigenic potential of METΔex14-addicted cells specifically. To investigate this hypothesis, we generated cancer lines conditionally expressing oncogenic forms of MET effectors KRAS, PI3K, and STAT3 (p20-*KRAS^G12D^*, p20-*PIK3CA^E545A^*, and p20-*STAT3^A662C_N664C^*). In H596 lung adenosquamous cells, which exhibit HGF-dependent growth reversible via MET inhibition, we found that doxycycline-induced expression of constitutively active mutant KRAS led to HGF-independent cell growth (Figure 5H) as well as loss of sensitivity to MET inhibition (Figure 5B,E,G). Doxycycline-induced overexpression of a parallel MET effector, STAT3 (Figure 5A,D,G), by contrast failed to elicit any rescue, similar to GFP-expressing controls (Figure 5C,F,G). While these results point to KRAS as a key mediator of MET-driven growth, a key caveat remains in that these cells do not normally exhibit MET-addicted behaviour despite expressing an oncogenic form of MET receptor. As mentioned earlier, this is likely due to a coexisting *PIK3CA^E545A^* oncogenic mutant allele. Therefore, while constitutive KRAS activity is sufficient to decouple HGF-driven MET activity from cell growth in H596 cells, it is not possible to assess the importance of PI3K activity in facilitating this decoupling. To address this, we repeated our observations in MET-addicted H1993 and Hs746T lung adenocarcinoma and gastric carcinoma cells, respectively. Similar to our observation in H596 cells, overexpression of *KRAS^G12D^* partially rescued MAPK phosphorylation (Figure 5M) and decoupled cell growth and survival from MET activity in *METΔex14*-expressing Hs746T cells (Figure 5J, bottom panel, Figure 5K). Notably, expression of the same mutant *KRAS* oncogene, despite similarly restoring MAPK phosphorylation from MET TKI treatment (Figure 5L), failed to rescue viability and growth in H1993 cells, which in contrast to Hs746T cells, express and are addicted to the wild type MET receptor (Figure 5J, top panel, Figure 5K). Finally, we observed that overexpression of oncogenic *PIK3CA^E545A^*, similar to *STAT3^A662C_N664C^*, did not rescue either line from MET TKI treatment (Figure 5I). As a whole, our results point to KRAS being the crucial signaling effector that is responsible for facilitating MET-driven growth in METΔexon14-addicted cells, while also playing a role in maintaining growth and tumourigenesis in MET WT-addicted cancer cells as well.

## 4. Discussion

The present study is aimed at facilitating the design of rational combinatorial therapies against METΔexon14-driven cancers, where response rates from single-treatment targeted therapies against MET alone remain largely mixed [21,48,49,50]. The response rates to capmatinib and tepotinib, two FDA-approved MET inhibitors, are below that seen with EGFR and RET inhibitors [51,52]. Here, we provide preclinical evidence pointing to the importance of KRAS as a critical mediator of METΔexon14-driven LUAD. Crucially, this observation was made by comparing the transcriptional profiles of MET-addicted cancers expressing either *METΔexon14* or exhibiting high-grade amplification of the wild type *MET* gene, suggesting divergent mechanisms underpinning MET-driven tumourigenesis. This provides some justification for stratifying patients in the clinic based on *MET* alteration status when designing combined drug therapies, as one may expect differing mechanisms of primary or secondary resistance to first-line MET targeted therapy based on the type of alteration present. This is especially important given the low ORRs in patients with either alteration when treated with MET TKi [53]. Already, clinical trials testing the efficacy of MET inhibitors in *METΔexon14*-presenting tumours have identified amplification and/or activating mutations in the *KRAS* or *BRAF* genes (as well as activating mutations in the PI3K-AKT pathway) to be a common form of acquired resistance [23,24,25,54,55]. This observation is consistent with our findings that RAS is the primary mediator of METΔexon14-driven tumourigenesis, whereupon decoupling this effector from MET activity via mutant KRAS leads to MET TKi resistance in *METΔexon14*-mutant, but not *MET*-amplified, cells. By contrast, in vitro studies have identified alterations in *MYC*, *mTOR*, and *PIK3CA*, but notably rarely *KRAS* alterations, as potential drivers of resistance in MET-addicted cancer cells with MET-amp [56,57,58].

The ERK1/2 serine-threonine protein kinases sit at the end of a multi-tiered phosphorylation cascade, beginning with KRAS-mediated activation of RAF-1 (MAP3K) and ending with their phosphorylation and nuclear translocation [59]. This latter step is an important one, as it is required for ERK1/2 to promote ETS/AP1-mediated transcriptional regulation of cell proliferation and motility [47,59]. Interestingly, while ectopic expression of *MET WT* and *METΔexon14* in human lung HPL1D cells led to similar levels of ERK1/2 phosphorylation (as opposed to higher ERK phosphorylation from ectopic METΔexon14 expression in HEK293T cells, which have relatively little background MAPK pathway activity), we nevertheless observed a markedly higher degree of pERK1/2 in the nuclear fractions of both cell lines. Consistent with these results, we also found higher levels of phosphorylated cFOS and FRA1, which together with FRA2 and c-JUN form the heterodimeric complex of AP1. Additionally, protein levels of ETV5, a key player in the maintenance of Ras-induced lung adenocarcinoma in alveolar type II cells [60], were higher in HEK293T cells expressing *METΔexon14* compared to just ectopic *MET* WT overexpression. The oncogenic nature of *MET* exon14-skipping mutations would provide the most direct reason why *METΔexon14* expression promotes greater increase in RAS-MAPK signaling compared to amplification of its wild type counterpart. However, high-grade amplification of wild type *MET* is also considered a driver alteration, such as in H1993 cells, and can be treated successfully in the clinic using anti-MET targeted therapies [61,62,63]. Furthermore, increased MET kinase domain phosphorylation in the mutant receptor appeared to lead to increased ERK1/2 phosphorylation without a concomitant increase in AKT or STAT3 phosphorylation. This suggests that, compared to the wild type receptor, the METΔexon14 mutant receptor has a specific predilection for activating the RAS-MAPK pathway.

One possible reason may be due to a critical importance of the juxtamembrane (JM) domain in regulating the balance between receptor recycling and degradation pathways. Rapid receptor internalisation following activation is a key mechanism by which cells rapidly switch off signaling to prevent sustained receptor activity, hence the oncogenic effects of losing this domain [64]. However, MET is known to continue signaling within its endosomal compartments following endocytosis, with some evidence suggesting that this compartmentalisation of internalised receptors plays an important role in spatially modulating signal transduction processes within the cell [65,66]. For instance, loss of the recycling adaptor GGA3 has been shown to enhance MET trafficking into degradative compartments, leading to attenuation of ERK1/2, but not AKT, phosphorylation following HGF stimulation [67,68]. Additionally, the authors showed that pERK1/2 attenuation as a result of defective MET recycling significantly impaired cell motility and migration, a process we found to be significantly enriched for in our GO analysis of GVSA leading edge genes upregulated in *METΔexon14*-expressing cell lines in response to HGF.

In addition to spatiotemporal regulation of MET signaling, its pleiotropic behaviour is tightly controlled through a plethora of binding partners, including adaptor proteins GRB2, CRK, and SHC, as well as the scaffolding protein GAB1 [13]. Site-specific recruitment of these proteins to the MET docking domain confers specificity to MET downstream signaling [69]. In particular, GRB2 binding is a necessary precursor for RAS activation through the recruitment of guanine nucleotide exchange factor SOS1 [70]. This MET-GRB2 interaction is an important mediator of MET-driven oncogenic behaviour, capable of predicting response to MET targeted therapy [71]. Changes to receptor structure that have the potential to introduce steric alterations may affect recruitment of binding partners, and of GRB2 especially if its reserved MET Y1356 binding site is affected [69]. X-ray crystallography experiments, similar to those performed on other MET mutants in the past, may reveal whether structural changes in the METΔexon14 receptor, if any, play a causative role in driving its oncogenic behaviour [72]. Finally, future studies incorporating the use of phosphoproteomics would help quantify the dynamics of METΔexon14 activation and downstream signaling networks; such techniques have been used to study the EGF receptor in the past, and can be easily deployed to characterise METΔexon14 signaling in the future [73,74].

To our knowledge, there have been no studies examining the direct molecular consequences of losing the JM domain in its entirety, though the MET Y1003 phosphotyrosine site within this domain has been identified to be functionally indispensable for mediating receptor degradation via its recruitment of the E3 ubiquitin ligase c-CBL [68]. On this basis, we were surprised to observe that introducing MET with a mutation at this site failed to phenocopy exon 14 loss in HEK293T cells in terms of downstream signaling and proliferation, despite the clear loss of MET ubiquitination [14]. However, Y1003 point mutations are comparatively rare in the clinic, suggesting that loss of the exon in its entirety confers additional oncogenic advantages beyond the impairment of CBL-induced MET degradation. Possibilities include the potential for structural impacts due to JM loss to affect MET effector recruitment, as well as involvement of other negative regulatory sites in this region, such as the Ser-985 phosphorylation site targeted by PKCδ and ε [75].

## 5. Conclusions

In summary, our report highlights distinct signaling and transcriptional properties elicited by different oncogenic driver alterations affecting MET. In the case of exon 14-skipping mutations, growth and survival advantages are conferred mainly through the RAS-MAPK pathway. This hallmark represents a potential point of weakness exploitable by vertical MET + MEK or MET + ERK inhibition, a strategy that was successfully deployed in EML4-ALK-positive LUADs. By comparison, high-grade amplification of wild-type *MET* appears to be reliant on activation of a more diverse complement of effector signaling pathways to drive tumourigenesis, potentially requiring a more personalised treatment approach. Additional studies comparing molecular mechanisms of MET hyperactivity will be required to fully understand the oncogenic nature of *MET* exon 14-skipping mutations and aid in the design of rational combinatorial therapies to combat drug resistance and improve treatment outcomes.

## Data Availability

Microarray data are uploaded as GEO: GSE198094.

## Supplementary Materials

The following supporting information can be downloaded at: https://www.mdpi.com/article/10.3390/cancers14061378/s1, Figures S1–S5: Supplemental Figures; Table S1: TCGA clinicopathological information; Table S2: Densitometry analyses of all immunoblots; Figure S6: Original immunoblot images.
