# Supplementary material for: MET Exon 14 Splice-Site Mutations Preferentially Activate KRAS Signaling to Drive Tumourigenesis
Source: Cancers (Basel). 2022 Mar 8;14(6):1378. doi: 10.3390/cancers14061378 (PMC8946549; doi:10.3390/cancers14061378)
Supplement: Supplementary file 1 [file cancers-14-01378-s001.zip › Supplementary Figures S1-5.pptx]

## Slide 1
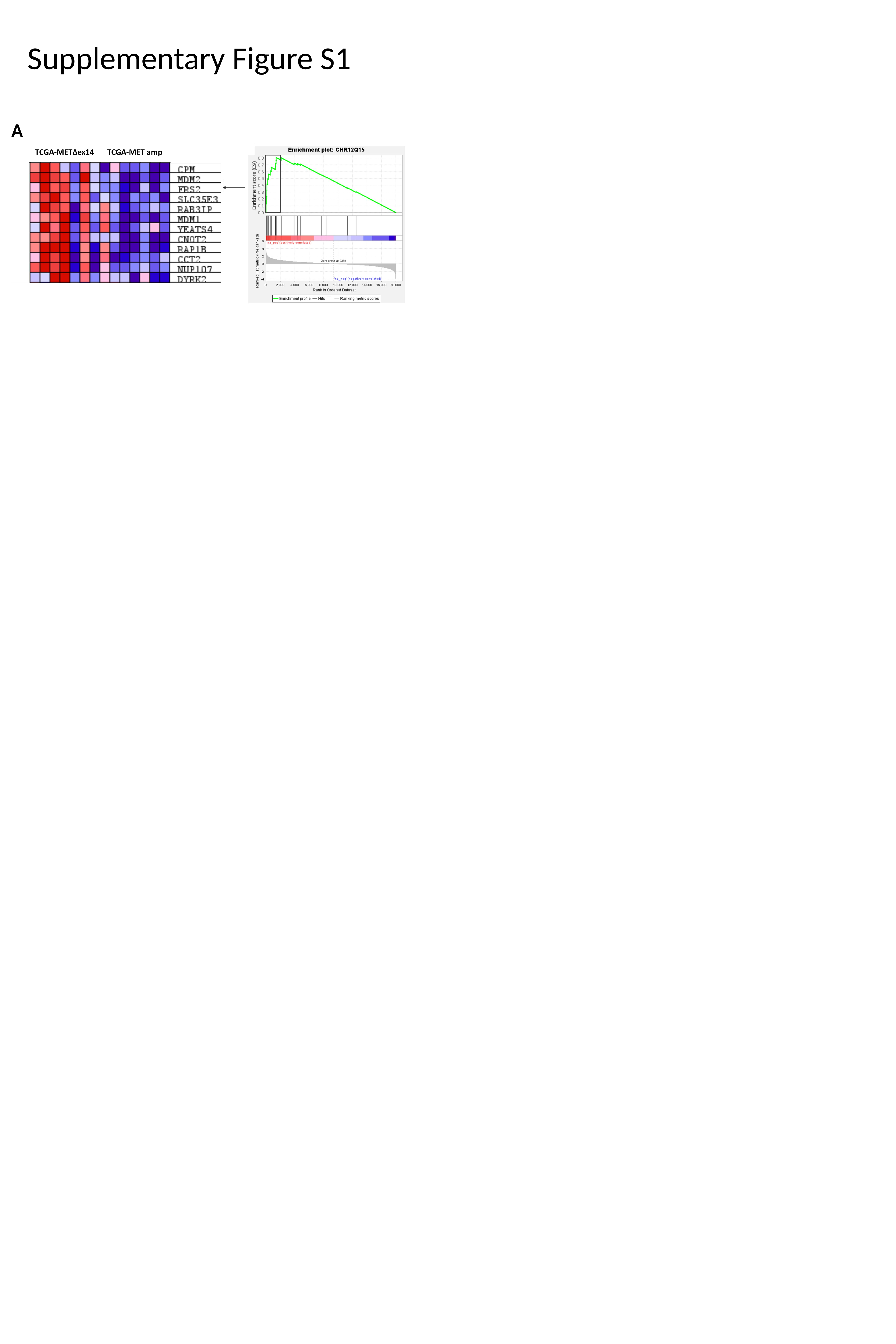

Supplementary Figure S1
 A

## Slide 2
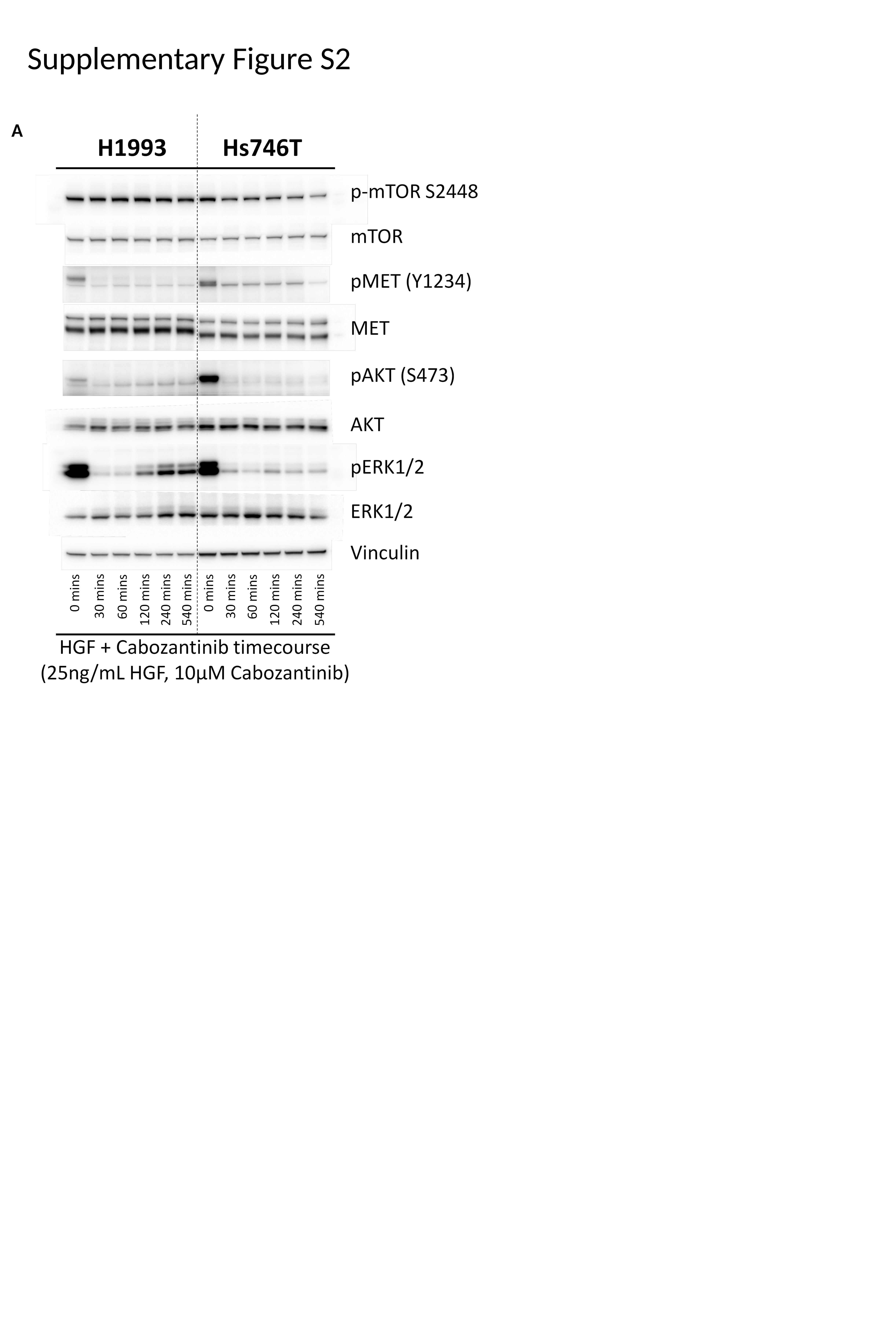

Supplementary Figure S2
 A

## Slide 3
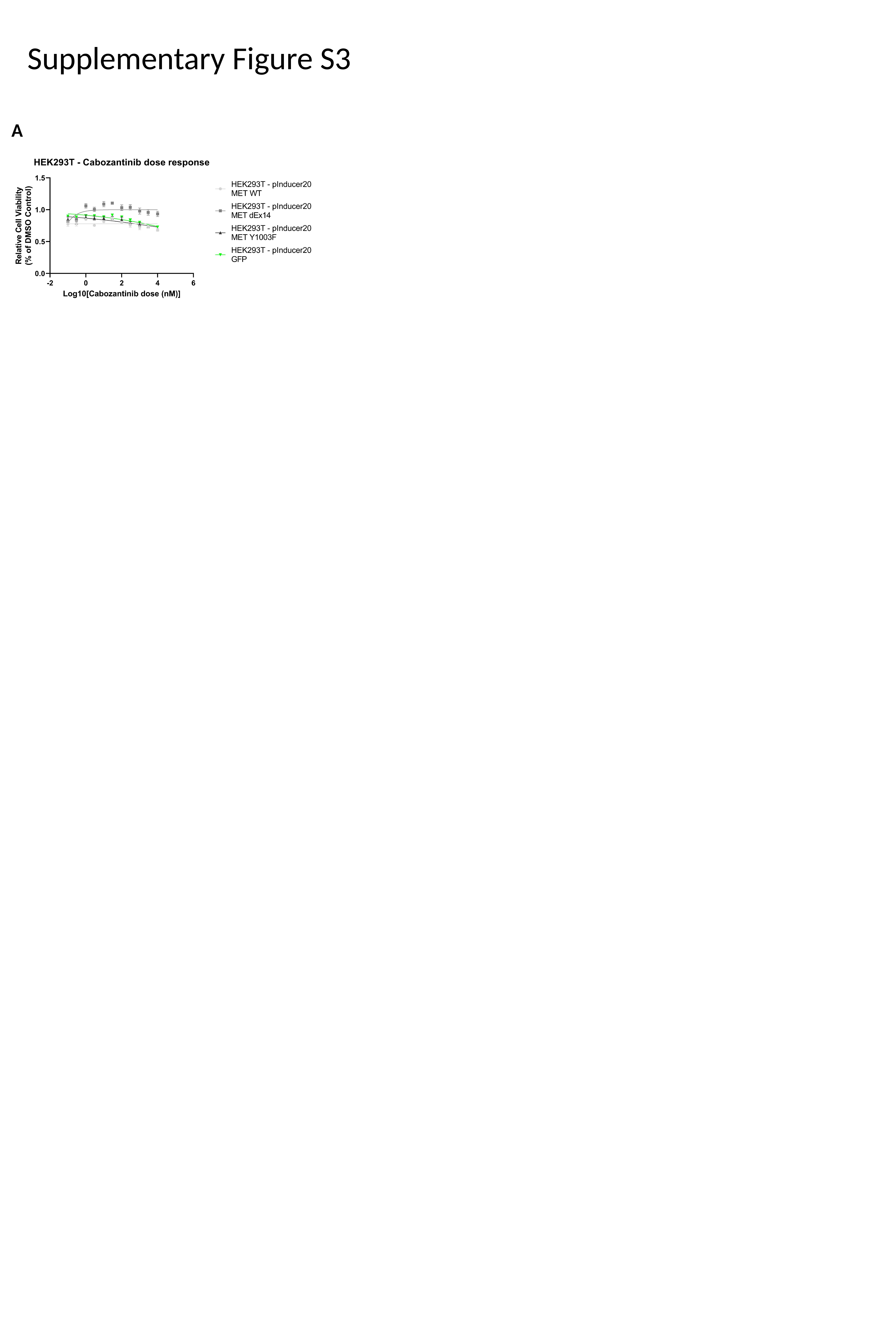

Supplementary Figure S3
 A

## Slide 4
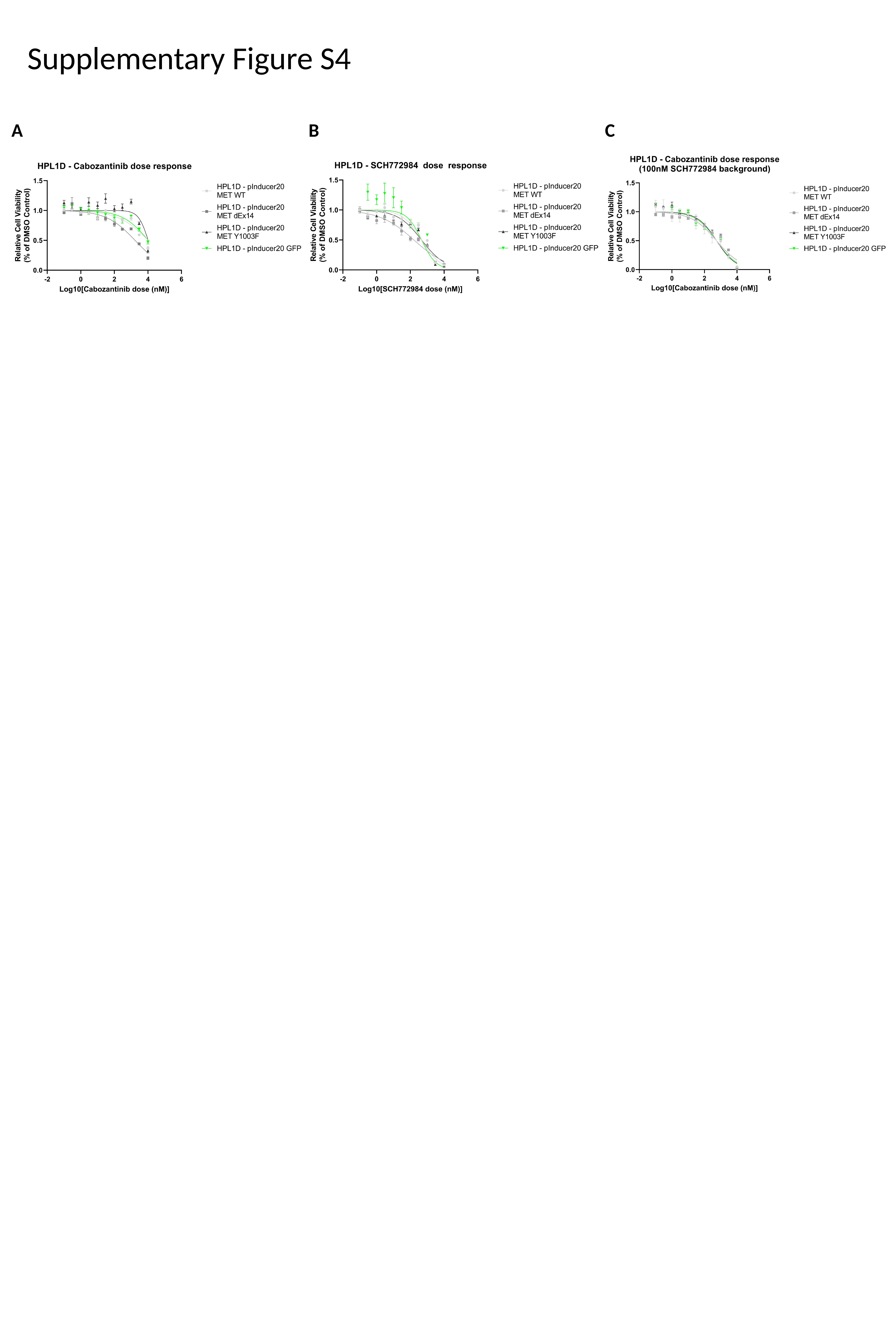

Supplementary Figure S4
 A B C

## Slide 5
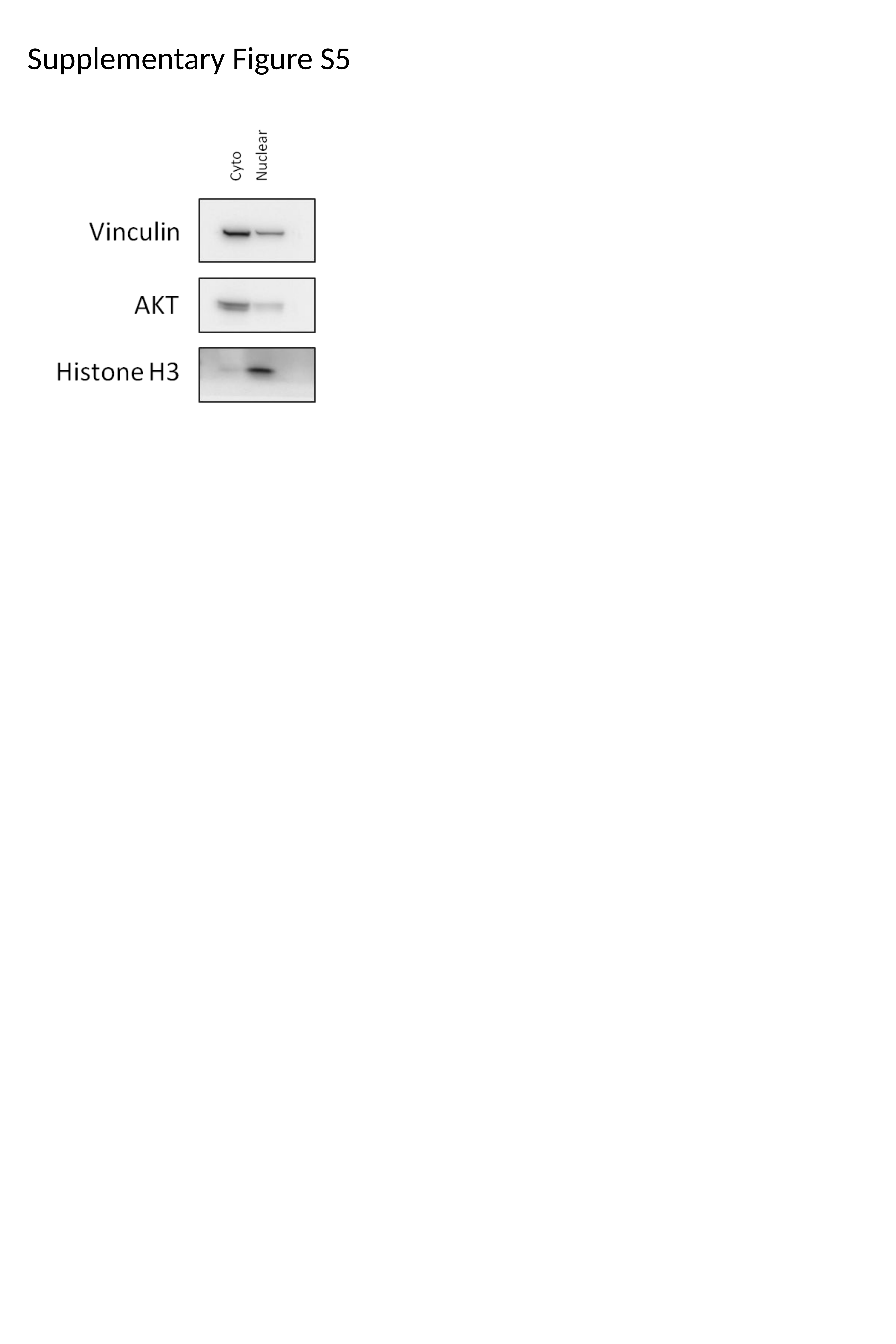

Supplementary Figure S5
