# Supplementary material for: MET Exon 14 Splice-Site Mutations Preferentially Activate KRAS Signaling to Drive Tumourigenesis
Source: Cancers (Basel). 2022 Mar 8;14(6):1378. doi: 10.3390/cancers14061378 (PMC8946549; doi:10.3390/cancers14061378)
Supplement: Supplementary file 1 [file cancers-14-01378-s001.zip › SUPPLEMENTAL INFORMATION.pdf]

SUPPLEMENTAL INFORMATION

Supplementary Tables

Table S1: Accompanying clinicopathological information for RNA-seq data of lung adenocarcinoma samples with *MET*-amplification or *METΔex14* mutation, downloaded from the NIH GDC Data Portal.

Table S2 – Western blot densitometry values for each visible band, quantified using ImageJ software.

Supplementary Figures

Figure S1

(A) GSEA using positional gene sets found most genes in the Chr12Q15 region to be overexpressed in METΔex14 tumour samples, suggesting focal amplification.

Figure S2

(A) WB analysis comparing effects of MET inhibition in MET-addicted H1993 and Hs746T cells shows a complete rebound in ERK1/2 phosphorylation in H1993, but not in Hs746T, cells.

Figure S3

(A) 96-well dose response assay assessing relative sensitivity of HEK293t cells ectopically expressing wild type MET, mutant (Δex14, Y100F) MET, or GFP to Cabozantinib. Both mutant and wild type *MET* overexpression fails to increase sensitivity of HEK293T cells to MET inhibition in HEK293t cells under anchorage independent conditions.

Figure S4

(A) 96-well dose response assays assessing the effects of wild type MET, mutant (Δex14, Y1003F) MET, or GFP overexpression in HPL1D cells on Cabozantinib sensitivity. METΔex14 expression confers a slight increase in sensitivity to both MET (*P*-val = 0.0021) and (B) MAPK inhibition (*P*-val = 0.0045).
(C) The observed increased sensitivity to MET inhibition in METΔex14-expressing cells was lost in the presence of MAPK inhibition (*P*-val = 0.3059), pointing to the MAPK pathway as the chief effector of MET-driven growth.

Figure S5

(A) Side-by-side loading control comparison of nuclear (Histone H3) and cytosolic (Vinculin & AKT) proteins present in either fraction to evaluate the degree of cellular compartment separation.

Figure S6

(A-L) Uncropped, original western blot images for each target included in the figures.
