# Supplementary material for: MET Exon 14 Splice-Site Mutations Preferentially Activate KRAS Signaling to Drive Tumourigenesis
Source: Cancers (Basel). 2022 Mar 8;14(6):1378. doi: 10.3390/cancers14061378 (PMC8946549; doi:10.3390/cancers14061378)

pMET Y1234

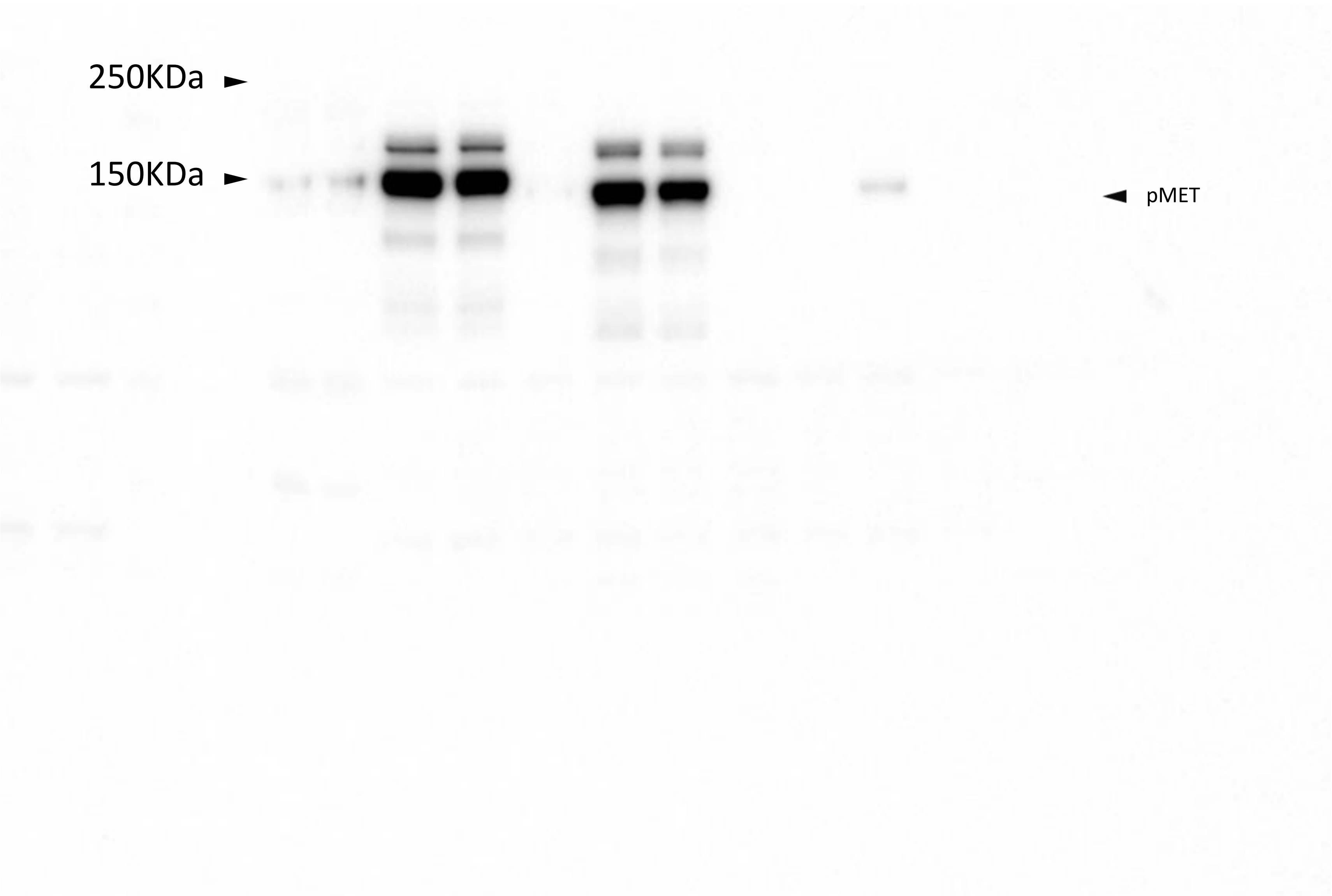

pMET Y1349

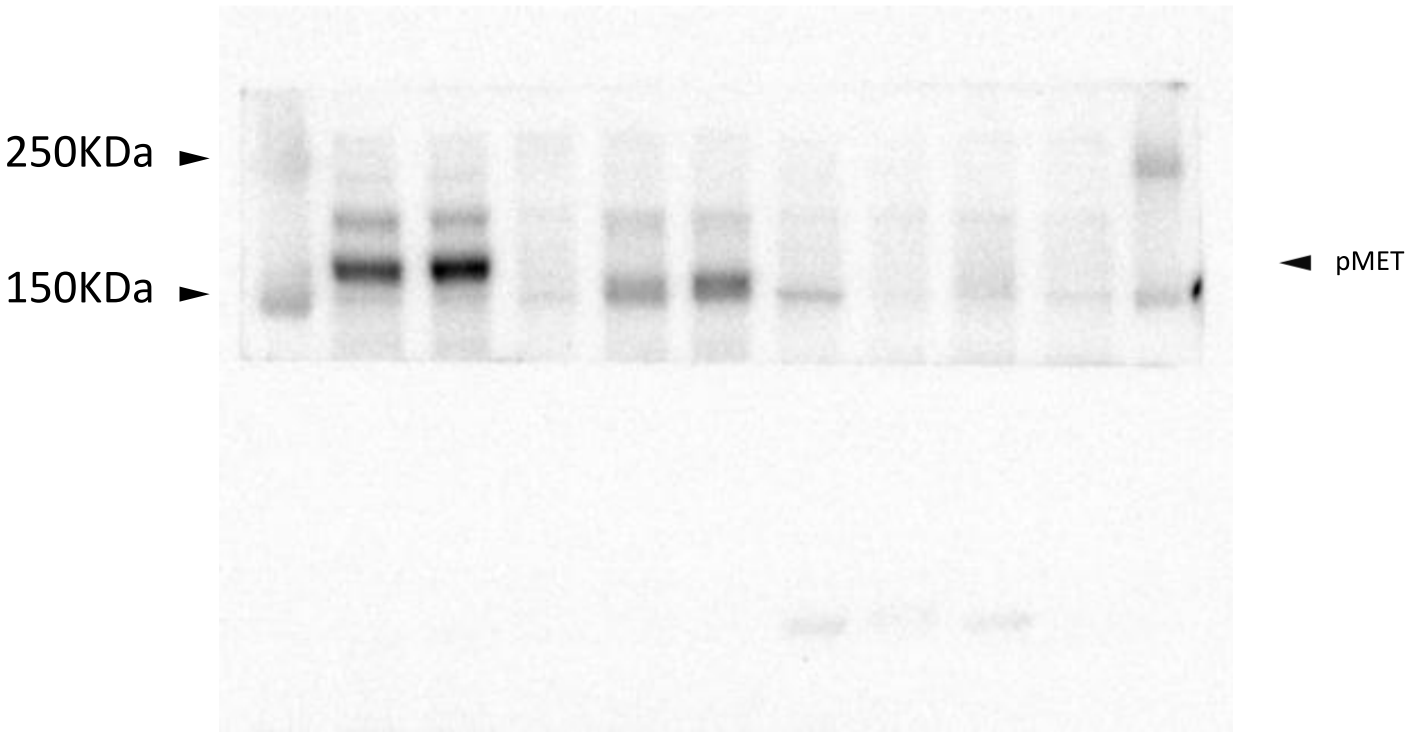

pMET Y1003

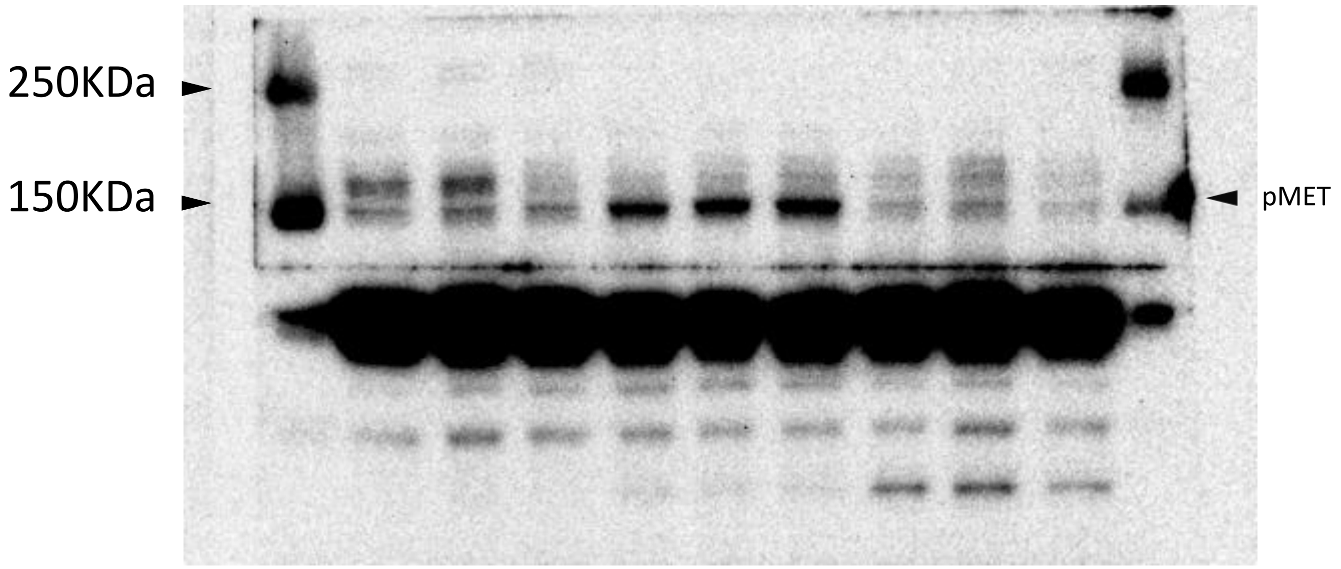

MET

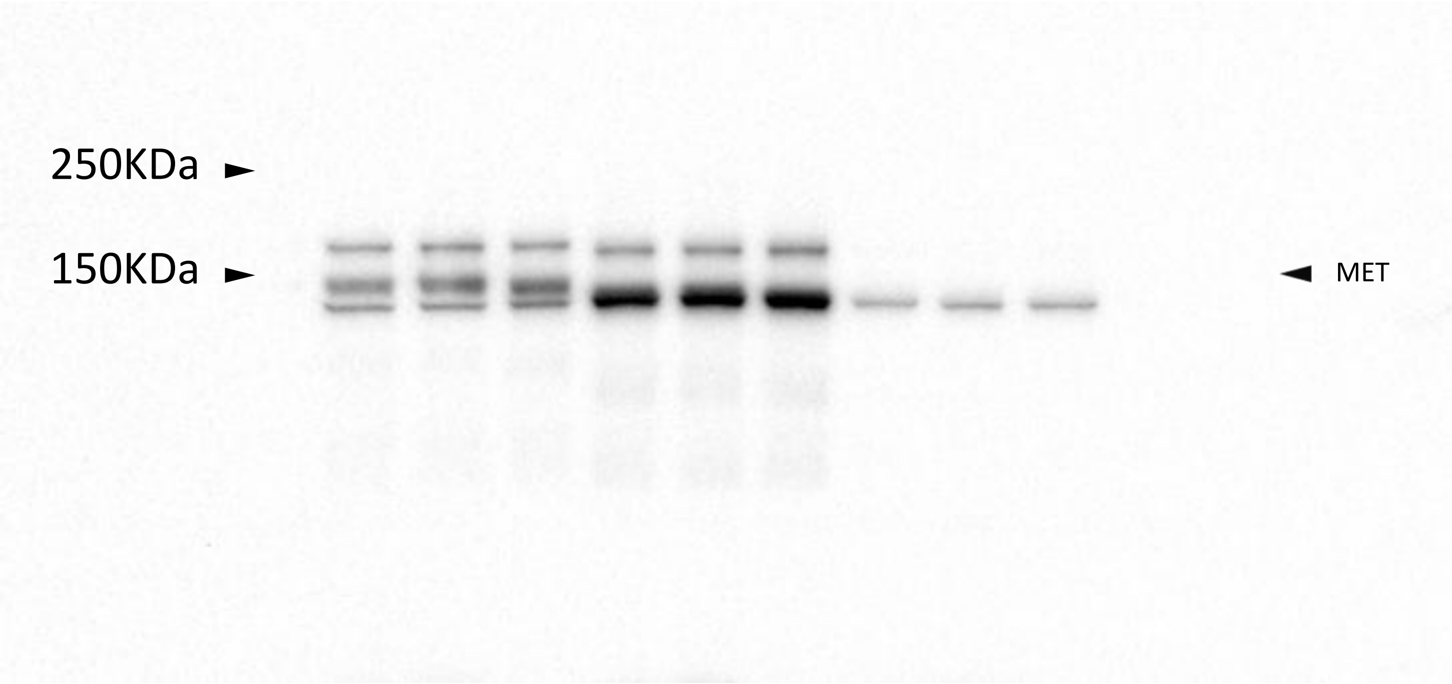

pSTAT3 Y705

150KDa ►
100KDa ►
75KDa ►
50KDa ►

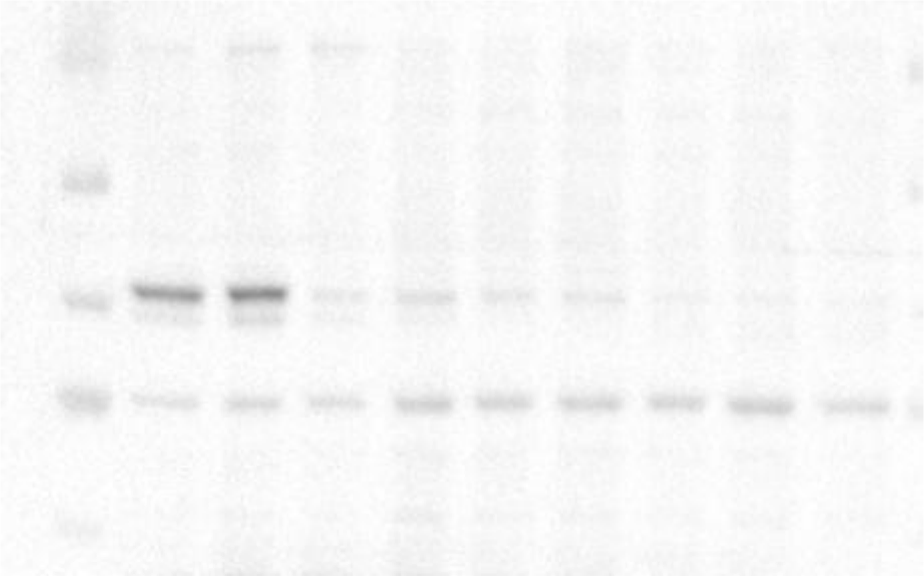

◄ pSTAT3

STAT3

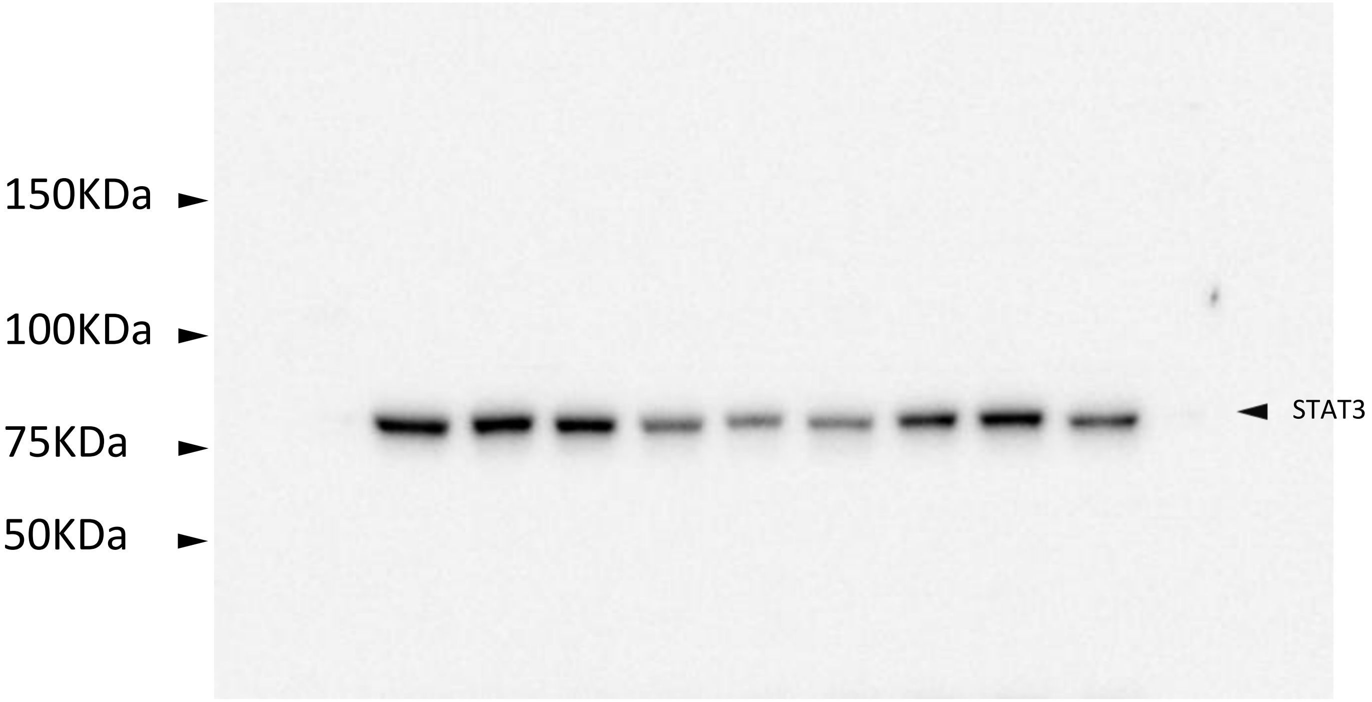

pAKT S473

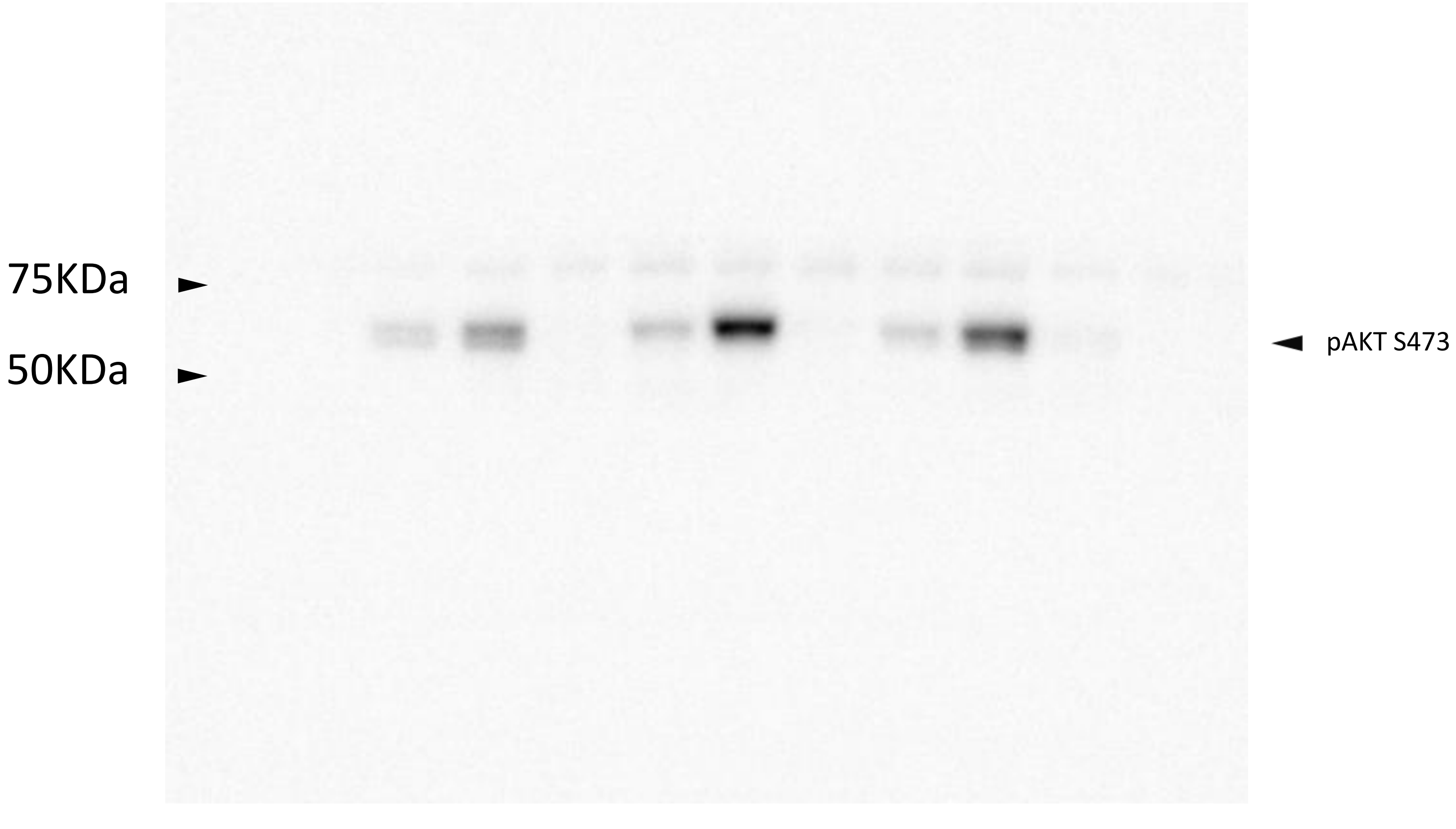

AKT

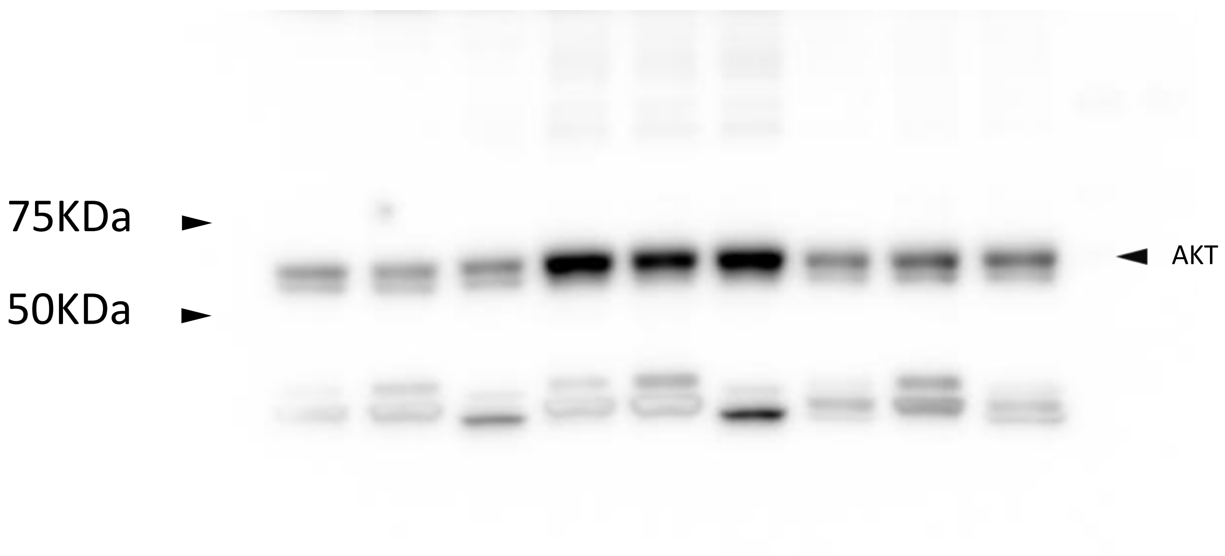

pERK1/2

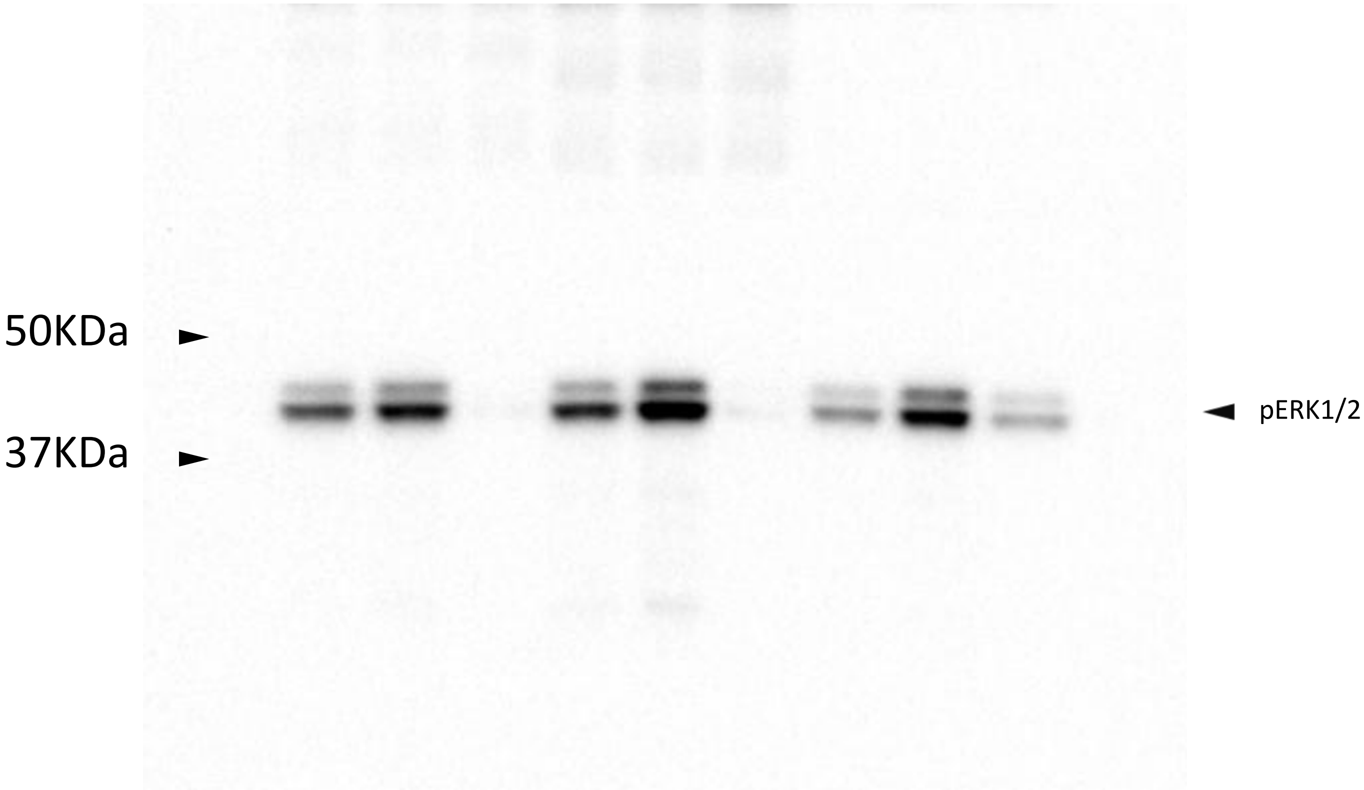

ERK1/2

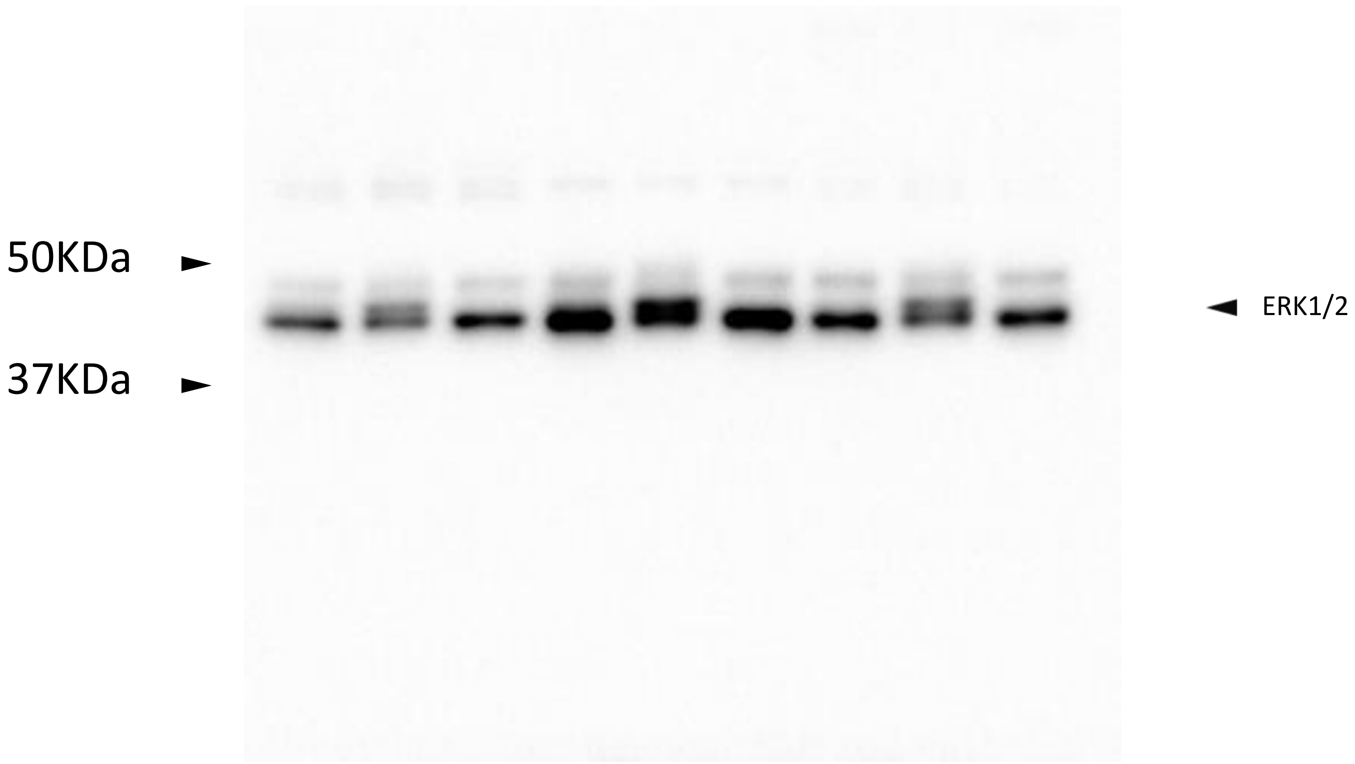

pSRC Y416

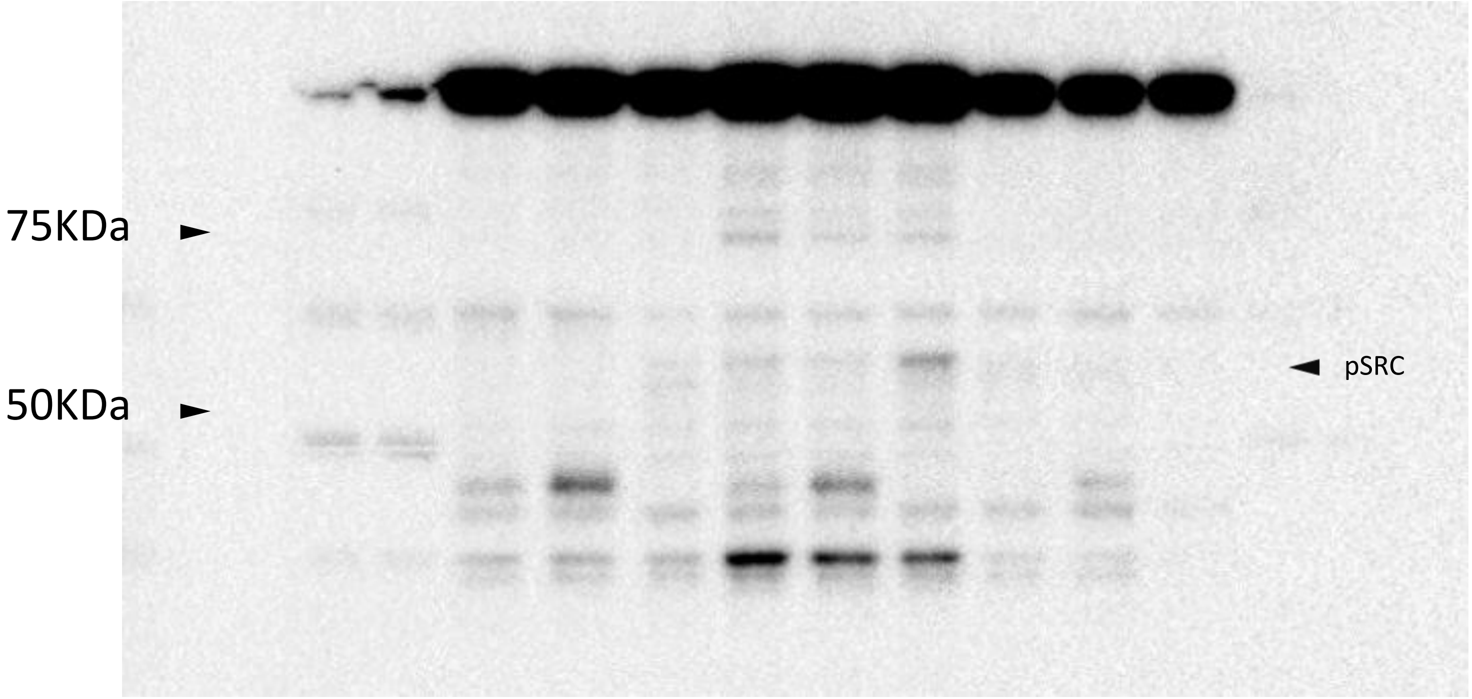

SRC

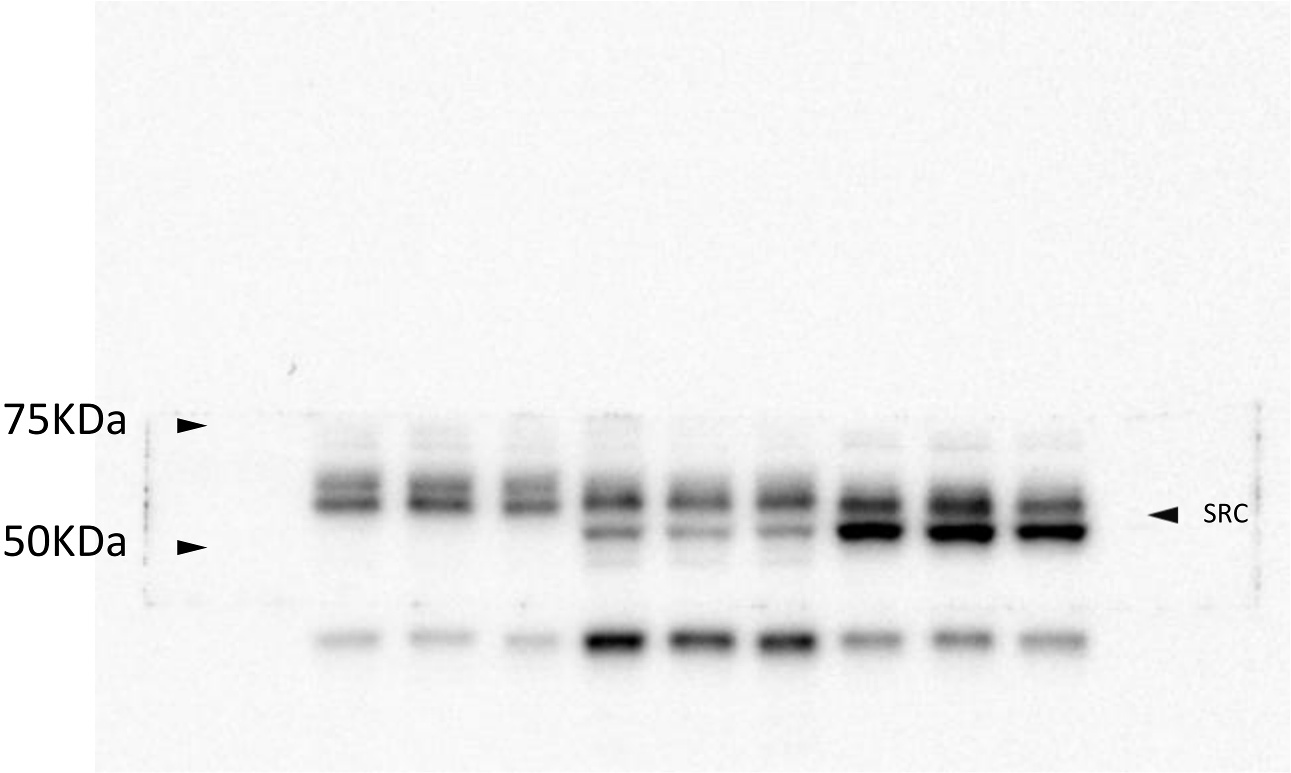

β-Actin

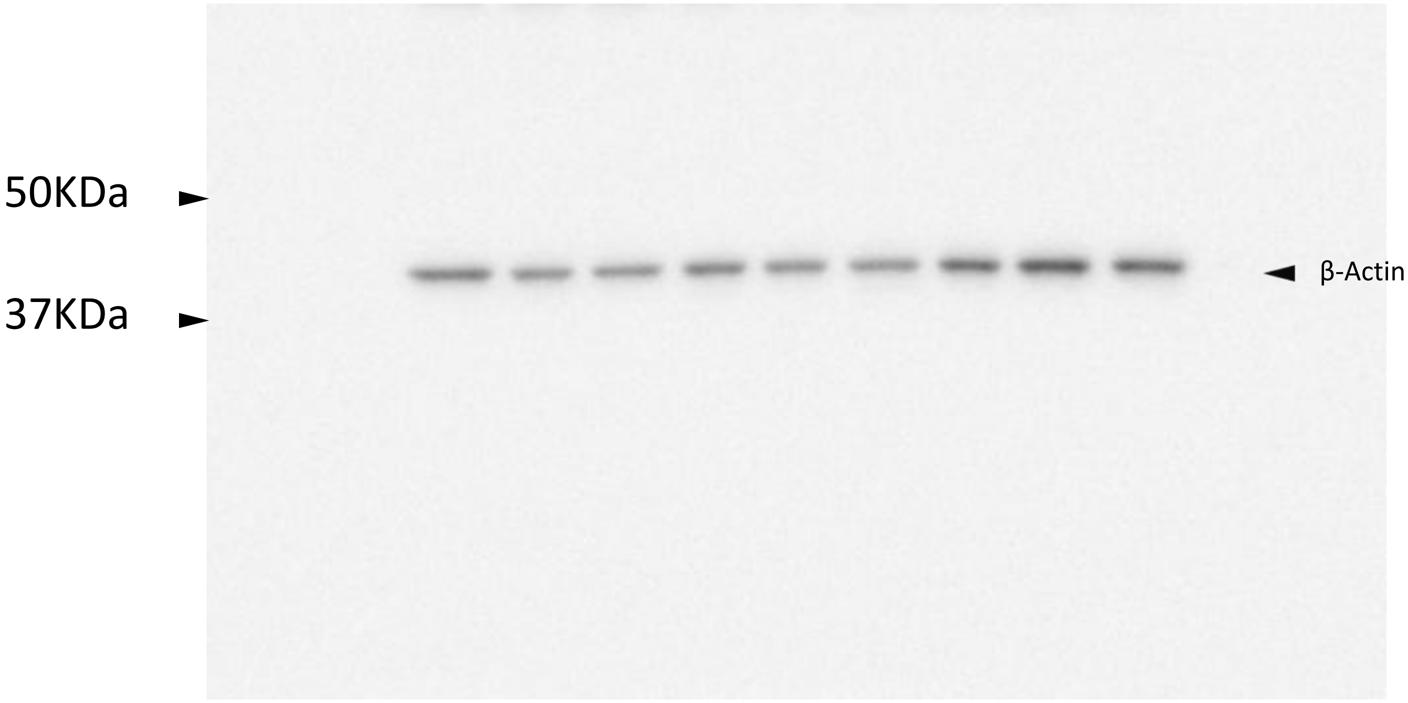

Supplement: Supplementary file 1 [file cancers-14-01378-s001.zip › Supplementary Figure S6 - Original western blots.zip!/Supplementary Figure S6 - Original western blots/(A) Fig 1b.pdf]
