# Supplementary material for: MET Exon 14 Splice-Site Mutations Preferentially Activate KRAS Signaling to Drive Tumourigenesis
Source: Cancers (Basel). 2022 Mar 8;14(6):1378. doi: 10.3390/cancers14061378 (PMC8946549; doi:10.3390/cancers14061378)

RAS

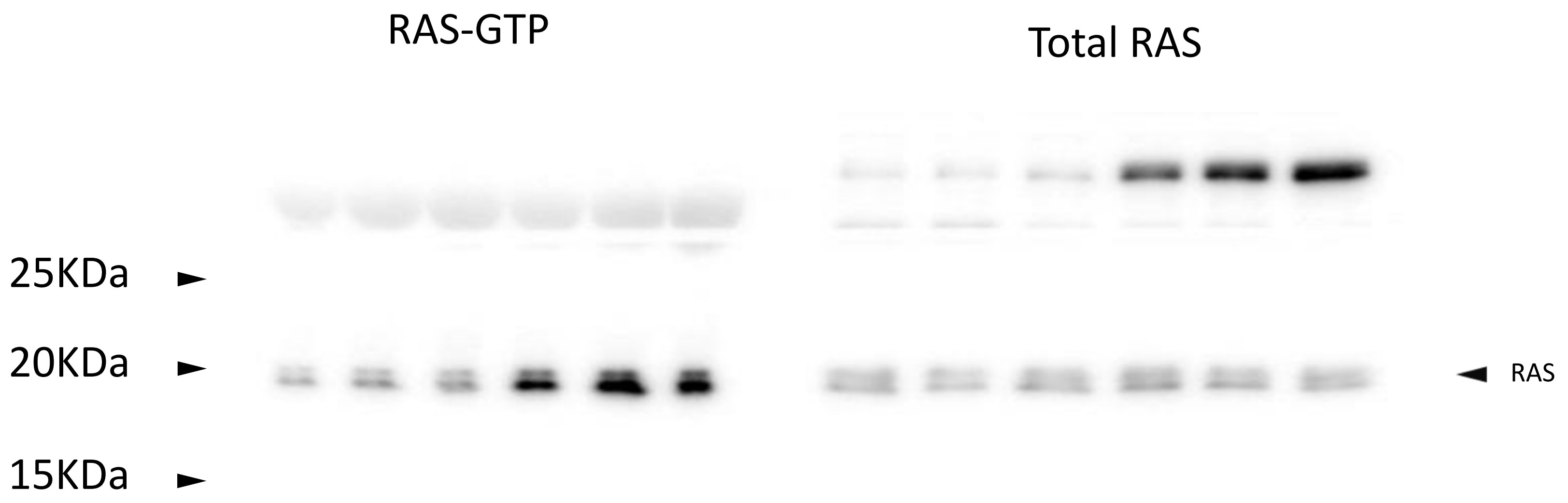

Vinculin

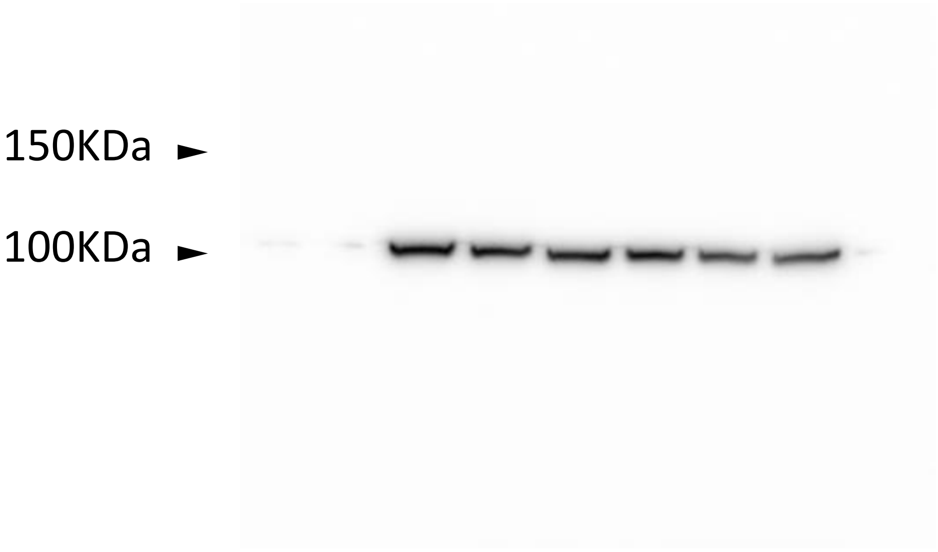

Supplement: Supplementary file 1 [file cancers-14-01378-s001.zip › Supplementary Figure S6 - Original western blots.zip!/Supplementary Figure S6 - Original western blots/(C) Fig 3b.pdf]
