# Supplementary material for: MET Exon 14 Splice-Site Mutations Preferentially Activate KRAS Signaling to Drive Tumourigenesis
Source: Cancers (Basel). 2022 Mar 8;14(6):1378. doi: 10.3390/cancers14061378 (PMC8946549; doi:10.3390/cancers14061378)

MET

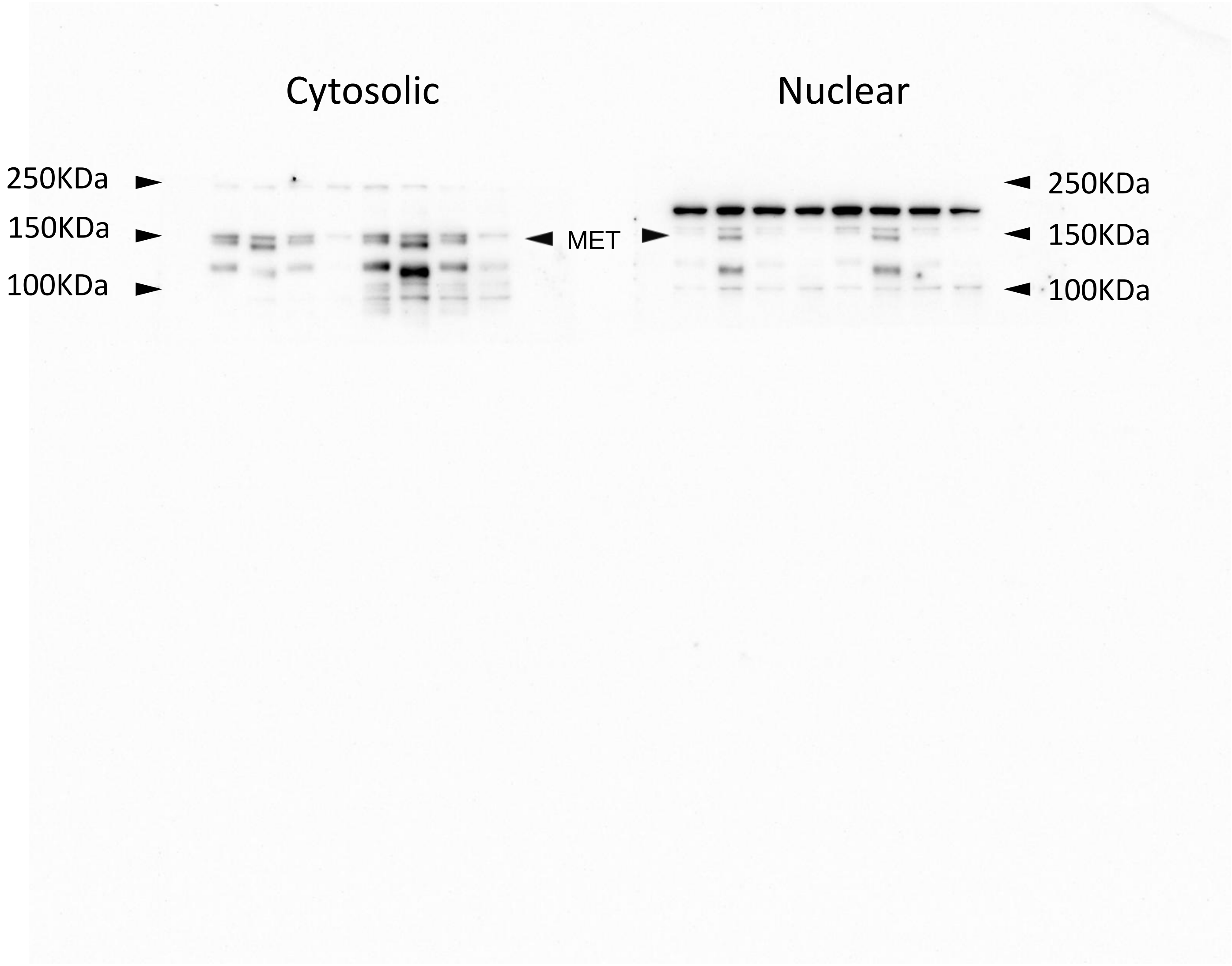
Cytosolic
Nuclear
250KDa
150KDa
100KDa
MET
250KDa
150KDa
100KDa

pERK1/2

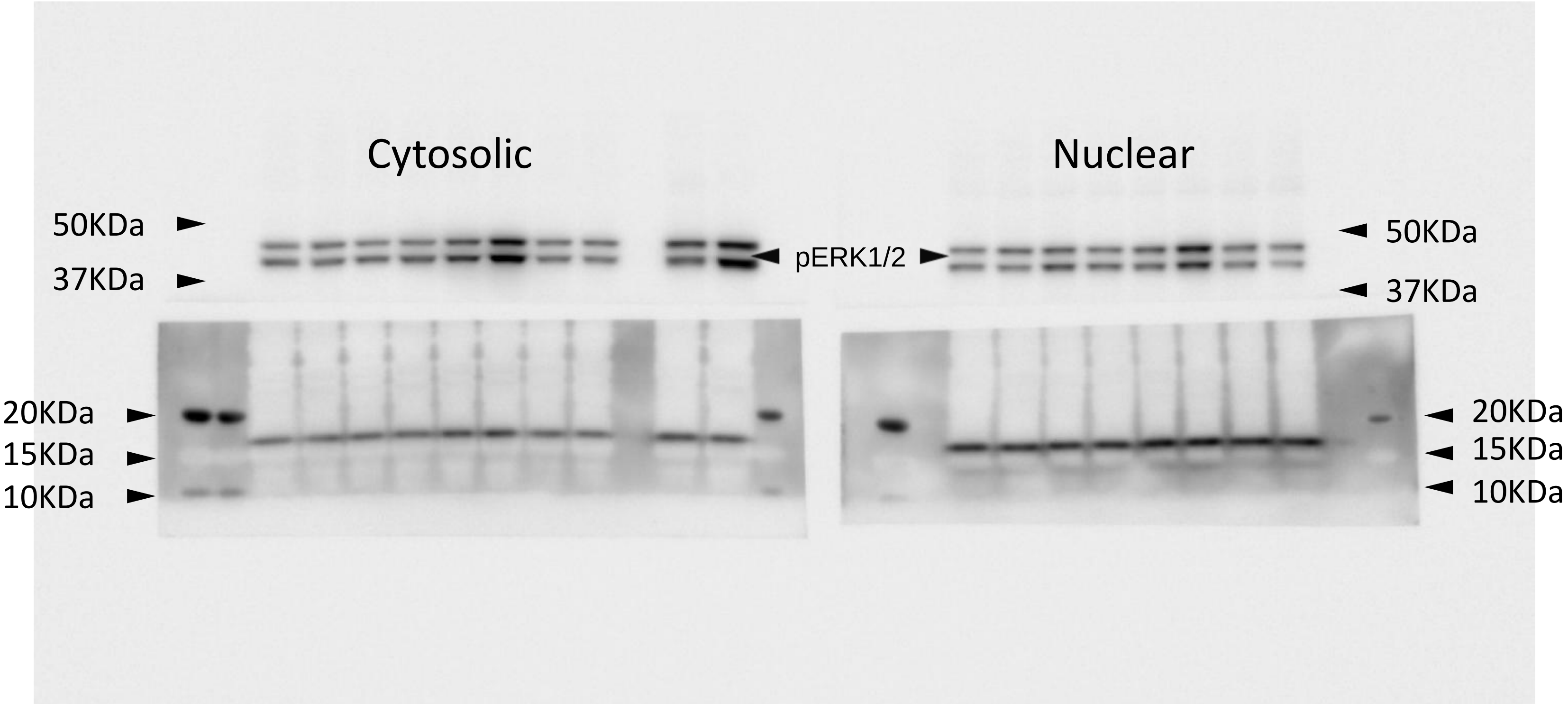

Cytosolic
Nuclear
50KDa
37KDa
pERK1/2
50KDa
37KDa
20KDa
15KDa
10KDa
20KDa
15KDa
10KDa

ERK1/2

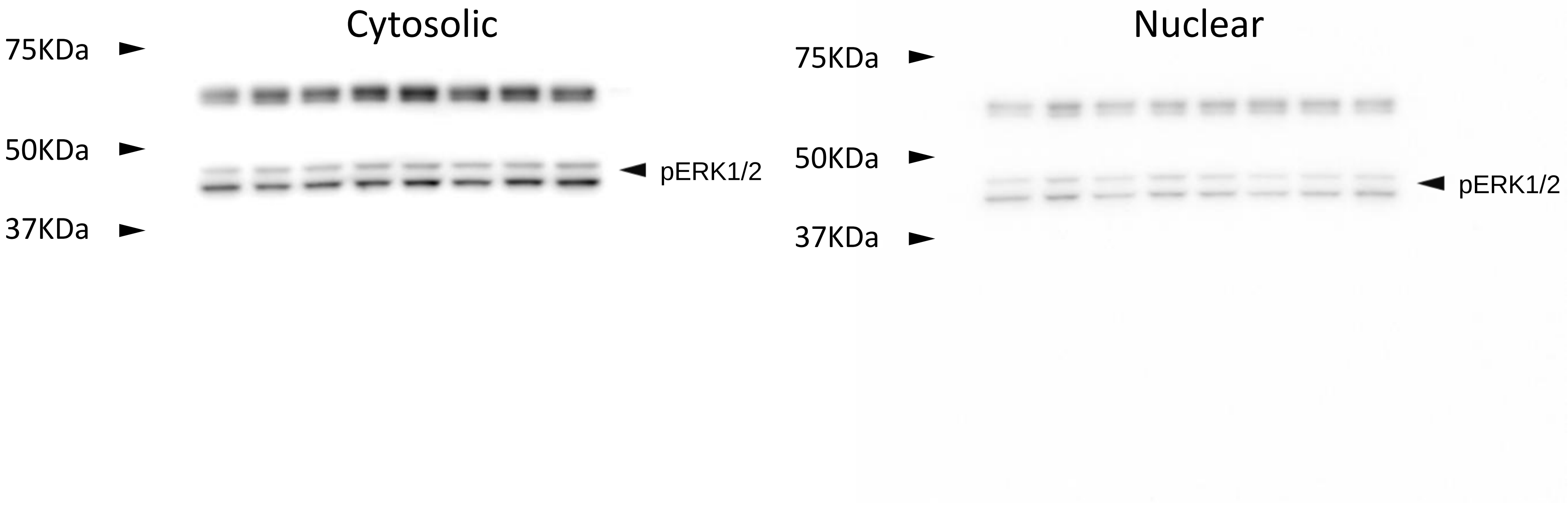
Cytosolic
75KDa
50KDa
pERK1/2
37KDa
Nuclear
75KDa
50KDa
pERK1/2
37KDa

GAPDH

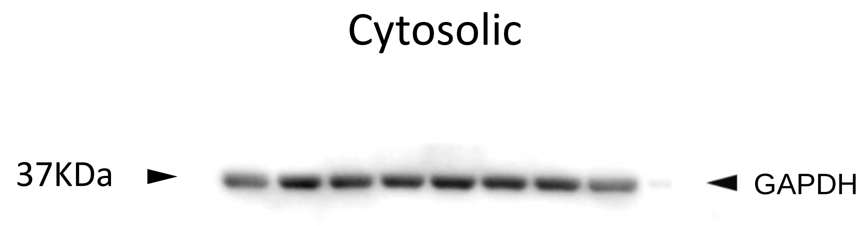

Histone H3

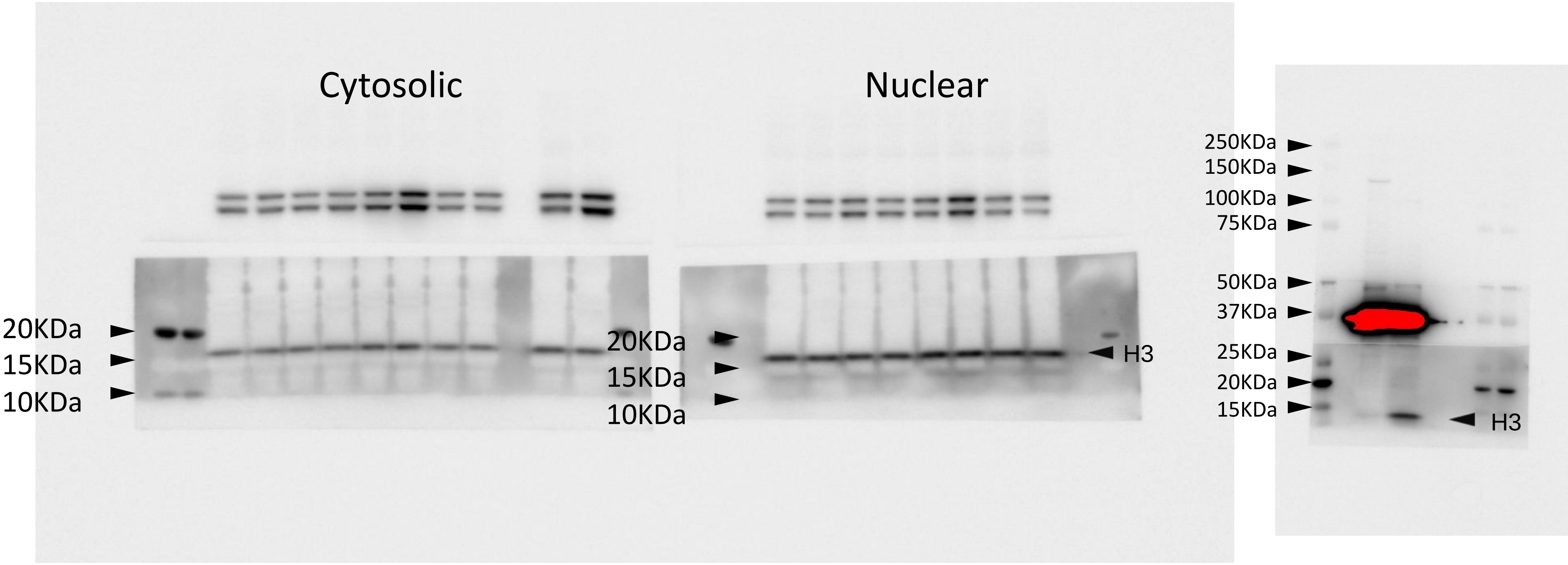
Cytosolic
Nuclear
20KDa
15KDa
10KDa
20KDa
15KDa
10KDa
H3
250KDa
150KDa
100KDa
75KDa
50KDa
37KDa
25KDa
20KDa
15KDa
H3

Vinculin & AKT

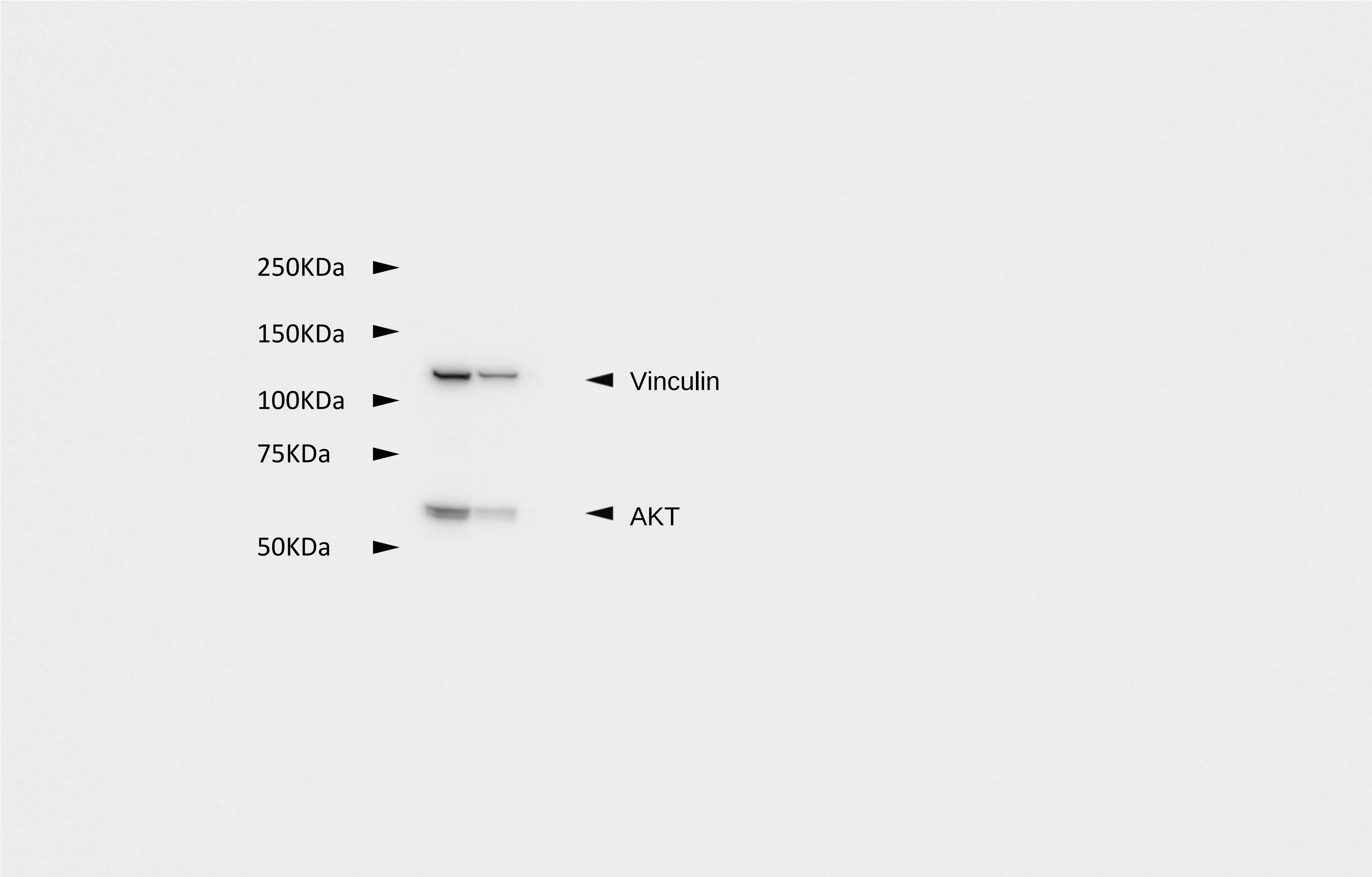

Supplement: Supplementary file 1 [file cancers-14-01378-s001.zip › Supplementary Figure S6 - Original western blots.zip!/Supplementary Figure S6 - Original western blots/(E) Fig 3d+supp. Fig S5.pdf]
