# Supplementary material for: MET Exon 14 Splice-Site Mutations Preferentially Activate KRAS Signaling to Drive Tumourigenesis
Source: Cancers (Basel). 2022 Mar 8;14(6):1378. doi: 10.3390/cancers14061378 (PMC8946549; doi:10.3390/cancers14061378)
Supplement: Supplementary file 1 [file cancers-14-01378-s001.zip › Supplementary Figure S6 - Original western blots.zip!/Supplementary Figure S6 - Original western blots/(H) Fig 4b.pdf]

pMEK1/2

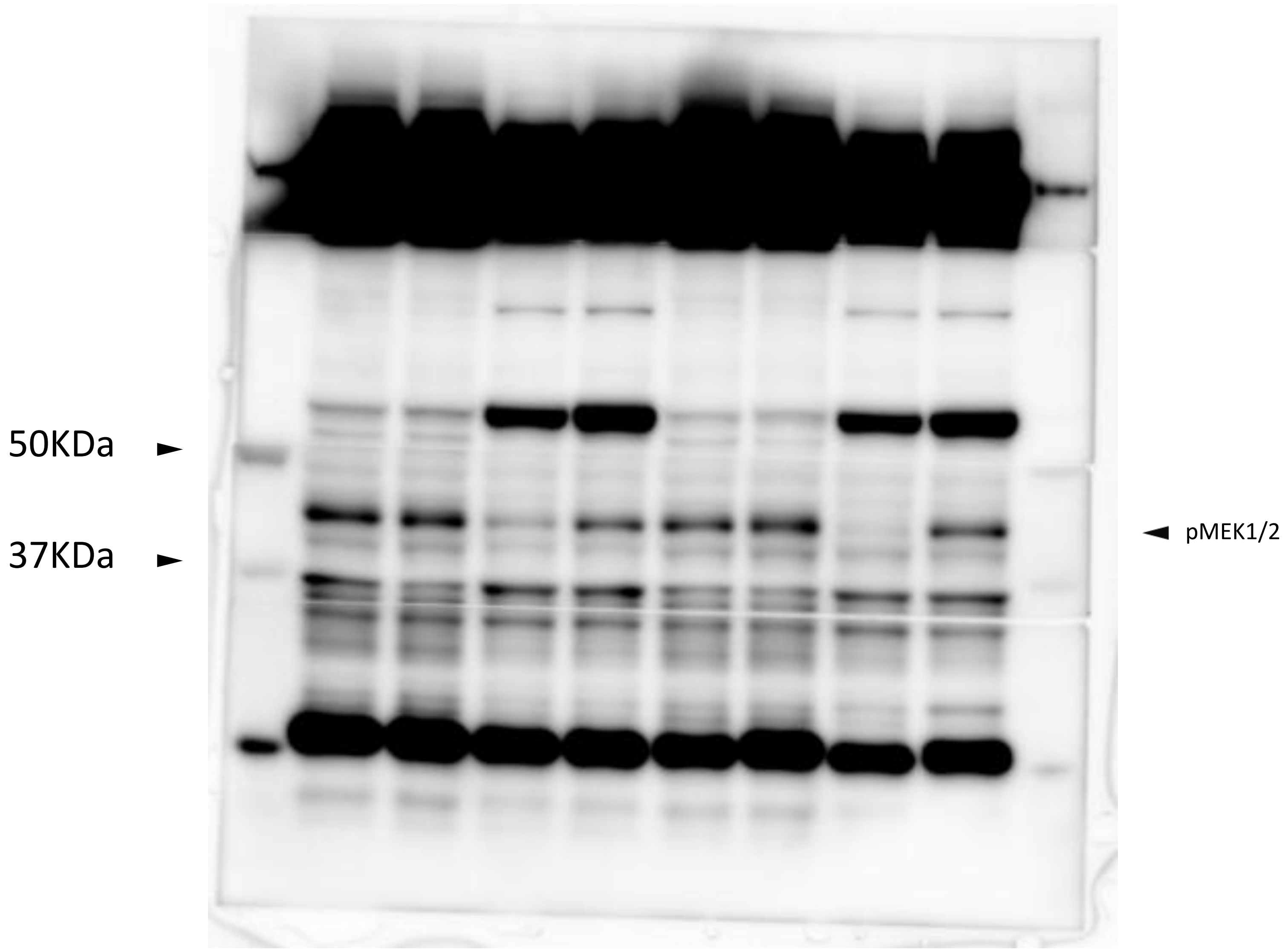

MEK1/2

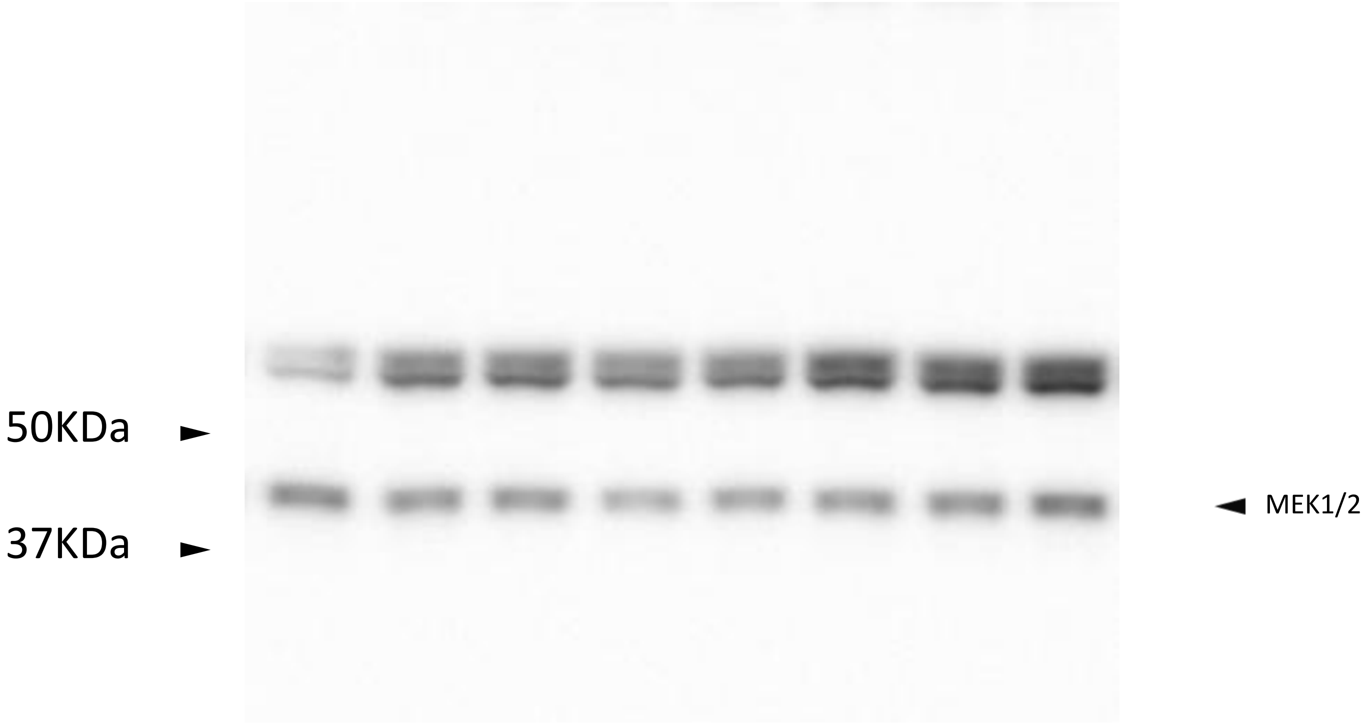

pERK1/2

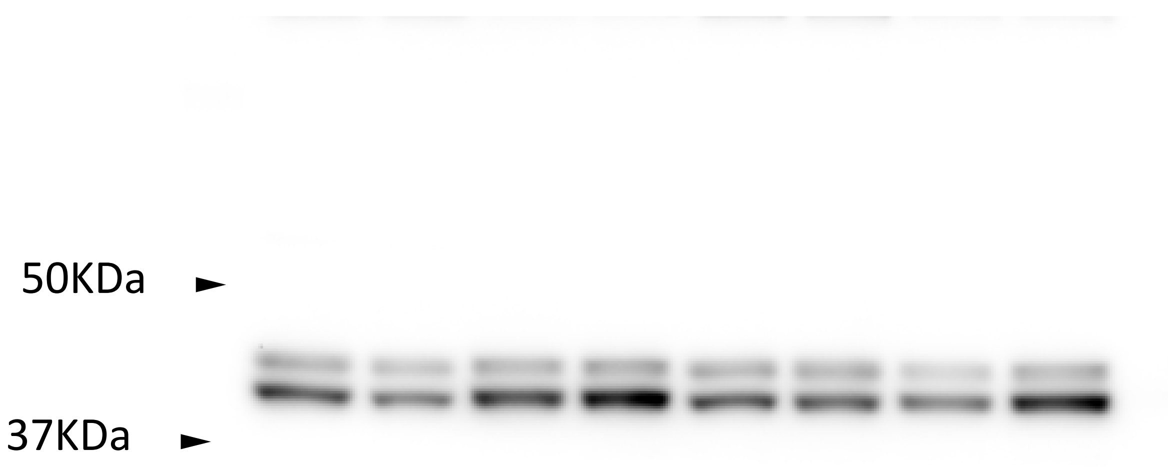

ERK1/2

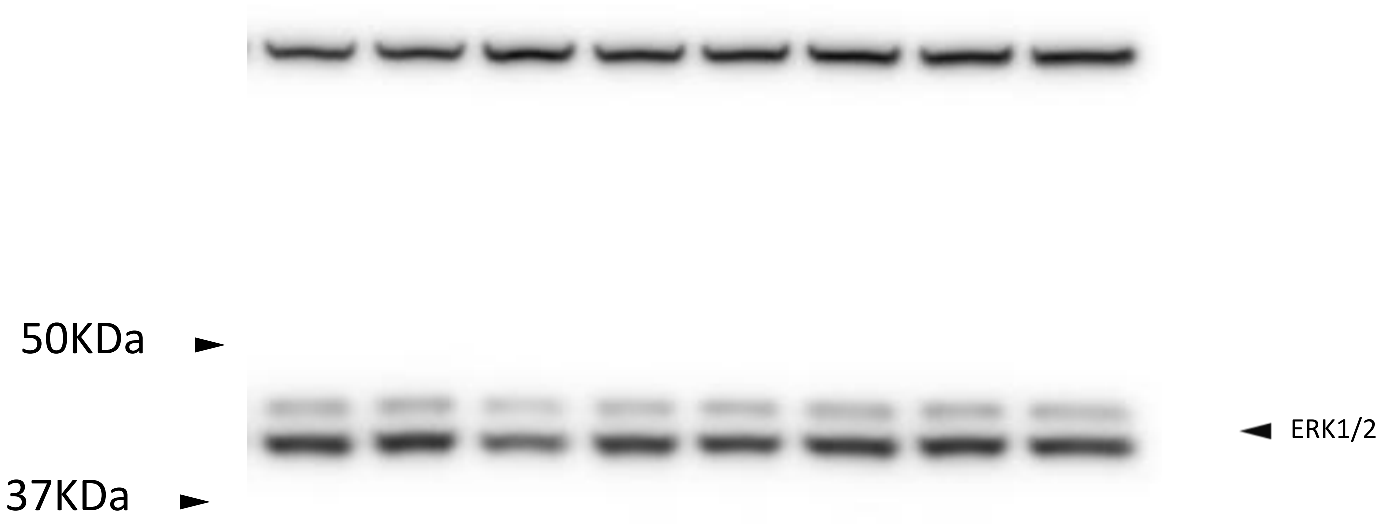

Vinculin

150KDa ►

100KDa ►

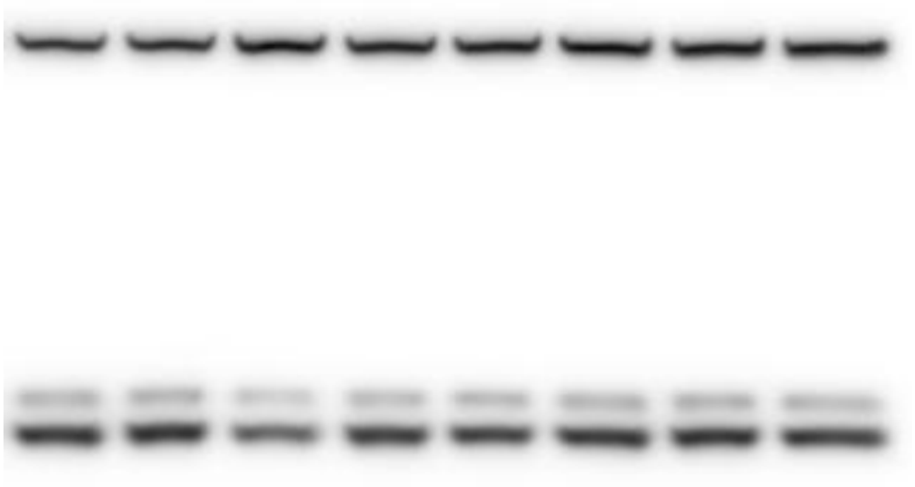

◄ Vinculin
