# Supplementary material for: MET Exon 14 Splice-Site Mutations Preferentially Activate KRAS Signaling to Drive Tumourigenesis
Source: Cancers (Basel). 2022 Mar 8;14(6):1378. doi: 10.3390/cancers14061378 (PMC8946549; doi:10.3390/cancers14061378)

p-MET Y1234 & pSTAT3 Y705

No Dox

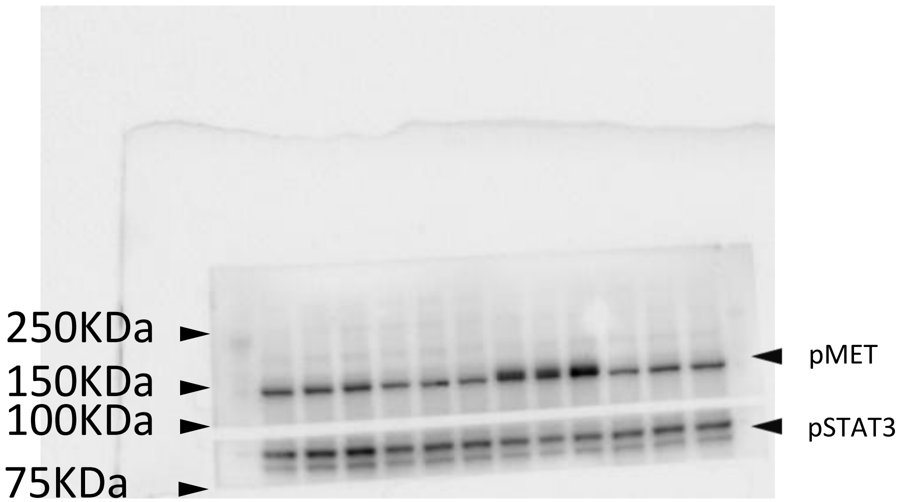

200ng/mL Dox

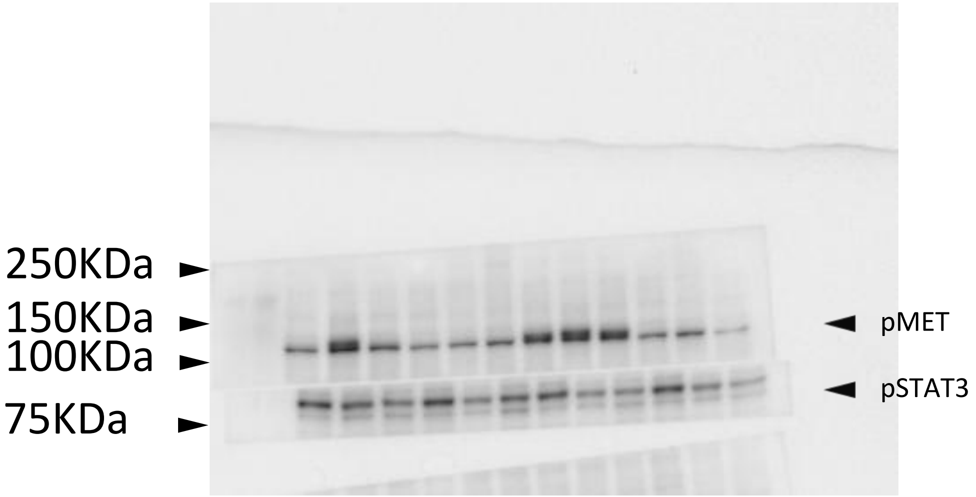

MET

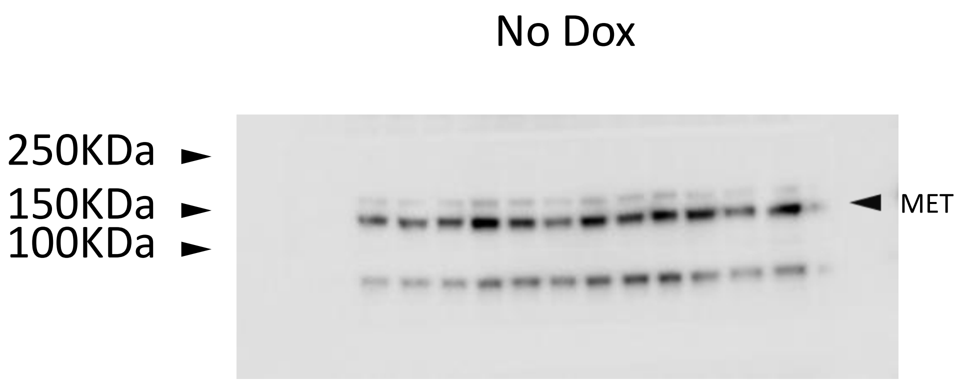
No Dox
250KDa
150KDa
100KDa
MET

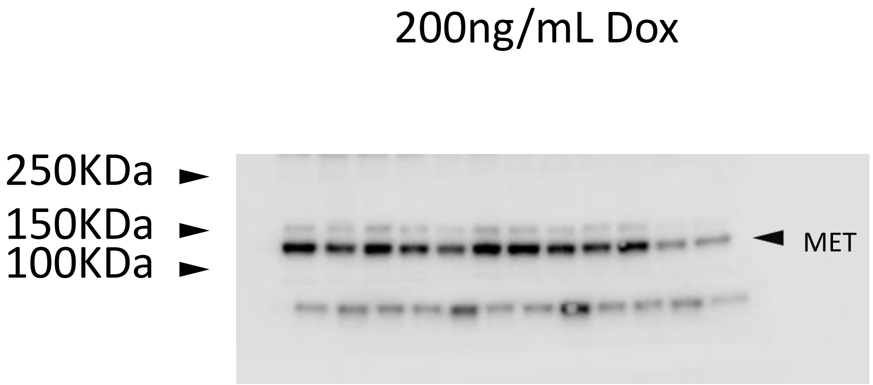
200ng/mL Dox
250KDa
150KDa
100KDa
MET

STAT3

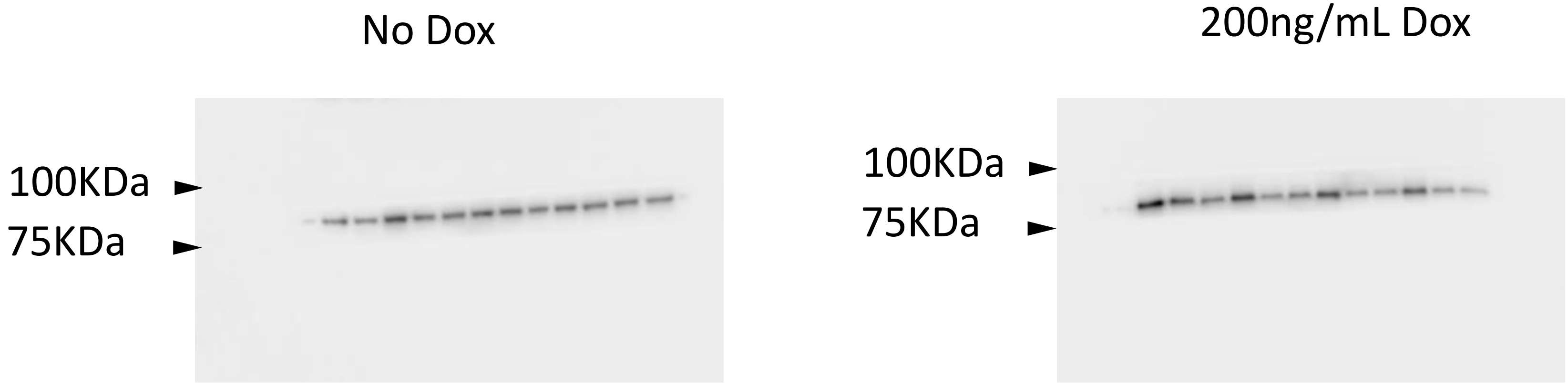

RAS

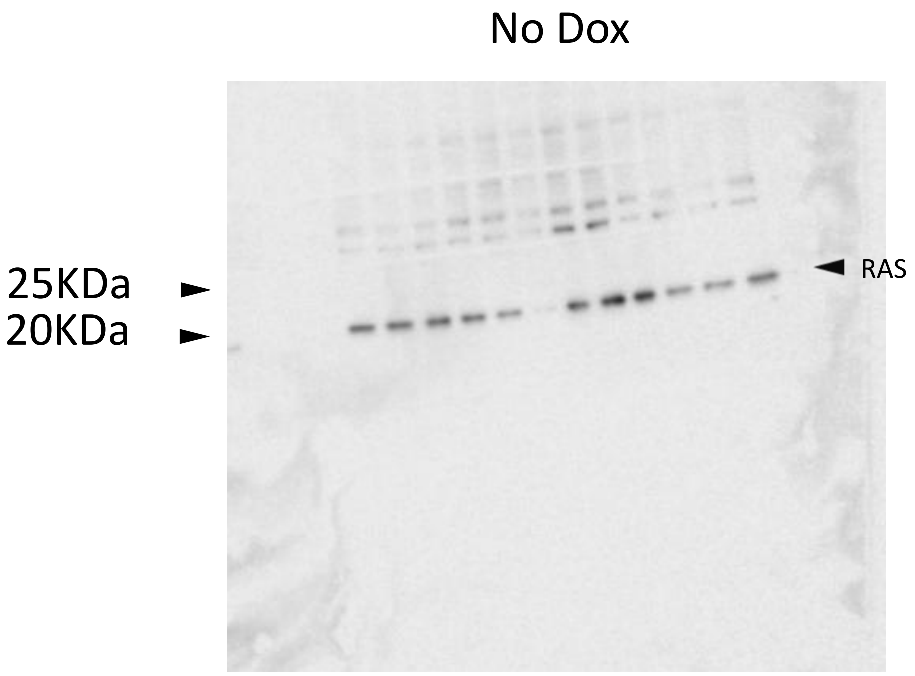

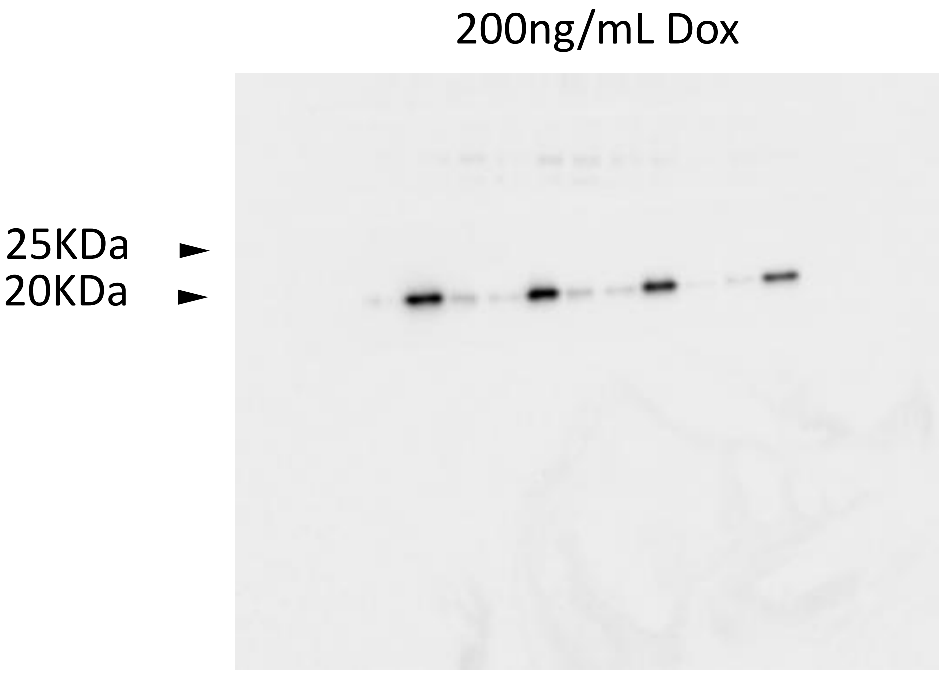

pMEK1/2

No Dox

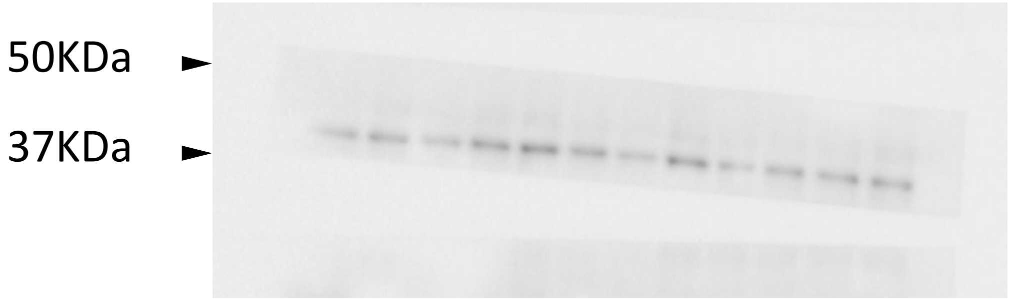

200ng/mL Dox

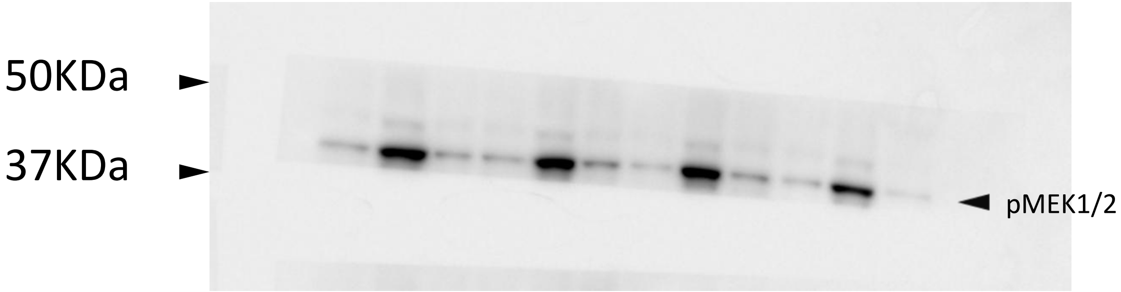

MEK1/2

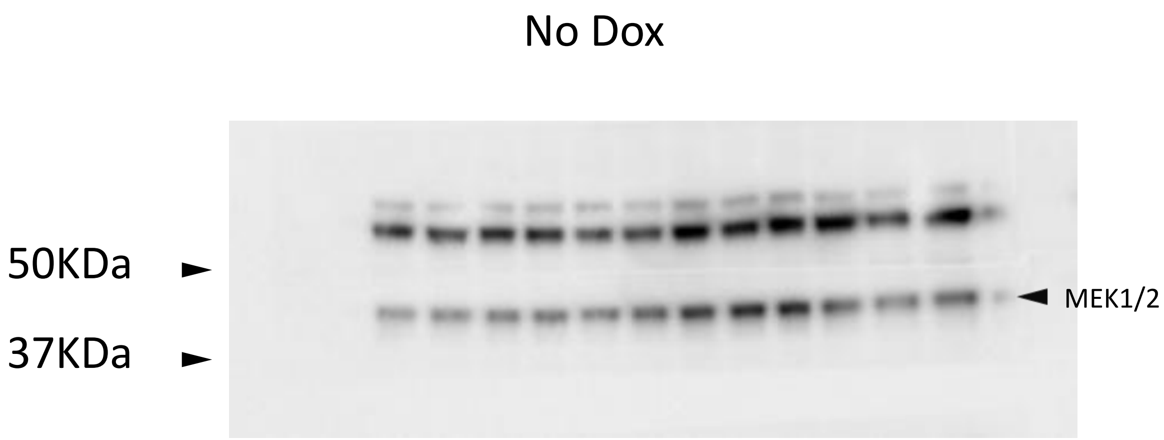

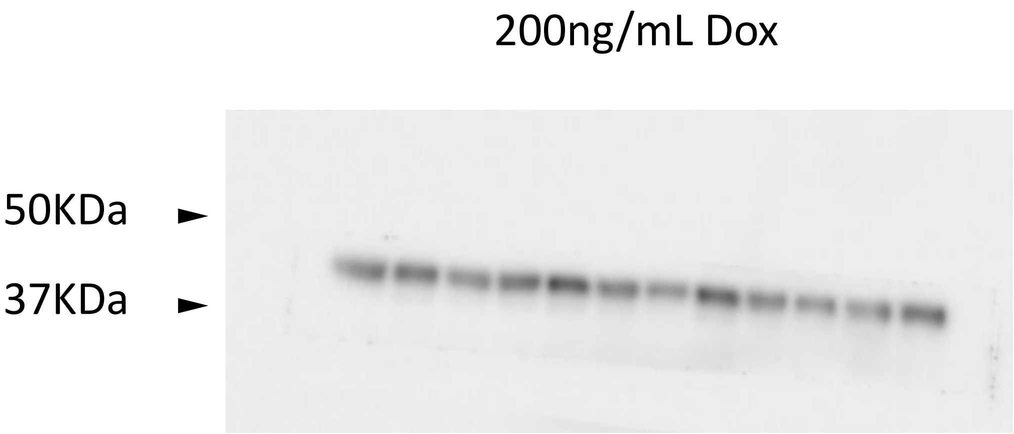

pERK1/2

No Dox

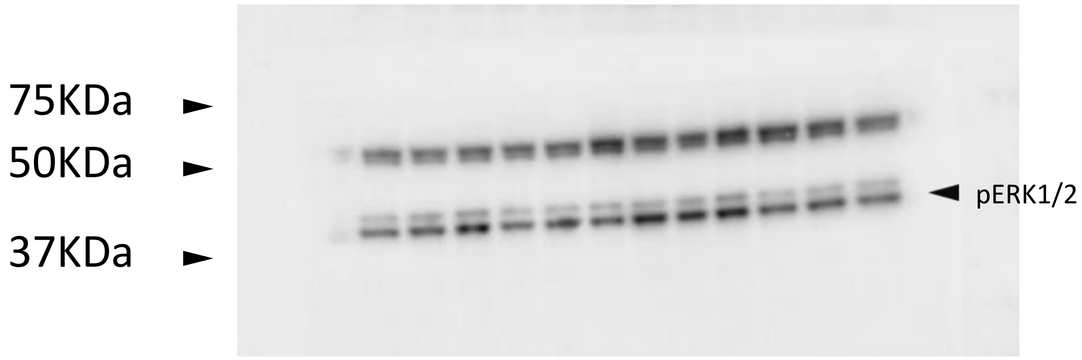

200ng/mL Dox

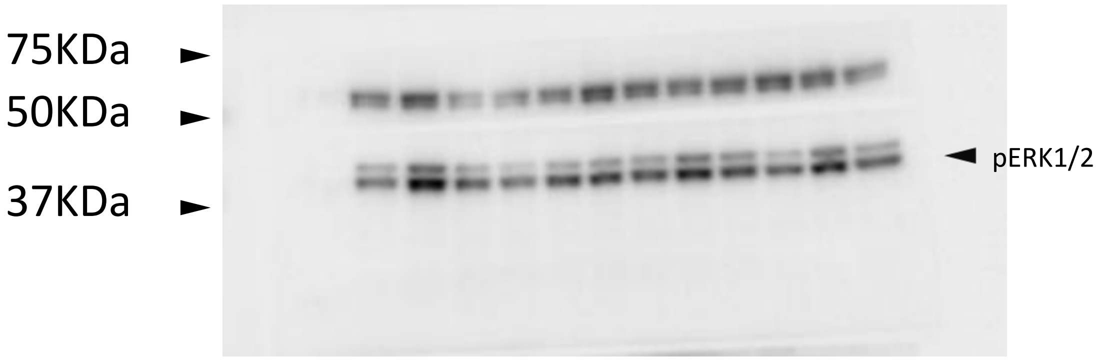

ERK1/2

No Dox

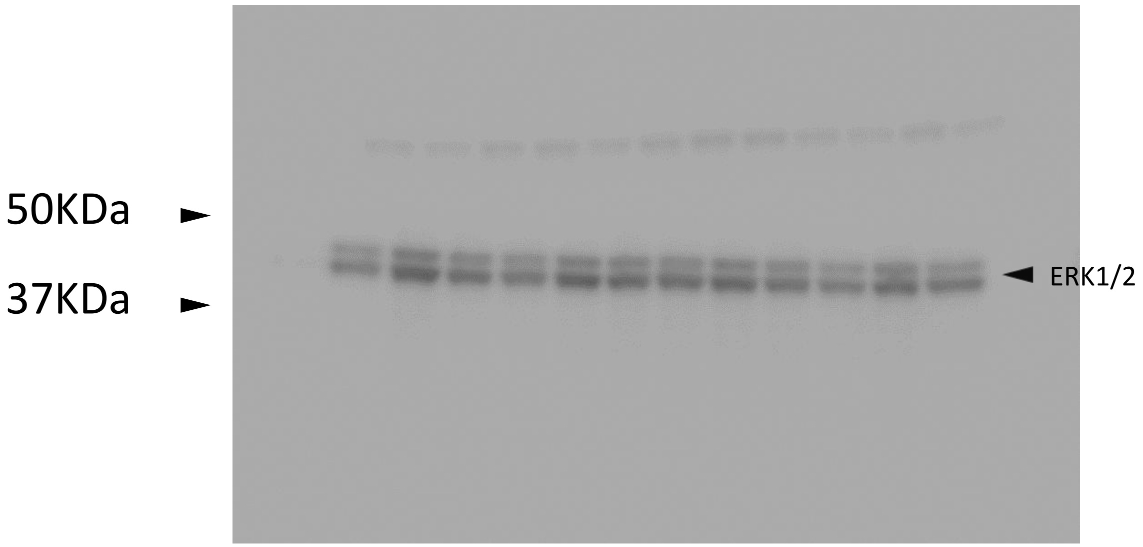

200ng/mL Dox

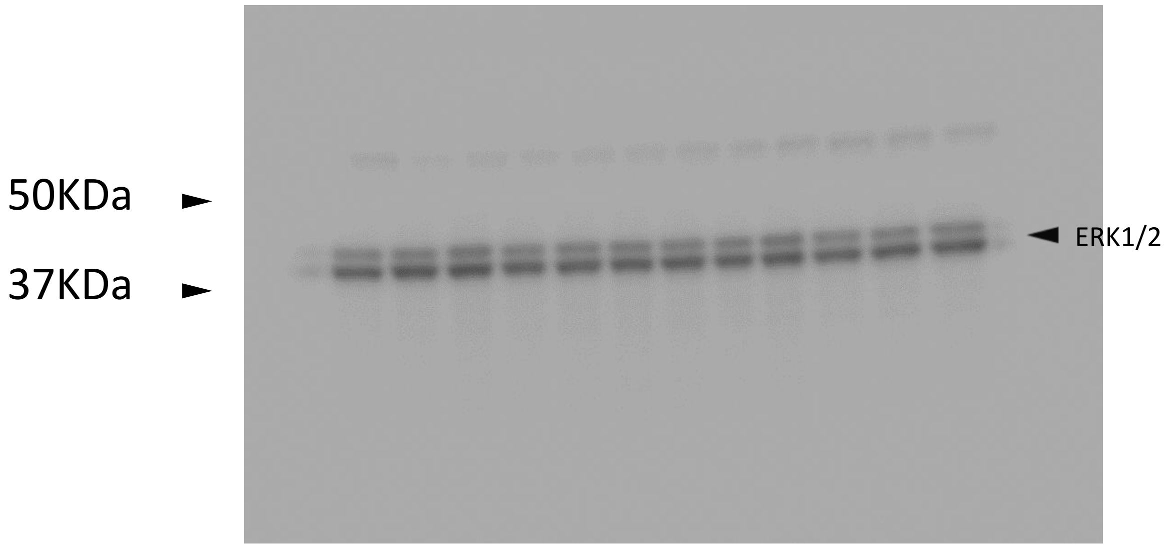

Vinculin

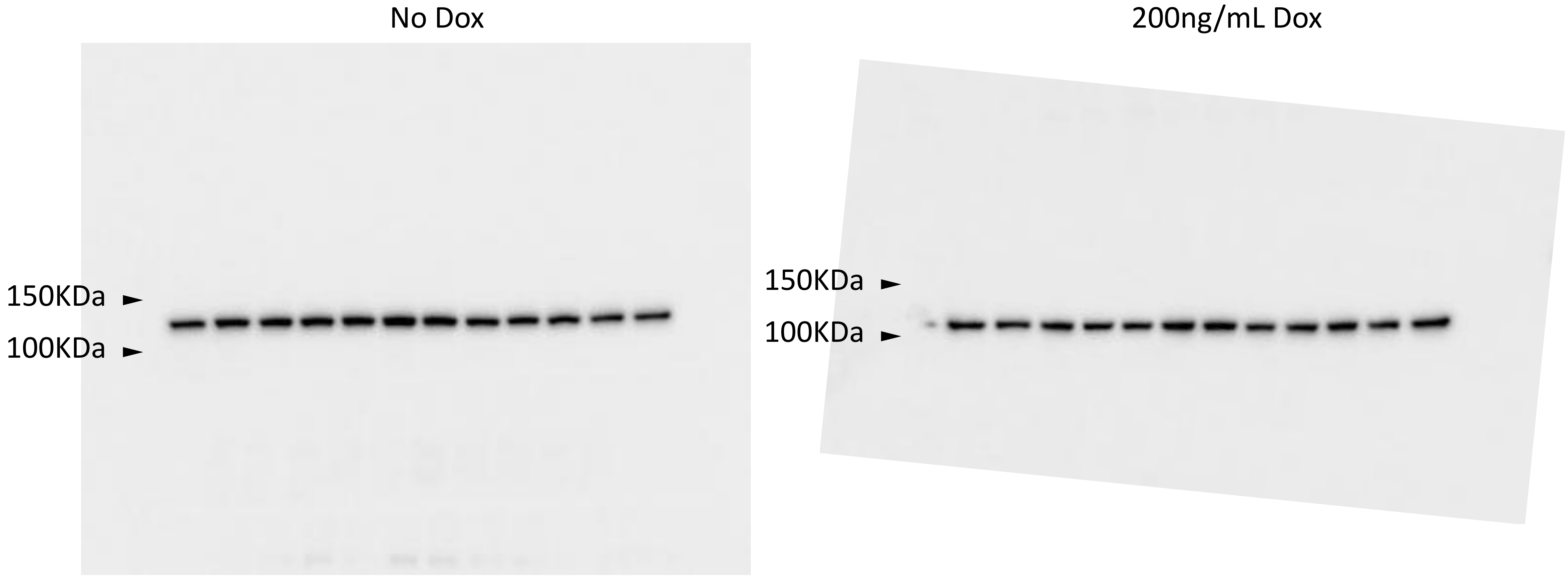

Supplement: Supplementary file 1 [file cancers-14-01378-s001.zip › Supplementary Figure S6 - Original western blots.zip!/Supplementary Figure S6 - Original western blots/(I) Fig 5i.pdf]
