# Supplementary material for: MET Exon 14 Splice-Site Mutations Preferentially Activate KRAS Signaling to Drive Tumourigenesis
Source: Cancers (Basel). 2022 Mar 8;14(6):1378. doi: 10.3390/cancers14061378 (PMC8946549; doi:10.3390/cancers14061378)

pMET Y1234

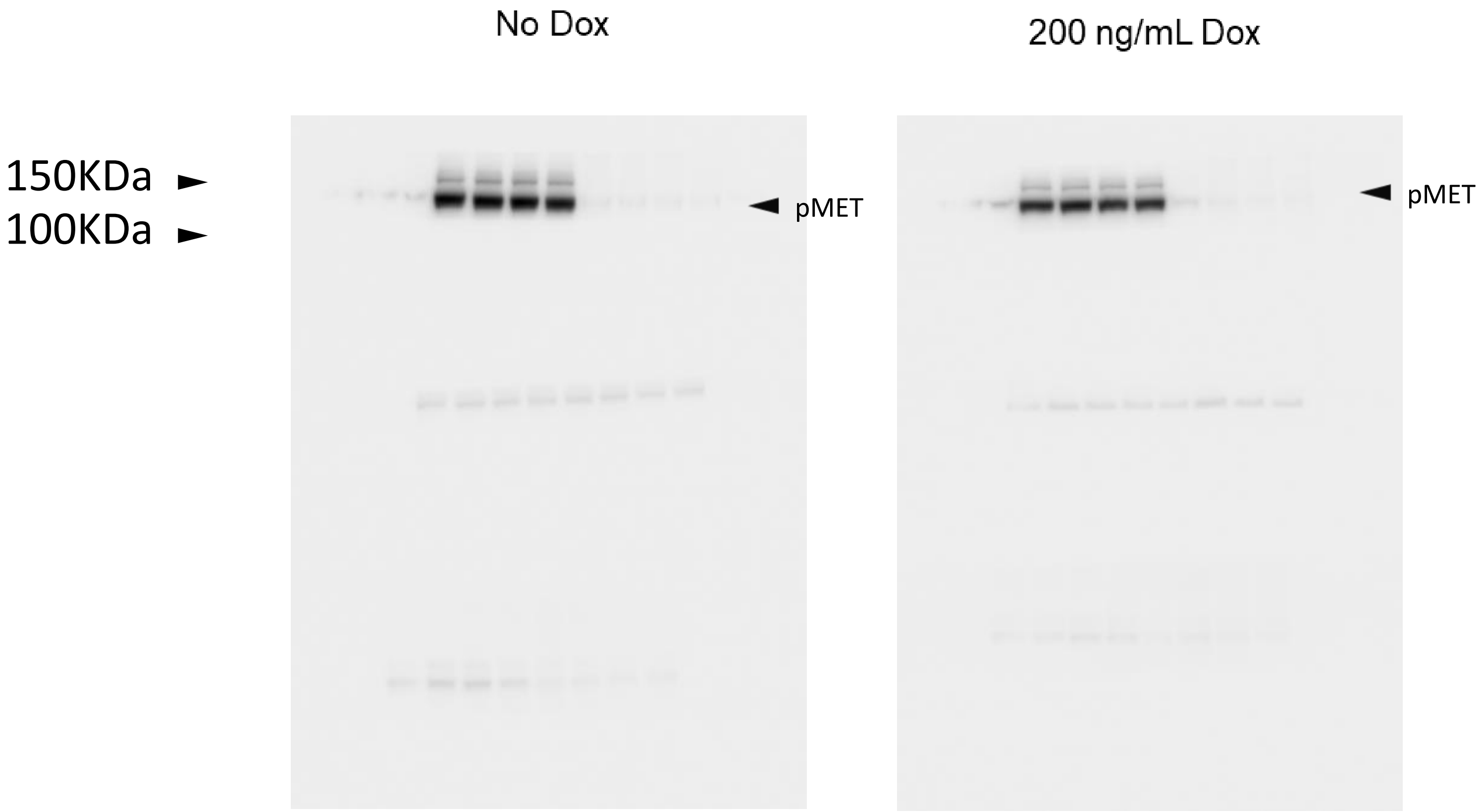

MET

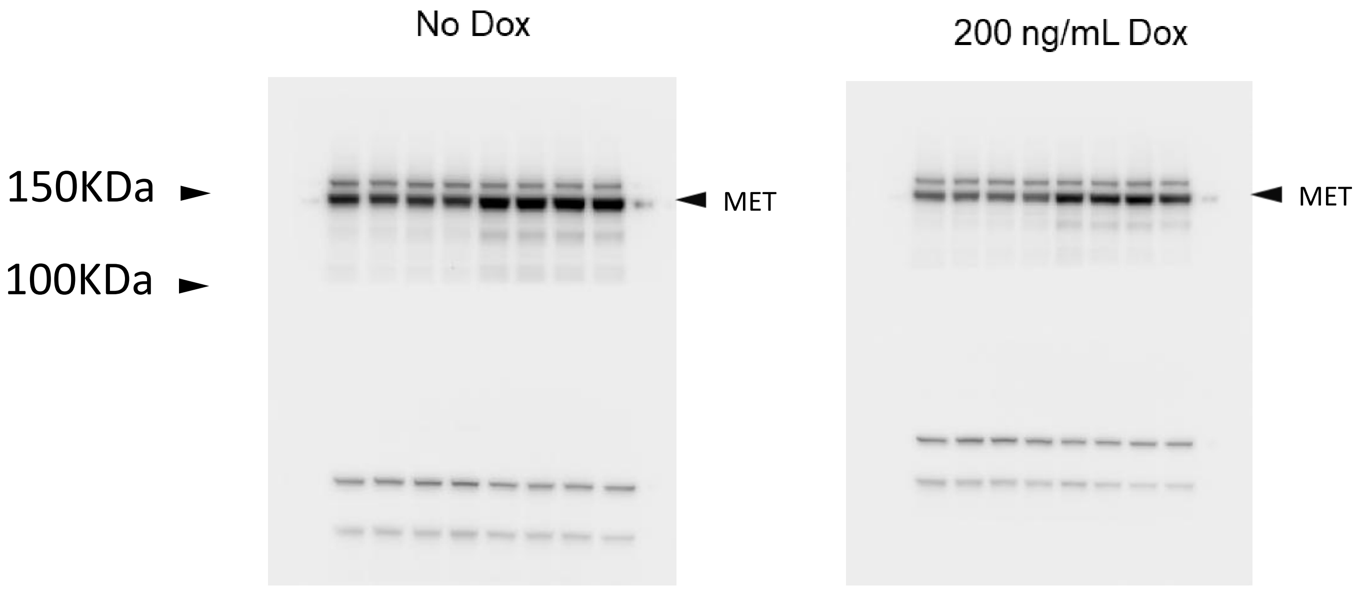

pSTAT3 Y705

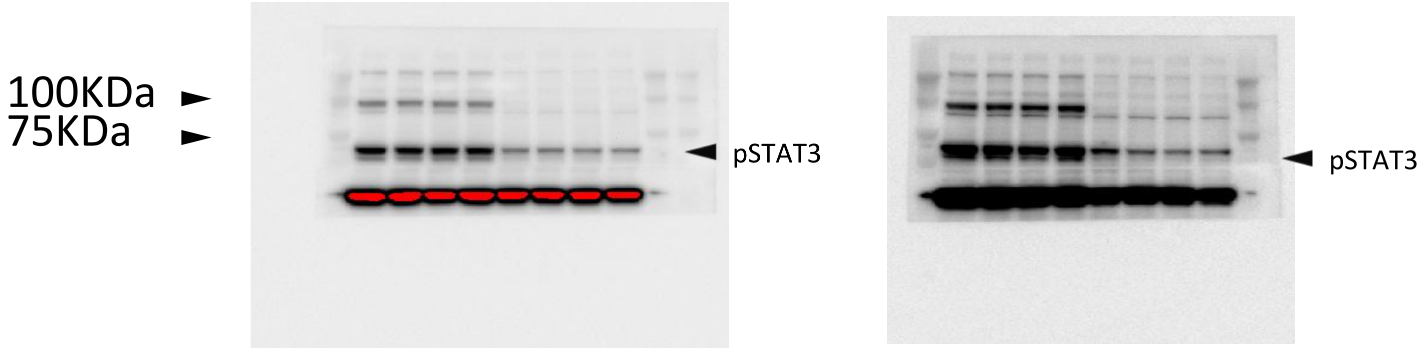

STAT3

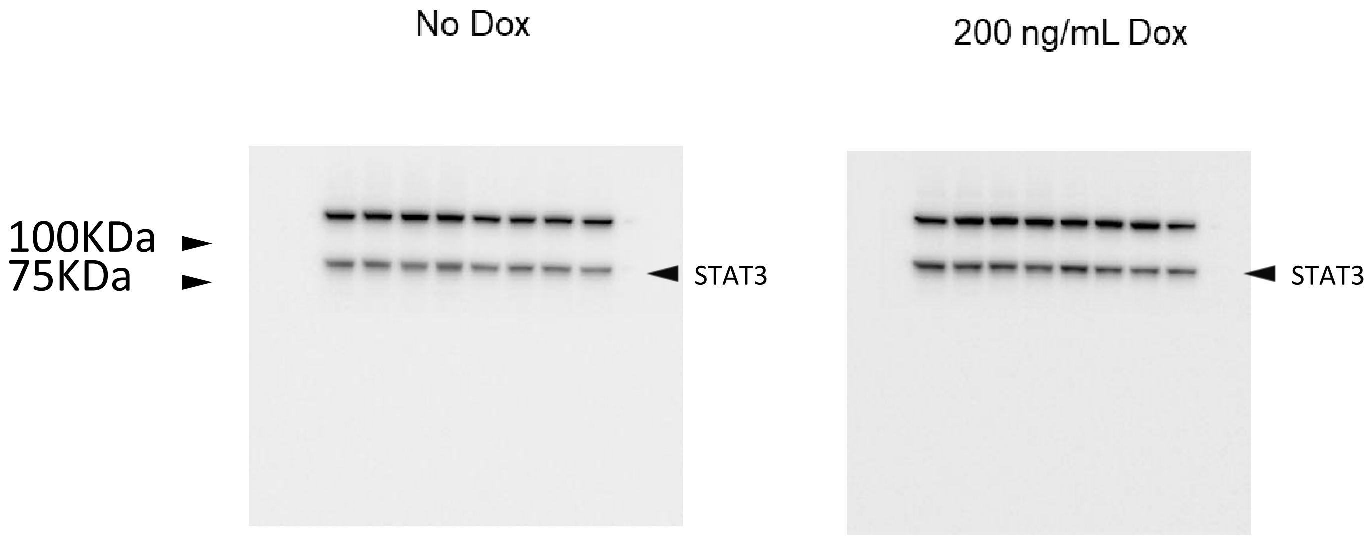

p110α

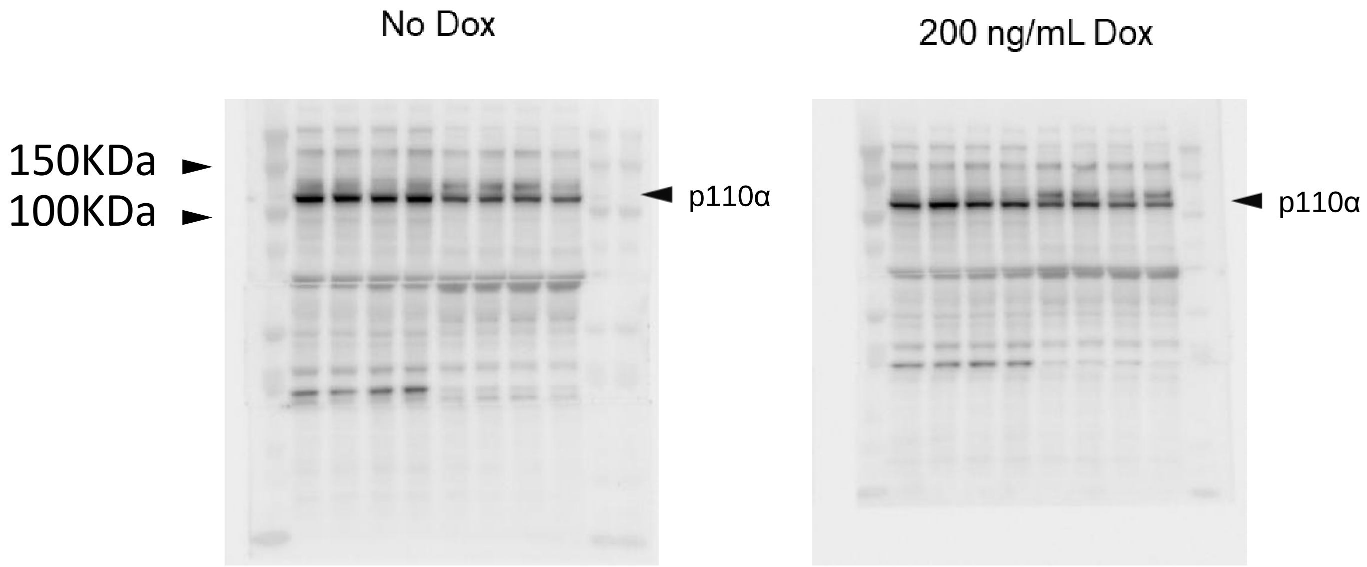

pAKT S473

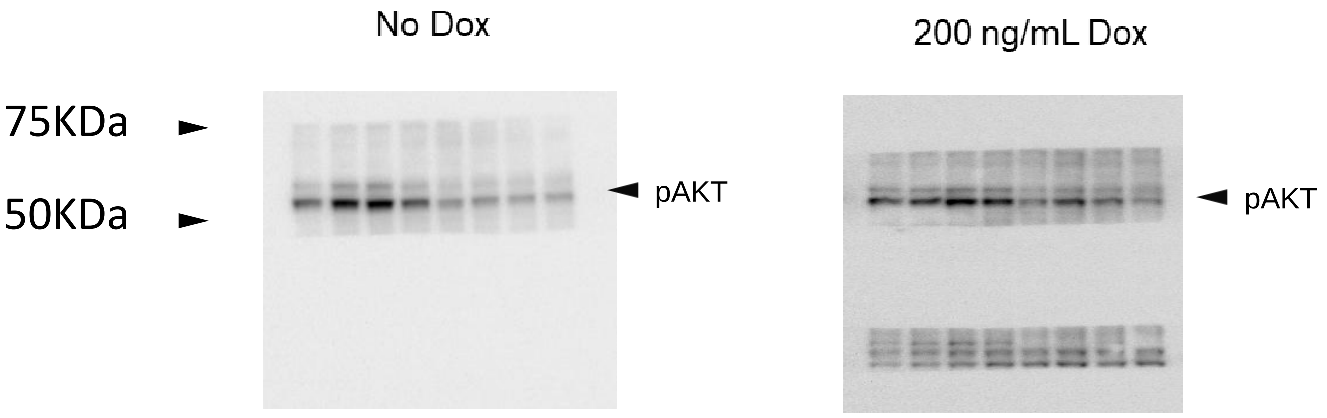

AKT

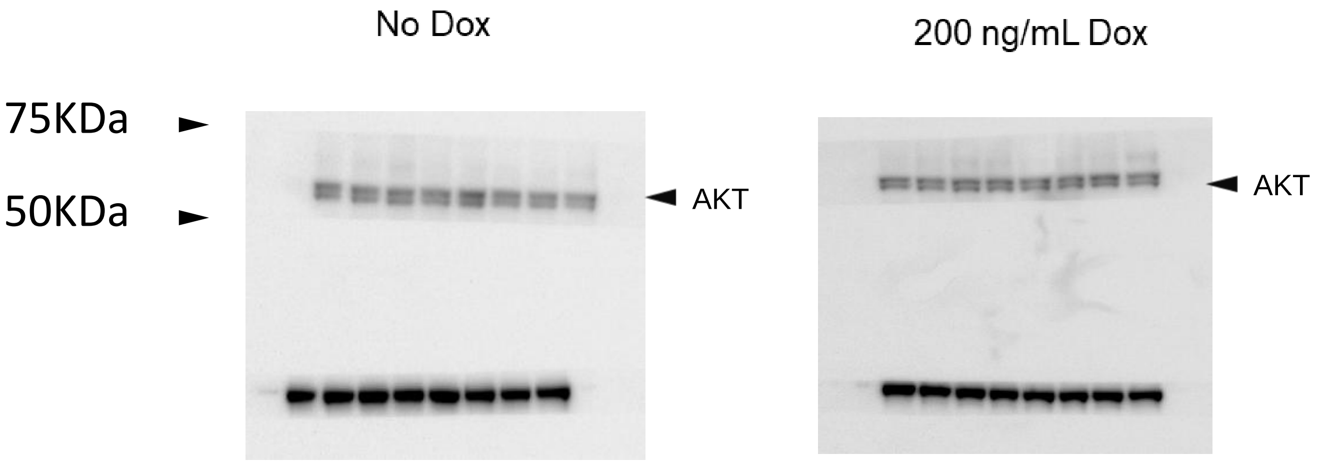

RAS

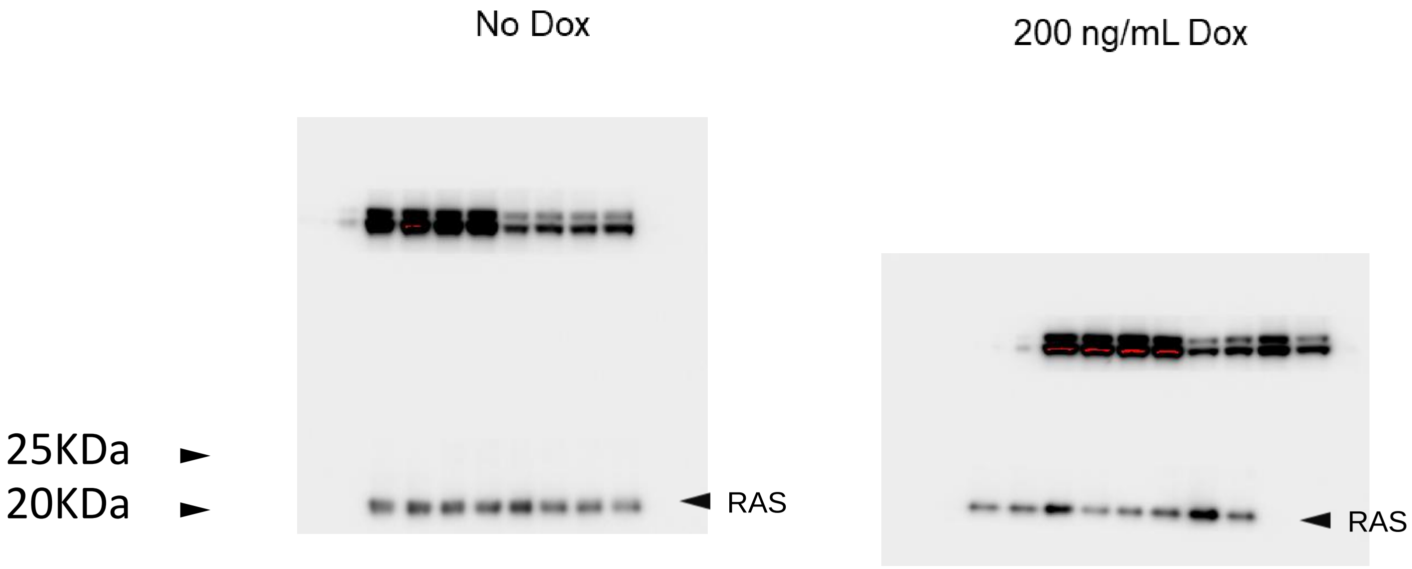

pMEK1/2

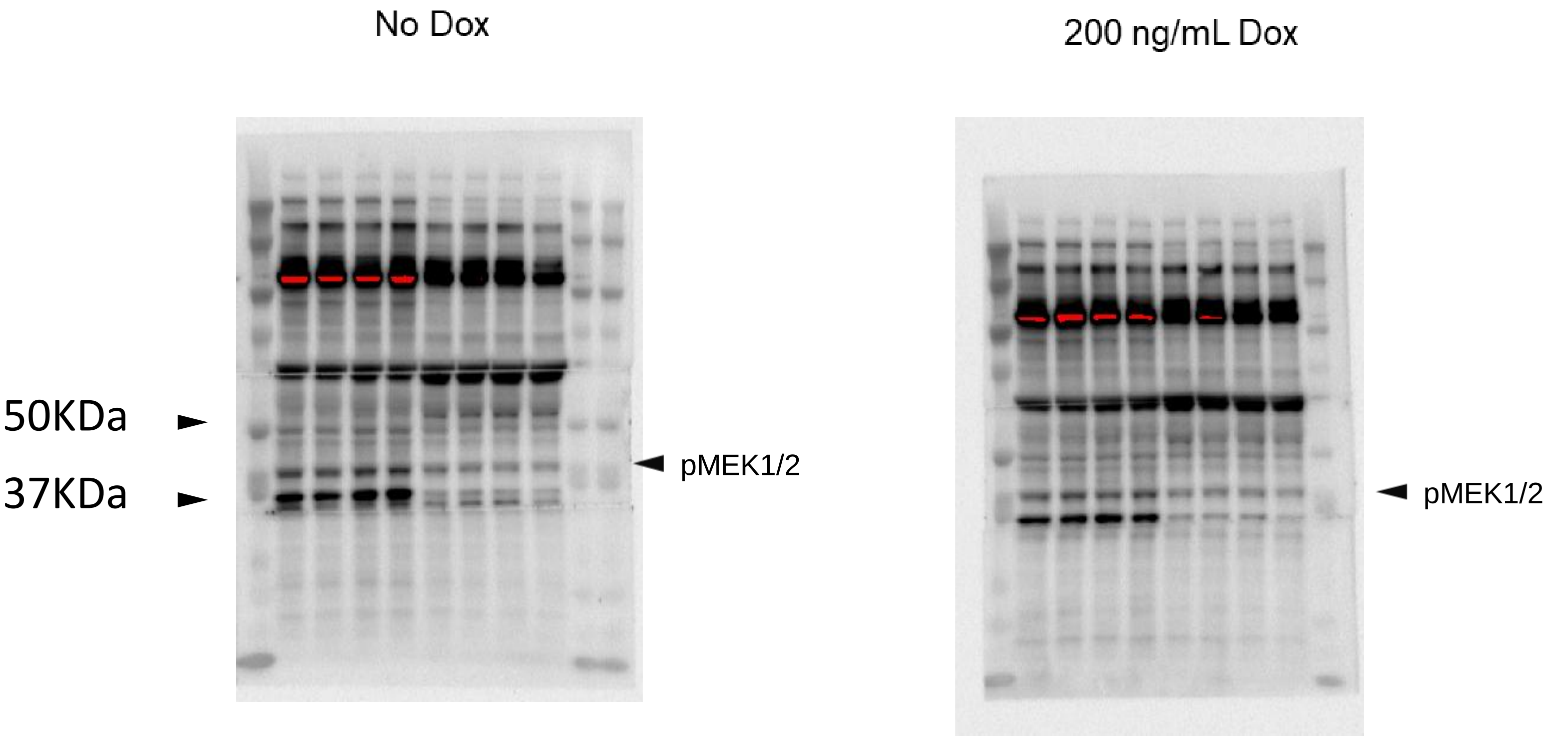

MEK1/2

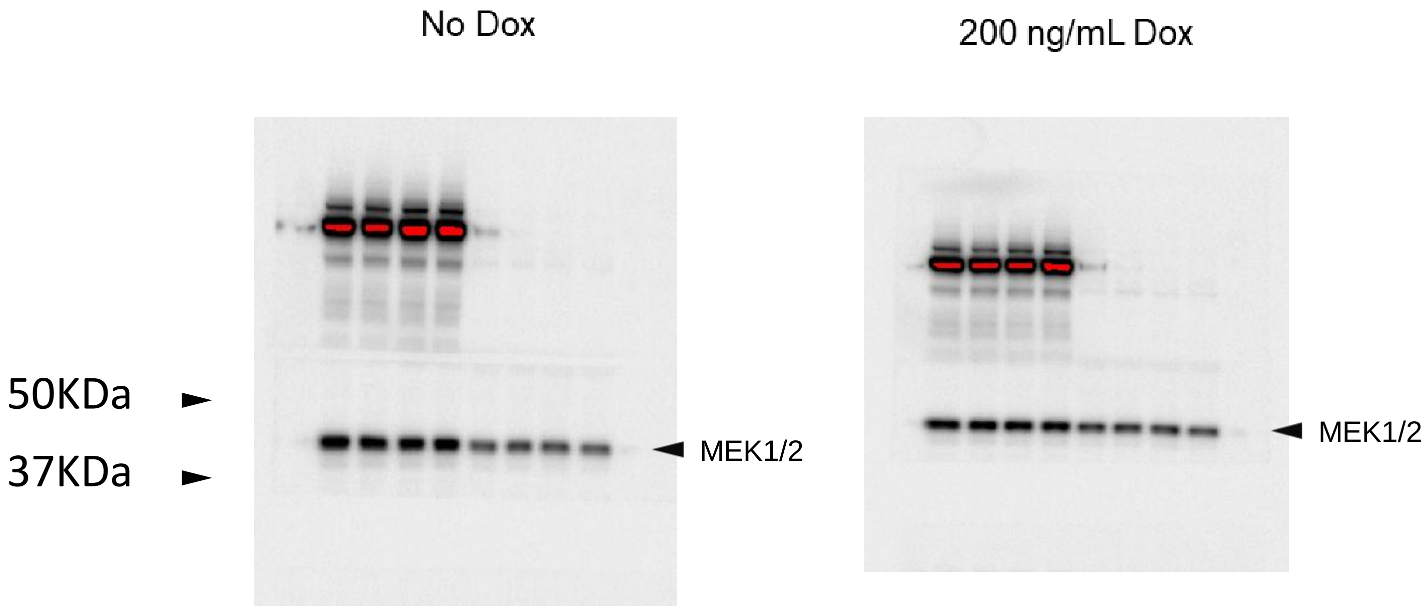

pERK1/2

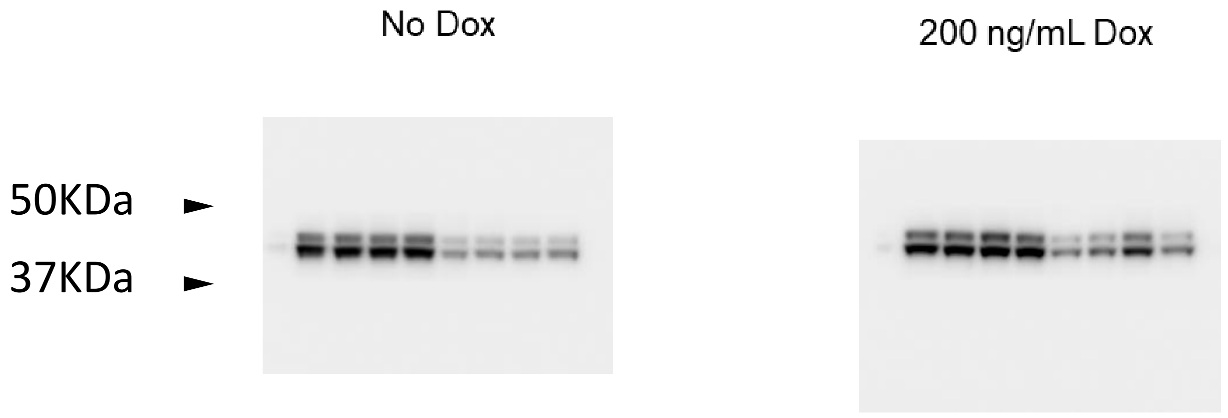

ERK1/2

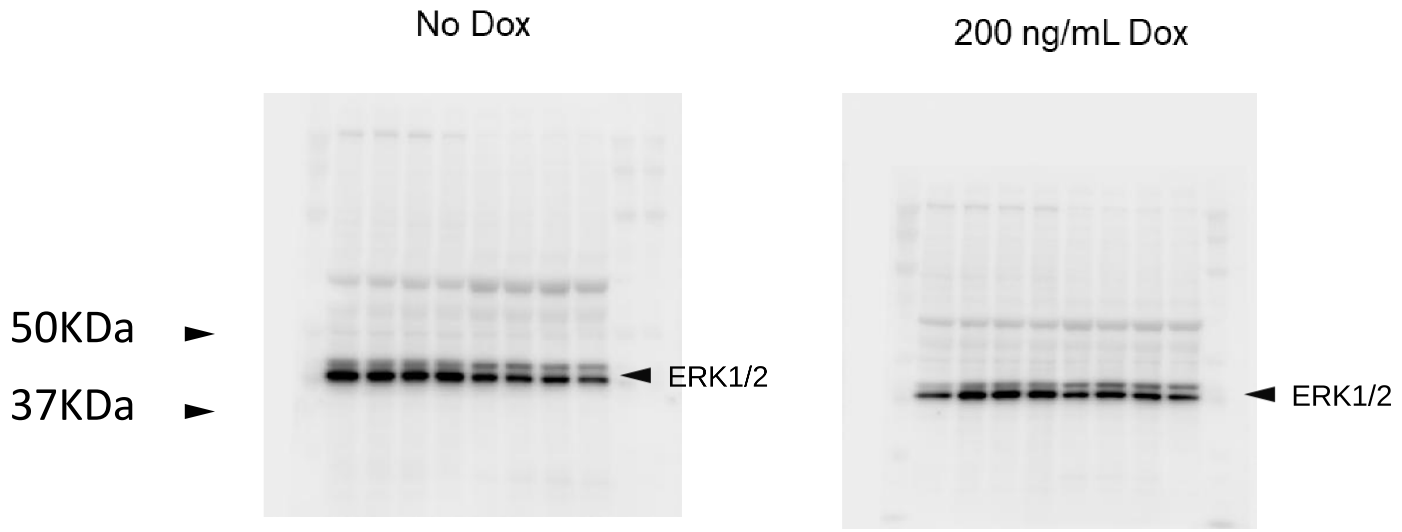

Vinculin

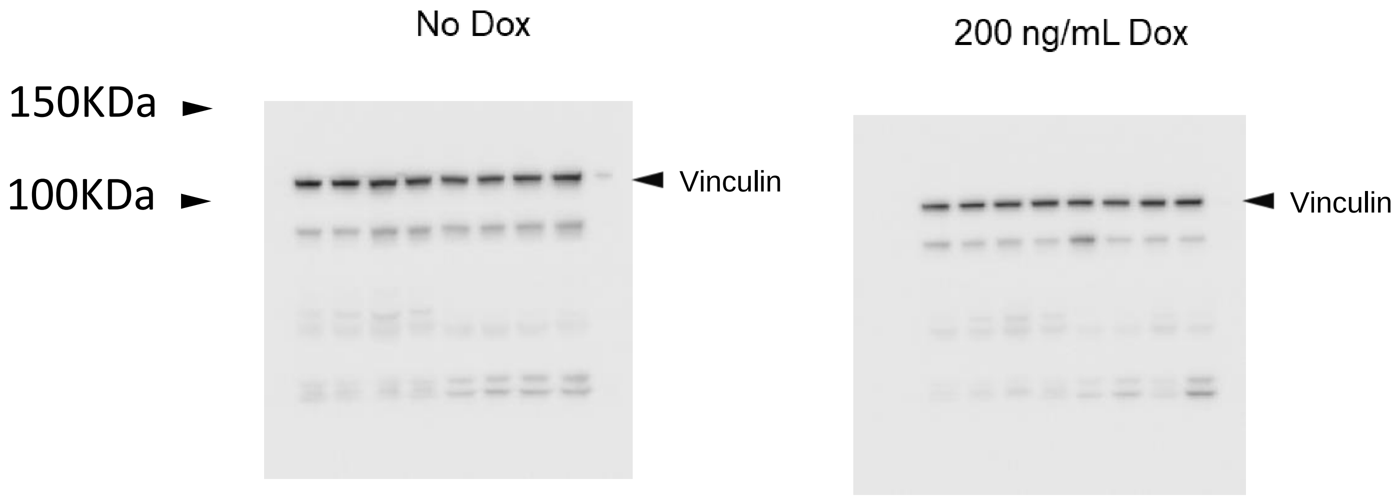

Supplement: Supplementary file 1 [file cancers-14-01378-s001.zip › Supplementary Figure S6 - Original western blots.zip!/Supplementary Figure S6 - Original western blots/(J) Fig 5l.pdf]
