# Supplementary material for: MET Exon 14 Splice-Site Mutations Preferentially Activate KRAS Signaling to Drive Tumourigenesis
Source: Cancers (Basel). 2022 Mar 8;14(6):1378. doi: 10.3390/cancers14061378 (PMC8946549; doi:10.3390/cancers14061378)

p-mTOR, pERK1/2

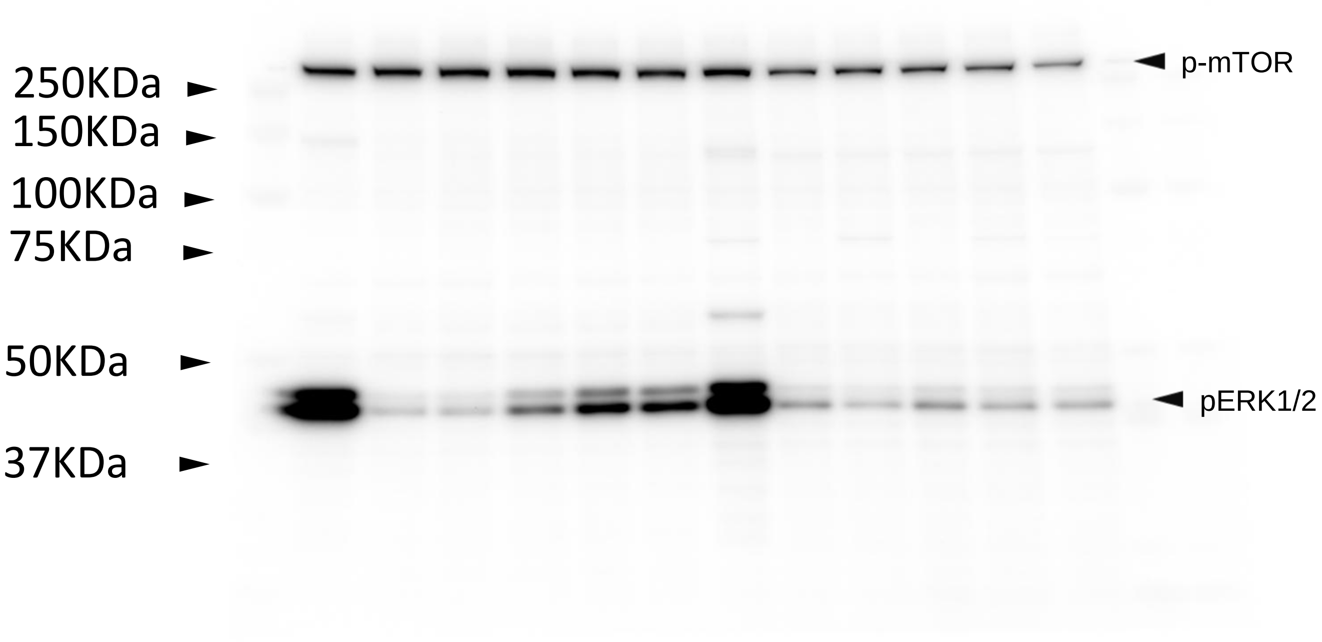

pMET_Y1234, pAKT_S473

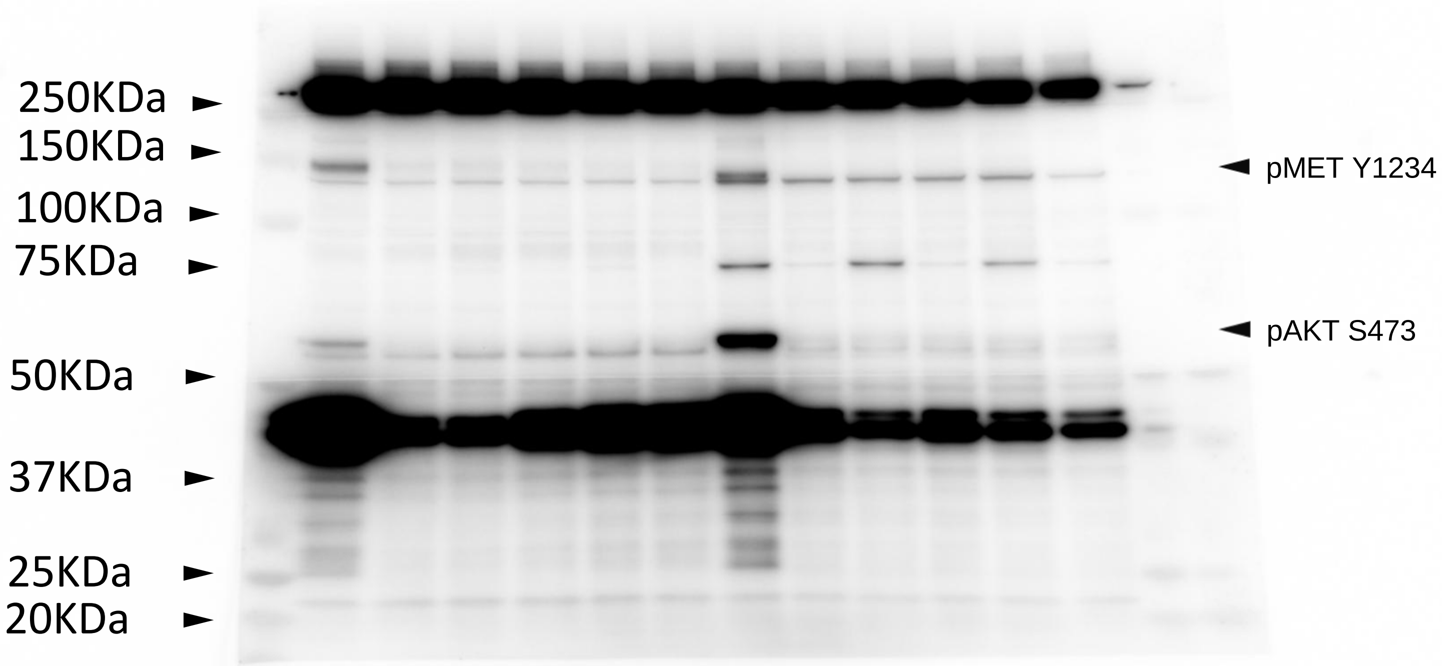

MET

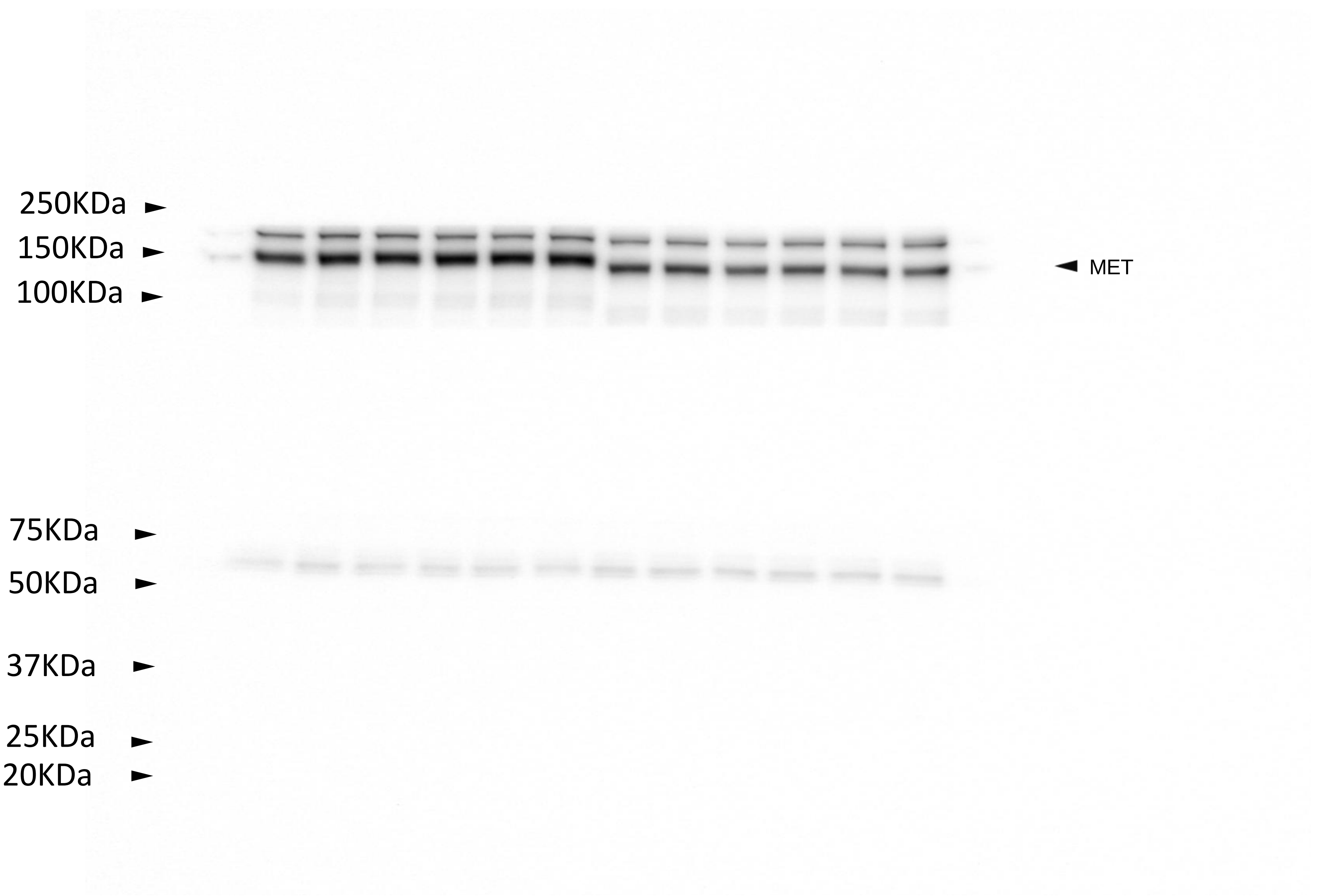

AKT

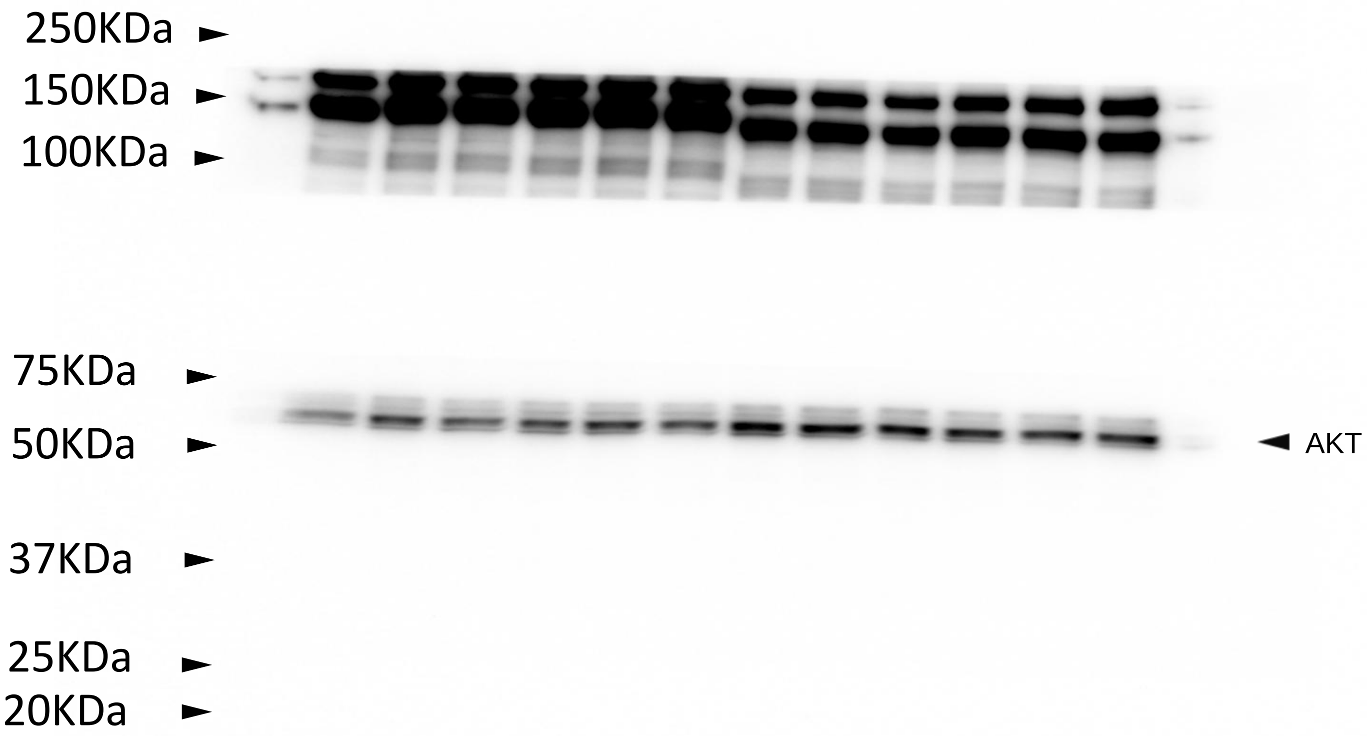

mTOR

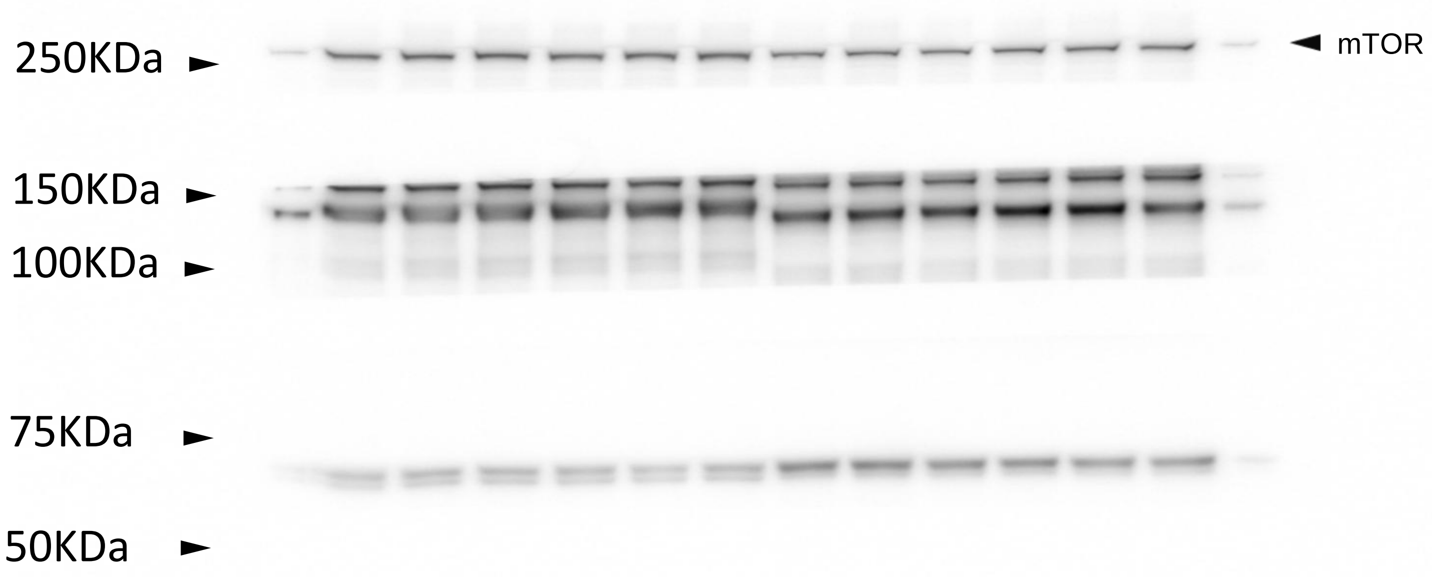

ERK1/2

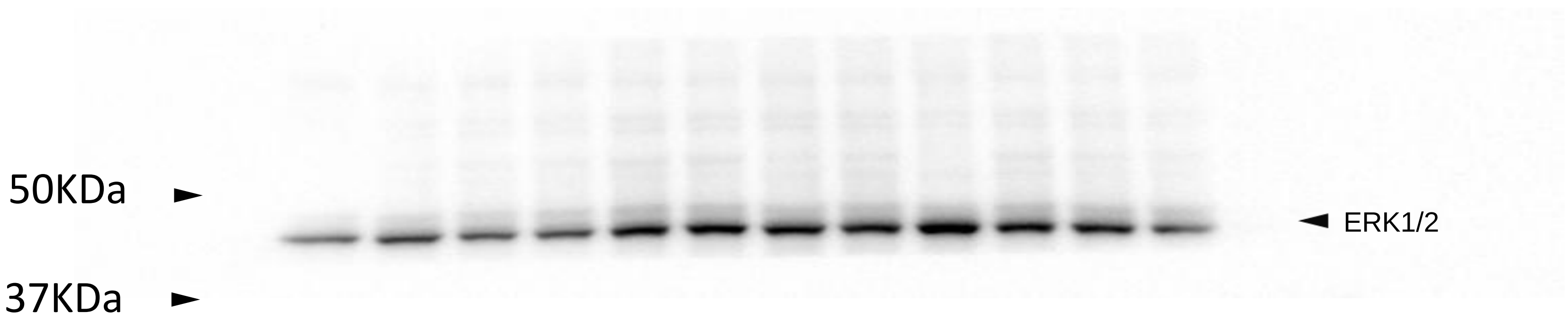

Vinculin

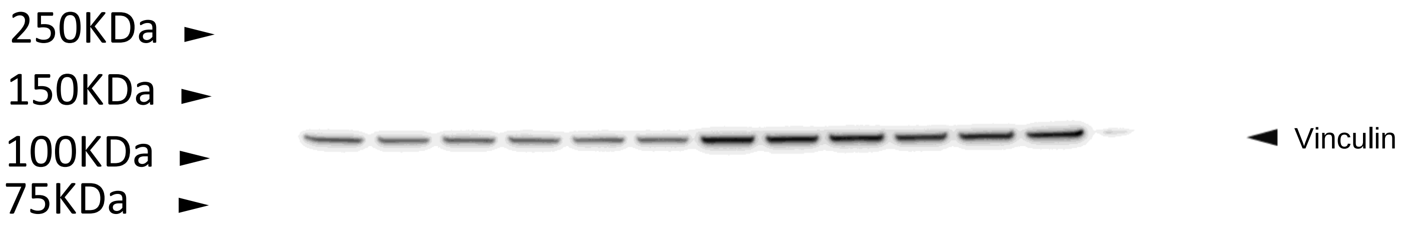

Supplement: Supplementary file 1 [file cancers-14-01378-s001.zip › Supplementary Figure S6 - Original western blots.zip!/Supplementary Figure S6 - Original western blots/(L) Supp. Fig S2.pdf]
